# Toward Recreating the Breath of Cities in the Atmospheric Simulation Chamber SAPHIR

**DOI:** 10.1021/acs.est.6c05767

**Published:** 2026-10-14

**Authors:** Yizhen Wu, Ralf Tillmann, Eva Y. Pfannerstill, Peeyush Khare, Andrea Carolina Marcillo Lara, Achim Grasse, Franz Rohrer, Milan Roska, Clémence Depp, Max Gerrit Adam, Sarah Albertin, Rabbia Asgher, Thomas J. Bannan, Shawon Barua, Kelvin H. Bates, David M. Bell, Birger Bohn, Steven Brown, Angela Buchholz, Yanfang Chen, Changmin Cho, Hugh Coe, Matthew M. Coggon, Michelle Färber, Sana Farhoudian, Juliane L. Fry, Hendrik Fuchs, Martin Graus, Imad El Haddad, Quanfu He, Thorsten Hohaus, Siddharth Iyer, Vlassis A. Karydis, Avinash Kumar, Markus Leiminger, Lu Liu, Emily Matthews, Gordon McFiggans, Ann M. Middlebrook, Markus Müller, Aki Nissinen, Anke C. Nölscher, Anna Novelli, Farhan R. Nursanto, Sebastien Perrier, André S. H. Prévôt, Iida Pullinen, Eka Dian Pusfitasari, Tobias Reinecke, Matti Rissanen, Matthieu Riva, Michael A. Robinson, Siegfried Schobesberger, Chelsea E. Stockwell, Jens Top, Alexandra P. Tsimpidi, Netta Vinkvist, Aristeidis Voliotis, Andreas Wahner, Yuantao Wang, Yuwei Wang, Carsten Warneke, Robert Wegener, Boxing Yang, Georgios I. Gkatzelis

**Affiliations:** † Institute of Climate and Energy Systems: Troposphere (ICE-3), 28334 Forschungszentrum Jülich GmbH, Jülich 52428, Germany; ‡ Institute of Geophysics and Meteorology, University of Cologne, 50969 Cologne, Germany; § 590435 NOAA Chemical Sciences Laboratory, Boulder, Colorado 80305, United States; ∥ Cooperative Institute for Research in Environmental Sciences, University of Colorado, Boulder, Colorado 80309, United States; ⊥ Aerosol Physics Laboratory, Physics Unit, Faculty of Engineering and Natural Sciences, 7840 Tampere University, 33720 Tampere, Finland; # Centre for Atmospheric Sciences, Department of Earth and Environmental Science, 5292 The University of Manchester, M13 9PL Manchester, U.K.; ∇ Department of Mechanical Engineering, 1877 University of Colorado Boulder, Boulder, Colorado 80309, United States; ○ PSI Center for Energy and Environmental Sciences, Paul Scherrer Institute, 5232 Villigen PSI, Switzerland; ◆ Department of Technical Physics, 163043 University of Eastern Finland, 70211 Kuopio, Finland; ¶ National Centre for Atmospheric Sciences, The University of Manchester, M13 9PL Manchester, U.K.; ⋈ Department of Environmental Sciences, 593528 Wageningen University, 6708 PB Wageningen, Netherlands; ⧓ Department of Physics, University of Cologne, 50923 Cologne, Germany; ⧖ IONICON Analytik GmbH, Eduard-Bodem-Gasse 3, 6020 Innsbruck, Austria; ● Univ. Lyon, Universite Claude Bernard Lyon1, CNRS, IRCELYON, 69626 Villeurbanne, France; ¤ Department of Chemistry, University of Helsinki, 00560 Helsinki, Finland

**Keywords:** Urban air quality, Volatile organic compounds, Radical chemistry, Oxidation products, Atmospheric simulation chambers, Emission inventories, Field−laboratory comparisons

## Abstract

Atmospheric simulation chambers are essential for studying oxidation processes, yet many experiments rely on single-precursor systems at concentrations that limit their representativeness for the chemical complexity of urban air. Here, we present a framework linking emission inventories, chamber experiments, and field observations by constructing emission fingerprints of major urban sources, including vegetation, volatile chemical products, cooking emissions, and gasoline and diesel exhaust. Applied during the CHANEL campaign in the SAPHIR outdoor chamber, these fingerprints advanced toward reproducing gas-phase mixtures representative of Los Angeles (gasoline-dominated traffic), a diesel-dominated city, and a future low-vehicle-emission scenario. The resulting mixtures generated peroxy-to-hydroperoxy radical ratios (RO_2_/HO_2_) close to unity, bimolecular RO_2_ lifetimes comparable with urban atmospheric observations, although lower NO levels favored RO_2_ + HO_2_ over RO_2_ + NO reactions. Comparison with aircraft measurements over Los Angeles using ammonium (NH_4_^+^) chemical ionization mass spectrometry showed reasonable agreement, with 465 organic product ions detected in both data sets, representing 78% of the product ions observed in the ambient air. Furthermore, 87% of common product ions agreed within a factor of 3 of the linear correlation, demonstrating that chamber experiments can capture key aspects of both atmospherically relevant radical chemistry and the oxidation product diversity of urban air.

## Graphical abstract

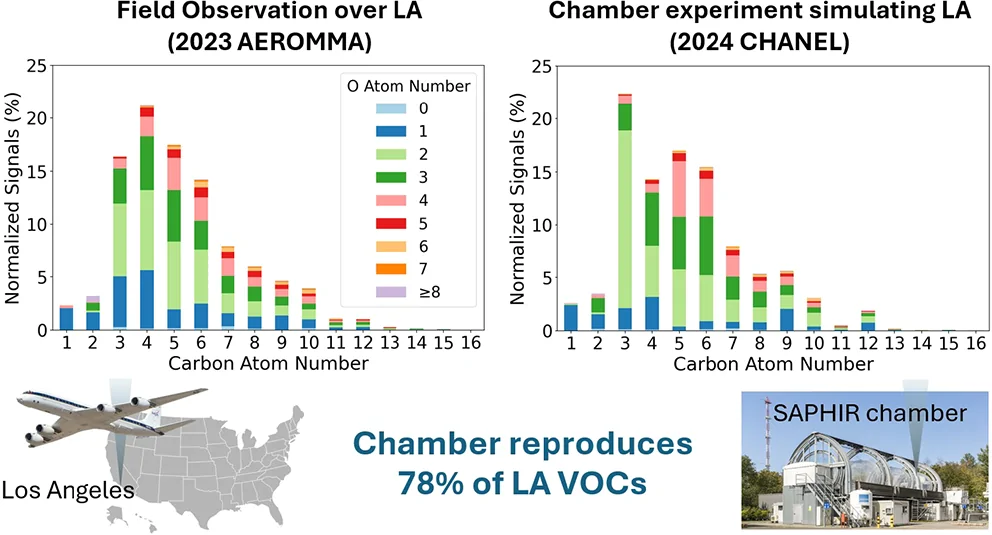

## 1 Introduction

Secondary organic aerosol (SOA) and ozone (O_3_) are major contributors to urban air pollution and its associated health burden, causing millions of premature deaths annually.^1–3^ Both pollutants are produced through the oxidation of volatile organic compounds (VOCs) and reactions involving nitrogen oxides (NO_*x*_),^4–6^ yet atmospheric chemical transport models still struggle to predict their abundance and variability in cities.^7^ This difficulty partly arises from the enormous chemical complexity of VOC emissions, which span thousands of compounds with widely different reactivity, volatility, and fates.^8^ While technological advances and regulations have substantially reduced vehicle-related VOC emissions in industrialized countries, the relative importance of other sources such as volatile chemical products (VCPs) and cooking activities has grown and reshaped the modern urban VOC mixture.^5,9–13^

Chamber studies have been indispensable for understanding VOC oxidation and SOA formation. Over decades, controlled experiments have revealed fundamental mechanisms of radical chemistry, product yields, and gas–particle partitioning.^4,14–26^ However, experiments have mostly been designed around single precursors^15,16,26^ or direct emissions from a single well-defined source, e.g., direct gasoline vehicle exhaust^24,25^ or direct plant emissions.^17,18^ Carter et al. pioneered the use of VOC mixtures to more accurately simulate the chemical complexity of urban air, successfully demonstrating the benefits of capturing mixture-driven O_3_ formation. Nevertheless, the chemical complexity in those experiments was inherently constrained by the number of species included, and experiments were often conducted under conditions diverging from the dilute, low-NO regimes typical of the ambient atmosphere.^27^ Organic peroxy radicals (RO_2_), formed from VOC oxidation, react with nitrogen monoxide (NO), hydroperoxyl radicals (HO_2_), other RO_2_, or nitrogen dioxide (NO_2_), or through unimolecular isomerization. The NO channel propagates radicals and yields O_3_ and organic nitrates, whereas the HO_2_ channel terminates them and yields hydroperoxides, so the prevailing NO level governs the product distribution and, through gas–particle partitioning, the formation of SOA.^28^ Indeed, prior work has shown that RO_2_ fates and NO levels in many chamber experiments deviate from those observed in the atmosphere,^28,29^ limiting the applicability of chamber-derived SOA yields in models.

Several studies focusing on gas-phase chemistry have also achieved more atmospherically relevant radical regimes,^30–38^ but these experiments typically relied on single compounds or a limited number of precursors. While such simplified experimental conditions provide valuable chemical mechanism insights, they do not capture the diversity of real-world ambient air, where multiple emission sources interact simultaneously. Under these complex conditions, highly reactive VOCs can scavenge hydroxyl radicals (OH), alter RO_2_ radicals reaction pathways, and thereby influence SOA and O_3_ production efficiency,^6^ making direct comparisons between chamber experiments and field observations challenging.

Here we introduce a new framework that directly links VOC emission inventories, chamber experiments, and field observations. Drawing on prior knowledge from single-component oxidation studies, field observations, and emission inventories, we developed reduced-complexity chemical fingerprints of VOCs from VCPs,^10^ cooking,^11^ gasoline tailpipe,^5,10,39,40^ and diesel tailpipe^5,10,39,40^ (hereafter gasoline and diesel) emissions, which together represent the dominant anthropogenic VOC sources in summertime modern cities. During the CHANEL (Household Chemicals Amplifying Urban Aerosol Pollution) campaign in 2024, these fingerprints were combined with biogenic emissions of Los Angeles (LA)^41^ to produce city-like mixtures and were injected into the SAPHIR (Simulation of Atmospheric PHotochemistry In a large Reaction Chamber) outdoor chamber.^42,43^ Exposure to natural sunlight during daytime and to oxidant injection under closed-roof conditions at night enabled atmospherically relevant oxidation chemistry across both regimes.

To our knowledge, this is the first study to introduce complex, inventory-based urban VOC mixtures in an atmospheric simulation chamber. Comparison of the resulting radical chemistry against atmospheric radical lifetime distributions^28^ from the JULIAC (Jülich Atmospheric Chemistry)^44^ and AEROMMA (Atmospheric Emissions and Reactions Observed from Megacities to Marine Areas)^45,46^ field campaigns indicates that the CHANEL experiments approach, without fully matching, the radical regimes observed in ambient air. Furthermore, an experiment designed to simulate LA urban air captured 78% of the product ions observed by aircraft over LA during the AEROMMA campaign. Together, these results show that controlled chamber experiments move closer to replicating the gas-phase chemical ″breath of cities″, providing a new bridge between laboratory studies and real-world urban atmospheres. This study focuses on gas-phase chemistry; SOA formation potential was nonetheless incorporated as a fingerprint selection criterion alongside OH reactivity, ensuring that precursors relevant to particle formation are retained and laying the groundwork for future particle-phase investigations using the same framework.

## 2 Methods

### 2.1 Determination of Emission Inventory Profiles for Urban Sources

Emission profiles for VCPs, gasoline, diesel, and cooking were derived from the GRA^2^PES inventory,^5,10,11,39,40,47^ which represents urban emissions in the United States and has been widely integrated into regional models, successfully reproducing observed variability in O_3_ and VOCs in US cities.^5,48,49^

For each VOC in the inventory, OH rate constants (*k*_OH+X_) were compiled from the literature,^50–56^ and SOA formation potential units (SOAPu) were assigned following Cappa and Wilson, based on VOC class, carbon number, and oxygen atom number.^57^ The fractional contributions of each compound to the total VOC OH reactivity (hereafter OH reactivity) and the total SOA formation potential were then calculated as

1 $$Fractional\,\;OH\,\;reactivity\left(\%\right)=\left[mole\,\;fraction_{i}\times k_{OH_{i}}\right]/\sum [mole\,\;fraction_{i}\times k_{OH_{i}}]\times 100$$

2 $$\mathrm{Fr}actional\,\;SOA\,\;formation\,\;potential\left(\%\right)=\left[mass\,\;fraction_{i}\times {SOAPu}_{i}\right]/\sum [mass\,\;fraction_{i}\times {SOAPu}_{i}]\times 100$$

where mole fraction and mass fraction denote the fractional contributions of each VOC to the total emitted moles and total emitted mass of the inventory source profile, respectively. These fractional metrics are used solely for ranking and fingerprint generation and should not be confused with absolute OH reactivity (s^–1^) or absolute SOA formation potential (μg/μg).

Urban VOC emissions comprise thousands of reactive chemical compounds from diverse sources, making it challenging to isolate source-specific oxidation pathways in controlled chamber experiments. We therefore reduced the chemical complexity of each source while retaining the dominant precursors that characterize its key chemical properties — preserving the reactivity and SOA formation potential of the full emission profile while keeping the system tractable for mechanistic investigation. To this end, we developed reduced-complexity chemical “fingerprints” of VOC emissions from VCPs, cooking, gasoline, and diesel emissions using a four-step procedure summarized in Table 1 and Figure S1, with a detailed description in the .

**Table 1:** Overview of the Four-Step Procedure Used to Generate Reduced-Complexity Fingerprints for Urban VOC Sources

step	description
1. Ranking by contribution	VOCs in each emission inventory were ranked by their contributions to the total OH reactivity and the SOA formation potential (eqs 1 and 2). Starting from the highest-ranked contributors, species were retained until 80% of each metric was captured.
2. Surrogate definition	where inventories reported compounds only as chemical classes or unresolved structures, representative surrogate species were selected based on established approaches or by identifying compounds with the same molecular formula reported in measurements.^58,59^
3. Classification and lumping	compounds were assigned to the Community Regional Atmospheric Chemistry Multiphase Mechanism (CRACMM) chemical classes.^58^ Within each class, compounds with comparable OH rate constants and SOA formation potential units were aggregated, with the highest-emitted compound serving as the representative.
4. Substitution	compounds not commercially available during the CHANEL campaign were replaced with structurally similar surrogates of comparable OH rate constants and SOA formation potential units.

To decrease uncertainties introduced by this method, OH reactivity and SOAP were recalculated using the reduced-complexity fingerprints and compared with the corresponding values from the full emission inventories (Figures 1 and S3). Detailed compound lists and surrogate choices are provided in Tables S1–S9 and the Supporting spreadsheet.

**Figure 1:**
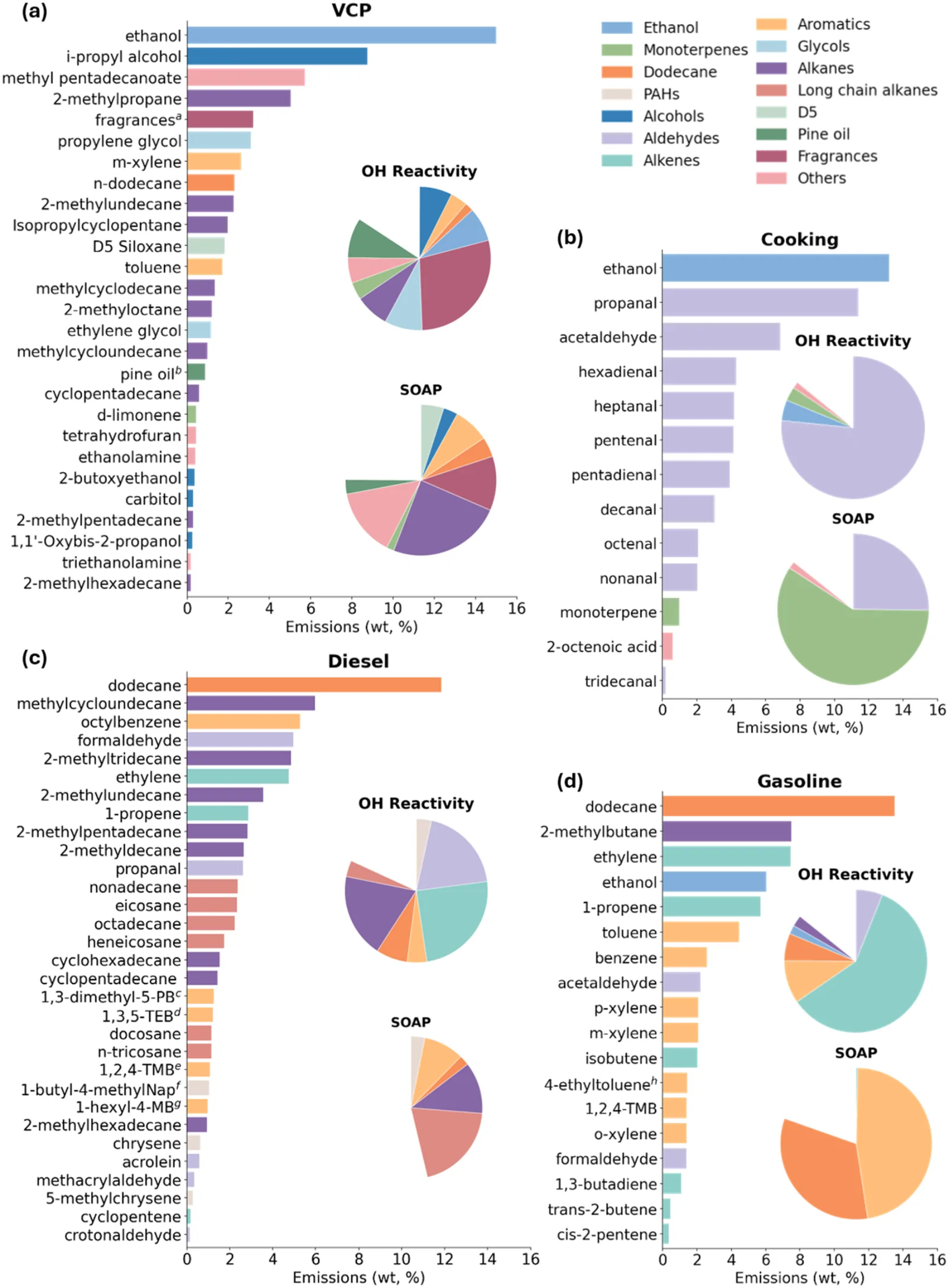
Reduced chemical fingerprints of major urban emission sources including (a) volatile chemical products (VCP), (b) cooking, (c) diesel tailpipe, and (d) gasoline tailpipe emissions. Bars show individual compound contributions to total source mass (by weight), and pie charts indicate the fractions of OH reactivity and secondary organic aerosol formation potential (SOAP) retained relative to the full emission inventories.^5,10,11,39,40,47^ In the figure, ^a^ fragrances denotes mixtures consisting of 20% monoterpenes and 80% propylene glycol; ^b^ pine oil denotes a mixture of 25% a-pinene, 25% b-pinene, and 50% limonene; ^c^ 1-butyl-4-methylNap denotes 1-butyl-4-methylnaphthalene; ^d^ 1,3,5-TEB denotes 1,3,5-triethylbenzene; ^e^ 1,2,3-TMB denotes 1,2,3-trimethylbenzene; ^f^ 1-hexyl-4-MB denotes 1-hexyl-4-methylbenzene; ^g^ 1,3-dimethyl-5-PB denotes 1,3-dimethyl-5-propylbenzene; and ^h^ 4-ethyltoluene represents either 4-ethyltoluene or 3-ethyltoluene.

With these fingerprints established, we conducted 27 chamber experiments during the CHANEL campaign, encompassing both single-source simulations and multisource mixtures designed to replicate different summertime urban environments. Los Angeles was selected as a primary reference city because of the availability of extensive gas- and particle-phase observations for direct comparison, as well as the extensive validation of the GRA^2^PES inventory in this region.^5,41^ In addition, we designed experiments to approximate representative European or industrialized urban conditions, where diesel emissions contribute more strongly to the traffic-related VOC mixture (∼73%) compared to US cities (∼25%).^60^ Future urban scenarios were also explored by reducing traffic-related emissions, reflecting expectations associated with ongoing energy transition and electrification. For VCPs, emission inventories outside the US remain limited. However, available studies indicate that VCP emissions scale with population density and that their relative contribution is not expected to differ dramatically between the US and Europe,^48,61^ though regional differences are expected, particularly in rapidly developing or industrializing regions.^62^ Based on these constraints, the global urban mixtures were constructed to represent moderately populated European cities rather than highly dense megacities such as Paris or New York.

### 2.2 The Atmospheric Simulation Chamber SAPHIR

In the summer of 2024, the CHANEL campaign experiments were performed in the SAPHIR chamber at Forschungszentrum Jülich, Germany. The SAPHIR chamber made of double-wall Teflon film has a volume of approximately 270 m^3^, with a low surface-to-volume ratio of around 1 m^–1^, which minimizes wall effects. The chamber is equipped with a shutter system that can be opened to allow the chamber air to be exposed to natural sunlight.^63,64^ OH and NO are produced via photolysis of nitrous acid (HONO), which originates from the Teflon surface upon illumination in the presence of water.^42^ Synthetic air is generated by evaporating high-purity liquid N_2_ and O_2_ (purity >99.9999%) and was used to flush the SAPHIR chamber between experiments to ensure the experiment started at clean conditions. Chamber humidity was controlled by evaporating high-purity water (Milli-Q Gradient A10, Millipore Corp.) and flushing the vapor into the chamber with a large flow of synthetic air (150 m^3^/h) until the target humidity level was reached. Further details about the SAPHIR chamber can be found in earlier publications.^42,43^

### 2.3 Experimental Procedure

Among the 27 experiments of the SAPHIR CHANEL campaign, there were single-compound simulations using limonene as a reference, single-source simulations (VCPs, cooking, gasoline, diesel), and multisource simulations representing present-day US, European cities, and future low- vehicle-emission scenarios (Table S10). Limonene was chosen as the single-compound reference because it is a monoterpene emitted from both biogenic sources and anthropogenic sources such as VCPs, and because its oxidation chemistry is more complex and less fully constrained than that of most other monoterpenes, making it both a widely characterized benchmark and a scientifically important system in its own right. Across all scenarios, emissions from different source categories were introduced simultaneously, representing conditions typical of a well-mixed urban boundary layer during daytime, when emissions from traffic, cooking, and VCPs co-occur and undergo oxidation under shared radical and oxidant environments (Tables S1–S9). Experiments spanned a range of NO_*x*_ conditions and oxidant regimes, including daytime oxidation dominated by OH and O_3_, nighttime oxidation dominated by O_3_ and NO_3_, and pure ozonolysis experiments, as summarized in Table S10. Nighttime experiments were designed to simulate the oxidation of VOCs emitted during the preceding evening rush hour and subsequently accumulated in the atmosphere, rather than to represent concurrent nighttime emission rates.

Each experiment began with humidification of the chamber to approximately 60% relative humidity at around 20 °C, followed by injection of about 10 μg m^–3^ of ammonium sulfate seed particles. Liquid VOCs were introduced through a heated, inert-coated injection line and vaporized into a high-flow stream of synthetic air, while high-volatility gases were added directly from pressurized cylinders and homogenized by the chamber fans within a few minutes. Detailed injection protocols, chamber conditions, and compound-specific injection efficiencies are given in Texts S5–S6, Tables S11–S12, and Figures S4–S6.

Oxidation conditions were varied by adjusting NO, NO_2_, O_3_, and CO levels and by opening or closing the chamber roof. High-NO experiments received 7.5 ppbv of NO, or 8 ppbv of NO_2_ in the Los Angeles simulation (Experiment 24), giving average NO mixing ratios of 0.23 to 1.05 ppbv. Medium-NO experiments relied solely on NO formed by photolysis of HONO from the chamber surface, averaging 0.15 to 0.31 ppbv. Low-NO experiments used 50 to 100 ppbv of O_3_ to titrate the NO formed in the chamber, averaging 0.07 to 0.19 ppbv (Table S11). Daytime experiments were run with the roof open so that HONO photolysis generated OH, whereas nighttime experiments were run with the roof closed after injection of O_3_ (10 to 40 ppbv) and NO_2_ (17 to 40 ppbv) to form NO_3_. Separate experiments used 75 ppbv of O_3_ for pure ozonolysis or 2 to 60 ppmv of CO to scavenge OH.

Average concentrations of NO, NO_2_, and NO_*x*_, as well as average temperature and relative humidity of each experiment are shown in Table S11. Representative time series of oxidants, radicals, and SOA in daytime and nighttime experiments are shown in Figures S4 and S5.

### 2.4 Instrumentation

The CHANEL campaign employed an extensive suite of instruments measuring gas-phase composition, aerosol properties, and radical concentrations, a full overview of which is provided in Text S4. The present analysis focuses on a subset of instruments that were central to the radical chemistry and VOC diversity analyses presented here, and that were also deployed during recent urban field campaigns, most notably AEROMMA, enabling direct chamber-to-field comparisons. Specifically, we describe the NH4^+^ VOCUS mass spectrometer (VOC measurements), the ROxLIF instrument (radical measurements), and the Ecophysics CLD-780 TR (NO and NO_2_ measurements). Measurements from additional instruments, including those used to characterize injection efficiency and aerosol chemical composition, are discussed in the Supporting Information.

#### 2.4.1 VOC Measurements

A Tofwerk VOCUS long time-of-flight mass spectrometer (Tofwerk AG, Switzerland) was employed in the SAPHIR chamber, operated with ammonium reagent ions (NH_4_^+^) to measure VOCs and oxygenated VOCs (OVOCs). Detailed descriptions of the instrument’s operating principles and setup can be found in Krechmer et al.^65^ and Xu et al.^66^

Instrument backgrounds were measured for 5 min every hour, and daily calibrations with a certified 20-compound VOC gas mixture showed stable sensitivity throughout the campaign. Data were acquired at a time resolution of 1 Hz, and only ions whose signals exceeded background levels by at least 4 standard deviations for more than 20% of the experiment duration were retained for analysis. Ion source settings, inlet configuration, sensitivities, and data processing are described in Text S4.1.

For comparison with ambient observations, measurements from the NH_4_^+^ VOCUS instrument deployed on the NOAA aircraft during the AEROMMA campaign^67^ were also used. The instrument operated with the same ionization scheme as in SAPHIR, and followed the same operating principles described above. Ionization product distributions of common calibrants agreed closely between the two campaigns (Text S2 and S4). A detailed characterization of the VOCUS performance during AEROMMA is provided by Roska et al.^68^ Because the mass spectrometer itself does not provide structural information, the comparison of VOCs between the CHANEL campaign and the AEROMMA field measurement in this study is limited by its inability to separate isomers.

Besides VOCUS, an online thermal desorption gas chromatography system coupled to dual flame ionization and mass spectrometric detection (GC-TD-FID/MS; Markes TT24–7xr with Kori-xr units, Agilent 8890 GC, Agilent 5977B MS) was used for nonmethane hydrocarbons (NMHCs; C_2_–C_12_) measurements and a FUSION PTR-TOF for aromatic and oxygenated compounds (Text S4).

#### 2.4.2 Measurements of Radicals

OH, HO_2_, and RO_2_ radicals were measured with a laser-induced fluorescence (LIF) instrument (ROxLIF).^69,70^ OH was detected by laser-induced fluorescence in a low-pressure cell. In the CHANEL campaign, the OH cell was equipped with a chemical modulation reactor (CMR),^71^ where propane was periodically injected to scavenge ambient OH, separating it from potentially internally produced radicals and enabling interference-free OH measurements.^71^

HO_2_ was monitored in a second cell by conversion to OH via reaction with added NO, so that the sum of HO_2_ and OH radicals were detected, and HO_2_ concentrations were derived by subtracting the OH signal.^69^ Potential interferences from certain RO_2_ species, which rapidly generate HO_2_ through RO_2_ + NO, were negligible as the HO_2_ detection cell was operated at low NO levels in this study.^72^

Total RO_*x*_ (OH + HO_2_ + RO_2_) concentrations were determined in a third measurement cell. In a converter, RO_2_ was converted stepwise to HO_2_ and then to OH by reaction with added NO in the presence of excess CO.^69,73,74^ RO_2_ concentrations were derived from the difference between total RO_*x*_ and the separately measured OH and HO_2_.

NO-related background signals in the HO_*x*_ and RO_*x*_ detection cells were characterized during regular calibrations. The exact cause of this NO-related background remains unresolved. Ten calibrations conducted throughout the campaign showed stable sensitivity (<15% drift). This background was subtracted from the measured HO_*x*_ and RO_*x*_ signals; because radical concentrations observed during CHANEL were generally well above 1 × 10^8^ cm^–3^, the impact of this background subtraction on the reported concentrations is minor. Average radical concentrations of each experiment are shown in Table S12.

#### 2.4.3 Measurements of NO and NO_2_

NO and NO_2_ were measured by the Ecophysics CLD-780 TR which operates on the principle of chemiluminescence detection.^75^ Calibration was conducted using a gas-phase titration system (Text S4). In NO mode, a primary NO standard (2.048 ± 0.016 μmol mol^–1^) was diluted with zero air, while in NO_2_ mode, excess ozone was added to the NO standard to quantitatively generate NO_2_.

### 2.5 RO_2_ Fate Analysis

RO_2_ radicals formed during VOC oxidation can undergo several competing reactions, including bimolecular reactions with NO, HO_2_, RO_2_, and NO_2_, as well as unimolecular isomerization reactions. To evaluate these pathways in SAPHIR, we followed the framework of Kenagy et al.^28^ and use three parameters: β, τ_bi_, and RO_2_/HO_2_.

β describes the competition between RO_2_ + NO and RO_2_ + HO_2_ reactions, and is defined as

3 $$\beta =\frac{k_{{RO}_{2}+NO}\left[NO\right]}{k_{{RO}_{2}+NO}\left[NO\right]+k_{{RO}_{2}+{HO}_{2}}\left[{HO}_{2}\right]}$$

A proxy of the bimolecular lifetime of RO_2_ against reactions with NO and HO_2_, τ_bi_, is given by

4 $$\tau_{bi}=\frac{1}{k_{{RO}_{2}+NO}\left[NO\right]+k_{{RO}_{2}+{HO}_{2}}\left[{HO}_{2}\right]}$$

where *k*_RO_2_+NO_ and *k*_RO_2_+HO_2__ are the bimolecular rate constants for RO_2_ reacting with NO and HO_2_, respectively. [NO] and [HO_2_] are the measured concentrations of NO and HO_2_. For the AEROMMA campaign, β was estimated using first-generation isoprene oxidation product measurements, and τ_bi_ was estimated from NO measurements and modeled HO_2_ as described in Robinson et al.^46^

Finally, the RO_2_/HO_2_ ratio is used as a proxy to assess the relative importance of RO_2_ + RO_2_ and RO_2_ + HO_2_ reaction pathways, which terminate the radical reaction cycle.

## 3 Results and Discussion

### 3.1 Reduced-Complexity Emission Fingerprints

Reconstructing the chemical complexity of urban air in chamber experiments requires a manageable yet atmospherically representative set of precursors. The methodology described in Section 2.1 yields reduced-complexity fingerprints, converting hundreds of VOCs from inventories into tractable sets of fewer than 30 species per source while retaining the dominant contributors to OH reactivity and SOA formation potential (Figure 1).

Each source exhibits distinct drivers of OH reactivity and SOA formation. VCP emissions are dominated by mass by alcohols, yet it is hydrocarbons, esters, aromatics, and fragrances (20% monoterpenes, 80% propylene glycol) that drive SOAP, while fragrances, alcohols, glycols, and pine oil (25% α-pinene, 25% β-pinene, and 50% limonene) collectively dominate OH reactivity. In contrast, gasoline emissions are dominated by aliphatic and aromatic compounds, where light alkenes (e.g., propene, isobutene, ethylene) control the total OH reactivity, and aromatics and dodecane dominate SOAP. Diesel emissions shift toward long-chain alkanes such as n-dodecane and higher homologues, with aldehydes and alkenes driving OH reactivity and alkanes and aromatics shaping SOAP. Cooking emissions present a different profile, rich in ethanol and aldehydes that dominate total OH reactivity, while monoterpenes account for more than half of the SOAP and decanal comprises most of the remainder, reflecting the outsized SOA potential of just a few compounds in this source class.

The reduced-complexity fingerprints explain more than 75% of the source OH reactivity and the SOAP across all sources, indicating that the lumping approach preserves the key chemical characteristics, except diesel, where roughly half of the SOAP is represented. This reduced coverage reflects increased uncertainties in the diesel emission inventories, where unresolved complex mixtures account for ∼35% of the SOAP. Despite recent advances in characterizing C_10_-C_18_ hydrocarbons from diesel exhaust,^76–79^ substantial fractions of semivolatile and oxygenated species still remain poorly constrained and dominate the SOAP fraction.^47^ As a result, the diesel fingerprint represents a conservative lower bound for diesel-derived SOA formation potential, while still effectively capturing the major reactive compound classes.

For VCPs, the methodology itself preserves approximately 80% of the SOA formation potential. However, due to limited commercial availability of several key compounds (e.g., methylcycloundecane and methylcyclodecane), the effective VCP representation in the CHANEL experiments was reduced to roughly 50% of the inventory-based SOAP (Figure S3). Consequently, similar to diesel, the VCP injections used here underrepresent the full SOA-forming capacity of this source class, even though this limitation arises from experimental constraints rather than from the fingerprint framework itself.

Taken together, these constraints define the overall uncertainty of the method. The fingerprints reproduce 50 to 80% of the inventory-based OH reactivity and SOAP, injection efficiency adds further variability, and unresolved IVOCs and SVOCs, which are known to contribute substantially to SOA formation, are not fully represented. Notably, the compounds contributing most to the missing SOAP tend to be less reactive, longer-lived species that are more susceptible to deposition losses in the real atmosphere,^80^ their exclusion may therefore affect SOA mass yield comparisons less than the nominal SOAP gap suggests. The fingerprints are therefore best understood as a first-order representation of urban emissions, one that conserves the relative composition and reactivity distribution of each source class rather than its absolute mass loading. With these fingerprints established, we conducted 27 chamber experiments simulating a range of urban environments. These experiments captured co-occurring emissions from traffic, cooking, and VCPs under diverse oxidant and radical regimes, allowing the chemical complexity of urban oxidation products to be approached under controlled conditions.

### 3.2 Radical Chemistry Regimes in the SAPHIR Experiments

The competition between RO_2_ reactions with NO, HO_2_, and other RO_2_ radicals, as well as unimolecular isomerization reactions determine the efficiency of SOA and O_3_ production, and the distribution of oxidation products.^6,28,29,34^ To reproduce atmospheric conditions in chamber experiments, it is vital that the fate of RO_2_ radicals follows what is observed in ambient.

Figure 2 shows RO_2_ fates during the CHANEL experiments using three diagnostic metrics following Kenagy et al.^28^ The parameter β (eq 3) describes the competition between RO_2_ + NO and RO_2_ + HO_2_, τ_bi_ (eq 4) represents the bimolecular lifetime of RO_2_ against reactions with NO and HO_2_, and the RO_2_/HO_2_ concentration ratios characterize the relative importance of RO_2_ + RO_2_ versus RO_2_ + HO_2_ pathways (Section 2.5). The β – τ_bi_ and β – RO_2_/HO_2_ distributions observed in CHANEL experiments overlapped strongly with the range of conditions predicted by the GEOS-Chem chemical transport model used in Kenagy et al.,^28^ which provides a broad estimate for atmospherically relevant RO_2_ fates across diverse chemical environments (Figure 2, gray outlined oval). Because that study did not explicitly separate environments such as urban versus rural or daytime versus nighttime, the model distributions serve here as a contextual benchmark rather than a location-specific target.

**Figure 2:**
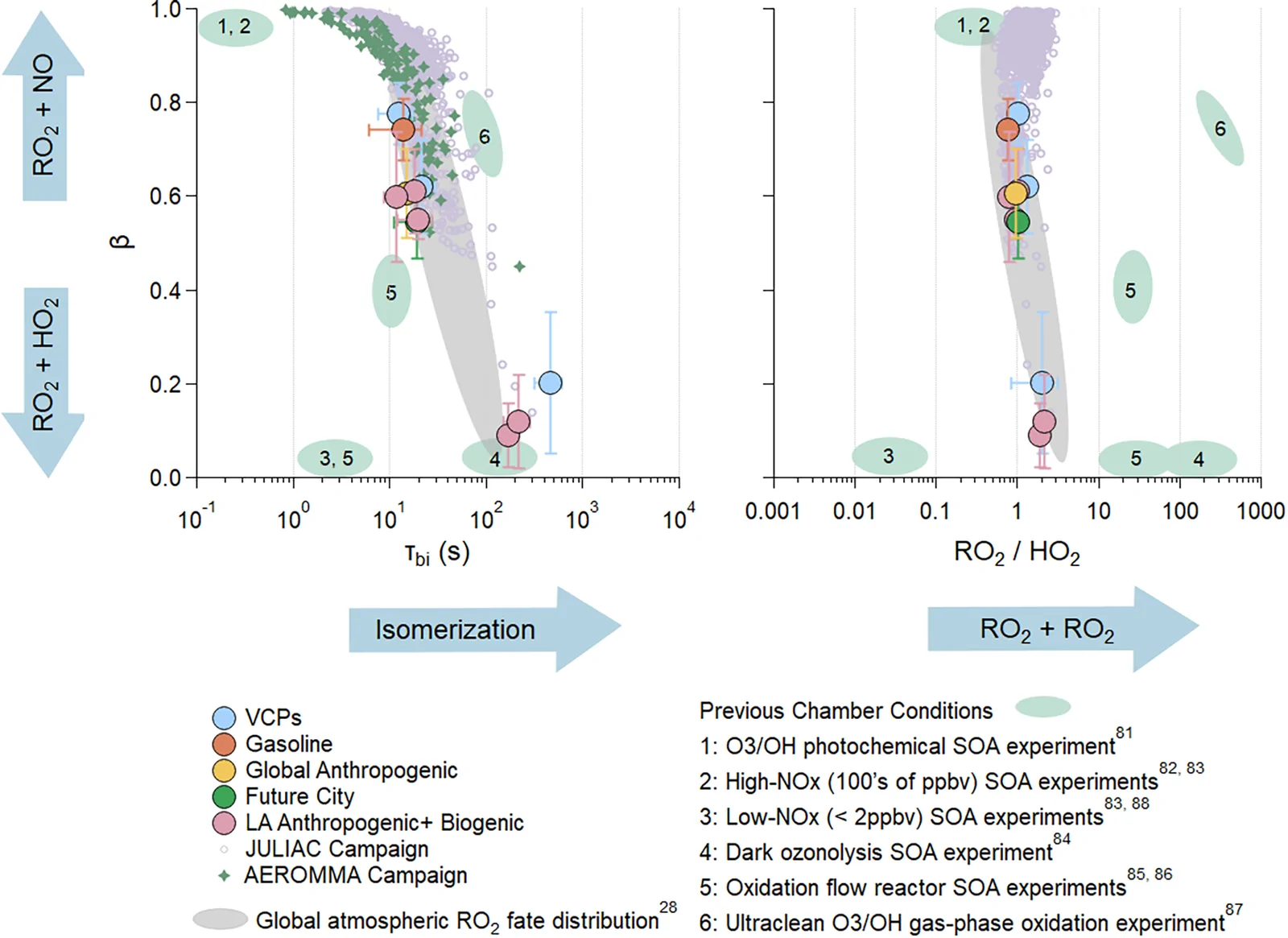
Radical termination reaction regimes of CHANEL experiments compared with global atmospheric distributions derived from Kenagy et al.^28^ (gray oval), JULIAC field observations (green stars), and AEROMMA airborne observations (purple circles). Colored markers denote different injection mixtures (Tables S1–S9), with error bars indicating standard deviations. The turquoise ovals indicate the ranges of previous chamber studies.^81–88^

Within this benchmark, CHANEL experiments occupy regions of RO_2_ fate space that are representative of ambient photochemical conditions, in contrast to many prior chamber experiments reported by Kenagy et al. (Figure 2, turquoise ovals with numbers).^81–88^ Those studies, many of which were designed specifically to maximize SOA formation and often employed oxidation flow reactors, clustered in more restricted regions characterized by extreme RO_2_ + NO or RO_2_ + HO_2_ dominance, unrealistically short RO_2_ lifetimes, and RO_2_/HO_2_ ratios varying by orders of magnitude.^81–83,88^ Against this broader context, CHANEL spans a substantially wider and more atmospherically relevant range of RO_2_ fate conditions, benefiting from reduced wall effects due to SAPHIR’s high volume-to-surface ratio, and operating at atmospherically relevant precursor mixtures, thereby enabling photochemical regimes that closely resemble those of the real atmosphere.

We further evaluated representativeness by comparing CHANEL radical regimes with ambient observations from the JULIAC campaign^44^ (Text S3) and airborne observations over multiple US cities during the AEROMMA campaign^46^ (Text S2). CHANEL experiments successfully reproduce the range of modeled global τ_bi_ and RO_2_/HO_2_ ratios in Kenagy et al.^28^ In contrast, ambient air observations in the JULIAC and AEROMMA campaigns generally exhibited higher β values, reflecting a stronger dominance of RO_2_ + NO pathways. These differences arise from a combination of higher NO levels in the ambient air observations compared to most low- and medium-NO CHANEL experiments and elevated HO_2_ concentrations in CHANEL (Figure S7) associated with fresh, highly reactive precursor mixtures typical of chamber studies.

The origin of these differences is consistent across both campaigns. JULIAC sampled chemically aged and diluted air from a rural region influenced by surrounding cities, continuously introduced into SAPHIR under steady-state conditions.^44^ This setup combined elevated NO availability from regional transport with additional NO from HONO photolysis from the SAPHIR chamber walls,^42^ favoring higher β regimes. Similarly, AEROMMA sampled fresh urban plumes with strong anthropogenic NO emissions, with average NO levels around 1 ppbv,^46^ substantially higher than those in most CHANEL experiments— a difference that may also have limited organic nitrate formation in the chamber relative to ambient air. In CHANEL, higher total precursor reactivity in multisource experiments elevated absolute radical concentrations, shifting β toward lower values despite similar RO_2_/HO_2_ ratios. The few high-NO*_x_* CHANEL experiments did reach high NO levels (Table S11) comparable to AEROMMA, but direct verification is not possible due to the lack of concurrent radical measurements.

Consistent with this interpretation, CHANEL experiments exhibit higher absolute VOC, RO_2_, and HO_2_ turnover rates than JULIAC by roughly a factor of 2 (Figure S8), reflecting accelerated radical cycling driven by fresh, reactive emissions typical of chamber studies. In contrast, JULIAC air masses were relatively clean with low concentrations of anthropogenic and biogenic VOCs,^44^ resulting in weaker overall radical cycling. Despite these differences in absolute rates, the similarity in RO_2_/HO_2_ ratios and τ_bi_ values indicates that CHANEL comes closer than previous chamber studies to reproducing atmospherically realistic radical termination pathways.

### 3.3 Comparison with Los Angeles Observations (AEROMMA)

To evaluate how closely CHANEL mixtures approach the oxidation product complexity of real-world urban environments, we compared VOCs measured by an NH_4_^+^ VOCUS instrument during the CHANEL campaign (Section 2.4.1 and S4.1) with VOCs observed by the NOAA NH_4_^+^ VOCUS instrument onboard the NASA DC-8 aircraft over Los Angeles during the AEROMMA campaign (Text S2).^45^ Briefly, NH_4_^+^ chemical ionization is a softer ionization technique that is well suited for measuring oxidation products due to its well-suited sensitivity to oxygenated organic compounds including ketones, aldehydes, alcohols, some acids, and organic nitrates, but has limited sensitivity to nonoxygenated aromatics.^66^

For consistency, VOCs directly injected in each CHANEL experiment were excluded from both data sets, as were compounds with substantial ambient background contributions such as acetone, ethanol, and acetonitrile. Noninjected VOCs that could represent primary emissions were retained in both data sets. The comparison focused on VOCs measured during the final 2 h of each CHANEL experiment, representing predominantly secondary oxidation products, and measurements in the boundary layer over Los Angeles during AEROMMA, hereafter referred to as LA urban air.

The two data sets also differ in meteorological conditions. In the experiment designed to simulate present-day LA conditions (experiment 24), average relative humidity and temperature were 21 ± 6% and 36 ± 3 °C, respectively, compared with 41 ± 16% and 25 ± 2 °C during AEROMMA, and the average photolysis frequency of NO_2_ (*J*_NO2_) was approximately half that observed during AEROMMA [(4.15 ± 1.05) × 10^–3^ s^–1^ versus (7.82 ± 1.81) × 10^–3^ s^–1^]. Given these differences in location and meteorological conditions, some compositional discrepancies are expected and do not by themselves indicate limitations of the fingerprinting approach.

#### 3.3.1 Overall Overlap between CHANEL Experiments and LA Observations

Figure 3e shows the fraction of the 597 product ions detected in the Los Angeles urban boundary layer during AEROMMA that were also detected in each CHANEL experiment. The experiment designed to simulate present-day LA conditions, which included fingerprints of VCPs, gasoline, cooking, and biogenic emissions^41^ (Experiment 24, hereafter CHANEL LA simulation), achieved the highest overlap with AEROMMA observations. Of the 597 product ions detected in LA urban air, 465 were also observed in the CHANEL LA simulation, corresponding to 78% of the ambient product ions measured (Figure 3e). This represents, to our knowledge, the most extensive chamber–field overlap reported to date, extending beyond individual tracers to several hundred VOCs and oxygenated VOCs.^27^

**Figure 3:**
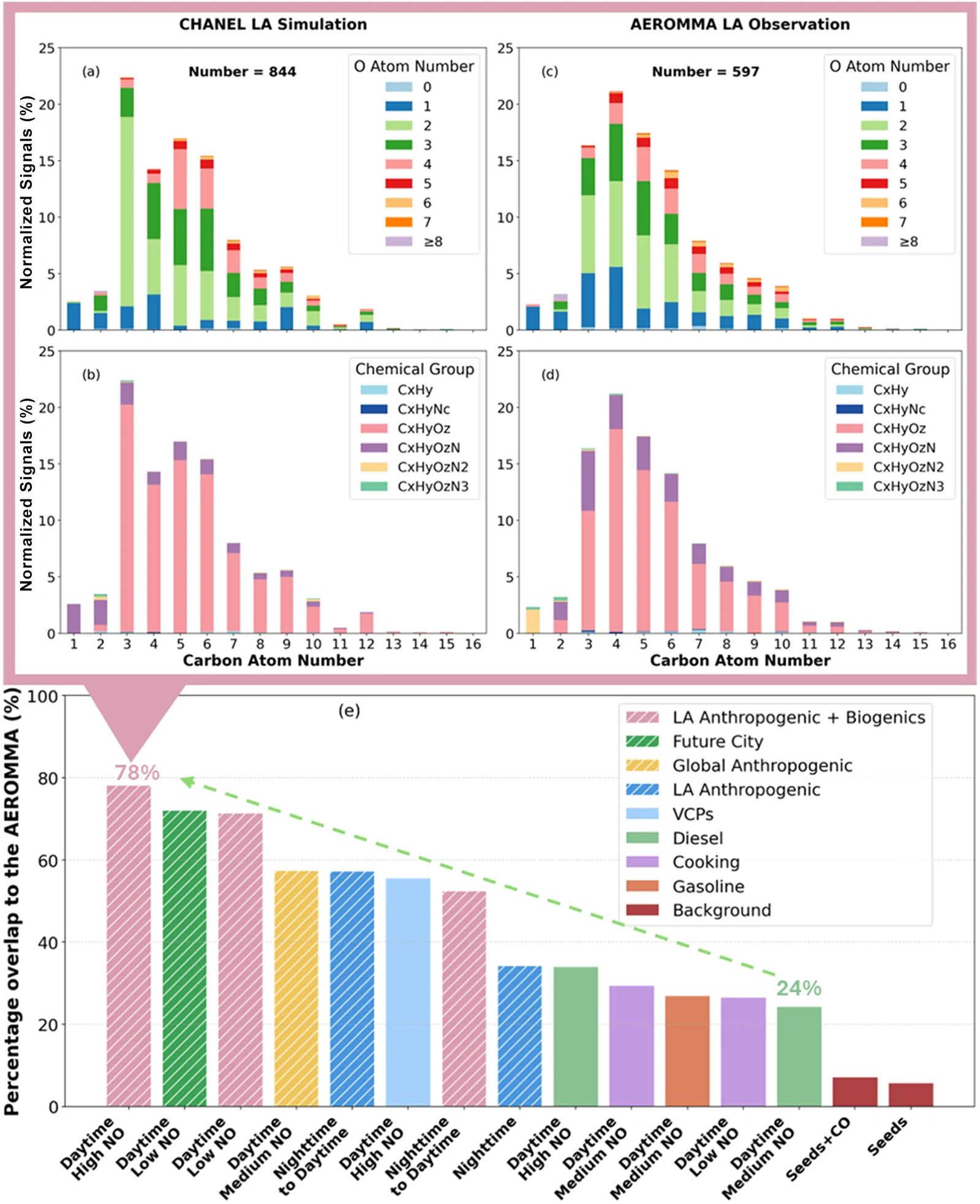
Comparison of VOC diversity between CHANEL chamber experiments and AEROMMA Los Angeles observations. Panels (a–d) show carbon and oxygen atom number distributions and chemical group compositions of compounds measured by the NH_4_^+^ VOCUS instrument in the CHANEL LA simulation experiment (a, b) and AEROMMA LA airborne observations (c, d). Colors denote either oxygen atom number (a, c) or chemical group classification (b, d). Signal intensities of individual product ions were normalized to the total summed signal intensity within each data set. Panel (e) shows the percentage of VOC product ions detected in each CHANEL experiment that overlapped with the AEROMMA LA urban air measurement (*y*-axis). The *x*-axis indicates the oxidation condition (low, medium, or high NO; daytime or nighttime oxidation) of each experiment. Colors denote injection mixtures (Tables S1–S9), and shaded bars indicate multisource experiments.

In contrast, single-source experiments captured substantially smaller fractions of the observed urban chemical space. The diesel-only experiment (Experiment 11) overlapped with just 24% of the LA product ions, consistent with the relatively small contribution of diesel to total fuel consumption in Los Angeles,^49^ though this value may be underestimated due to the limited ability of NH_4_^+^ VOCUS to capture hydrocarbon chemistry. Higher overlaps would be expected in regions with greater diesel usage, such as many European or Indian cities. Among individual anthropogenic sources, VCPs under high-NO condition (Experiment 6) achieved the highest overlap at 55%, highlighting the dominant role of VCPs in shaping secondary urban VOC diversity in cities with reduced vehicle emissions, such as LA.^10^

Anthropogenic mixtures without biogenic emissions (Experiments 19 and 15, representing anthropogenic emissions from LA and more heavily diesel-influenced cities, respectively) reproduced only 57% of LA VOC product ions, demonstrating that anthropogenic emissions alone are insufficient to explain the observed secondary urban VOC diversity. The inclusion of biogenic emissions increased overlap from 57 to 78%, underscoring their critical role even in heavily urbanized environments. A notable exception was the nighttime-to-daytime oxidation experiment combining anthropogenic and biogenic mixtures (Experiment 16), which reproduced only 52% of LA VOC product ions. This experiment was intentionally designed as an extended nighttime exposure, with the SAPHIR chamber roof kept closed for 19.5 h to sustain nighttime oxidation before opening the roof the following morning to initiate daytime chemistry. The prolonged nighttime conditions led to substantial depletion of reactive VOCs and oxidants prior to photochemical processing, therefore limiting the overlap with LA urban air.

#### 3.3.2 Detailed Comparison for the CHANEL LA Simulation

Given its highest overlap, we examine the high-NO CHANEL LA simulation (Experiment 24) in more detail. In total, 844 product ions were measured in SAPHIR, exceeding the 597 observed in Los Angeles. Higher precursor concentrations than in ambient air, together with the operation of SAPHIR as a batch reactor in which oxidation products accumulate rather than being diluted or removed, brought a larger number of secondary products above the instrument detection limit. Across the 465 common VOC product ions, signal intensities showed reasonable agreement between CHANEL and LA urban air (*R* ^2^ = 0.60), with 87% of compounds falling within a factor of 3 of the linear fit and 98% within a factor of 5 (Figure S9).

Carbon number, oxygen number, and chemical group distributions further confirmed the broad compositional similarity between the CHANEL LA simulation and LA urban air (Figure 3a–d), with differences concentrated in a small number of compound classes. CHANEL overrepresented C_12_ products by approximately 0.8%, reflecting the use of dodecane as a surrogate in both VCP and gasoline emissions. C_3_ products were overproduced by approximately 6%, driven primarily by C_3_H_6_O_2_ formed from propylene glycol oxidation, yielding hydroxyacetone and 2-hydroxypropanal^89^ (Figure S10, excluding C_3_H_6_O_2_). These compounds were also present in LA urban air but at lower relative abundance. More details are discussed in Text S2.

C_4_ products exhibited reduced overlap by 7%, with seven product ions missing from the chamber simulation (Text S2 and Table S13). All missing product ions exhibited low signals in LA urban air, each contributing less than 0.1% to the total VOC signals. However, several nitrogen-containing C_4_ product ions (C_4_H_9_NO, C_4_H_11_NO, and C_4_H_7_NO_4_) together accounted for 1.3% of the total signals in LA urban air and deviated by more than a factor of 3 from the overall correlation (Figure S9c), highlighting potential challenges in reproducing certain nitrogen-containing compounds.

This nitrogen-containing product discrepancy is consistent with a broader pattern: the proportion of organic nitrates in LA urban air also exceeded that in the CHANEL LA simulation (Figure 3b,d), most likely reflecting the lower average NO concentration in the SAPHIR chamber (0.2 ppbv, Table S11) compared to LA ambient air (1 ppbv).

More broadly, the 132 product ions detected in LA urban air but absent from the CHANEL LA simulation likely reflect a combination of factors, including organic nitrates less efficiently formed under the lower NO conditions of the chamber, some oxidation products from compounds lumped during fingerprint construction, and differences in geographic and meteorological context between the two data sets. Collectively, these unmatched species accounted for only about 3% of the total ambient VOC signals, indicating that they are minor contributors to the overall VOC burden and are unlikely to substantially affect the representativeness of the fingerprint approach.

#### 3.3.3 Source-Dependent Contributions to Carbon and Oxygen Atom Distributions

Carbon and oxygen atom number distributions provide a compact view of how individual emission sources contribute to the chemical product space observed in LA urban air (Figure S11). Single-source experiments exhibit distinct and incomplete atom distributions relative to LA urban air. Gasoline gives rise predominantly to low-carbon-number products, cooking emissions dominate intermediate carbon classes, and VCPs distinctively contribute substantial C_3_ and C_10_ products, with the latter predominantly originating from monoterpene oxidation.

Despite these source-specific signatures, none of the single-source experiments reproduce the full oxidation product atom distribution observed in LA urban air. The persistent gaps were substantially reduced only when multiple anthropogenic and biogenic sources were combined, demonstrating that urban chemical complexity emerges from the simultaneous oxidation of diverse emission sources rather than from any individual sector alone.

Taken together, these comparisons demonstrate that the multisource CHANEL mixtures reproduce a large fraction of the secondary product diversity observed in LA urban air. While individual source experiments capture specific subsets of this chemical space, only combined mixtures recover the emergent complexity arising from interactions among anthropogenic and biogenic emissions. This establishes a quantitative benchmark for evaluating the representativeness of chamber experiments against real-world urban atmospheres.

## 4 Implications

This study establishes a new experimental approach for chamber studies, capturing key aspects of the oxidation product complexity and radical chemistry of cities using inventory-based fingerprints. CHANEL mixtures approached atmospherically relevant peroxy radical fates and captured 78% of the product ion diversity of Los Angeles air as measured by NH_4_^+^ VOCUS, showing that mixture-based chamber experiments can narrow the gap between controlled laboratory studies and the real atmosphere.

The fingerprint framework enables systematic testing of emission scenarios under controlled but atmospherically relevant conditions. Future cities are expected to experience steep reductions in traffic-related emissions alongside persistent or increasing contributions from VCPs and cooking, as well as stress-induced biogenic emissions. By adjusting the source fingerprints, chamber simulations can explore how such shifts affect radical chemistry, SOA formation, and ozone production. While single-compound experiments remain valuable for developing and refining chemical mechanisms, mixture experiments provide essential complementary insights into how source interactions drive chemical variability under realistic urban conditions — a dimension that cannot be captured by single-precursor studies alone. The same framework can also be extended to other complex emission sources such as biomass burning, industrial plumes, or indoor air, offering a unified and transferable method that advances chamber experiments toward truly representative atmospheric conditions.

Beyond chamber applications, the multisource fingerprint experiments could also be used to evaluate and improve chemical mechanisms in atmospheric models. While models have made considerable progress in representing emissions, substantial uncertainties remain in the chemical mechanisms themselves, particularly in reproducing secondary VOC distributions, NO*_x_* sinks, and SOA formation from complex mixtures. The experimental permutations enabled by the fingerprint approach, combined with the breadth of instrumentation deployed during CHANEL and the near-ambient RO_2_ fates achieved in SAPHIR, create a well-constrained data set for mechanism evaluation. Moreover, because the chemical simplifications applied in the fingerprints mirror the reduced precursor sets implemented in models such as CMAQ, chamber-derived constraints on OH reactivity, volatility distributions, and SOA yields can be directly translated into model parameters, creating a consistent bridge between experimental design and model representation. Preliminary evidence from a companion CHANEL study suggests that mixed-precursor systems produce markedly different SOA outcomes from single-precursor experiments. The presence of multiple reactive species can suppress SOA formation relative to single-compound experiments such as limonene-only oxidation, which supports the case for mixture-based studies in atmospherically relevant SOA quantification.

The CHANEL experiments also highlight important limitations. Fingerprint representativeness is inherently linked to emission inventory quality. For diesel emissions, unresolved compounds, primarily consisting of intermediate-volatility organic compounds (IVOCs), likely lead to SOAP underestimation. Incomplete transfer of low-volatility compounds and the absence of primary carbonaceous aerosol, which can act as an I/SVOC reservoir under dilute chamber conditions, introduce further constraints. Lumping within CRACMM categories also does not resolve differences in NO_*x*_ sensitivity among grouped compounds, which adds an additional source of uncertainty. Although wall losses in the SAPHIR chamber are expected to be minor, sticky or low-volatility compounds may still be affected, potentially limiting the representativeness of secondary production relative to ambient conditions as further highlighted in the supplement. Because SAPHIR operates as a batch reactor, oxidation products accumulate rather than being diluted or deposited, so the product distribution is weighted toward later-generation chemistry relative to ambient air. Additionally, NO levels in the experiments were generally lower than those in ambient air, which could lead to reduced production of organic nitrates. Finally, the comparison with AEROMMA field measurements, though encouraging, was based on a single instrument pair that could not separate isomers, and should therefore be regarded as indicative rather than comprehensive. Because the mass spectrometer itself does not provide structural information, complementary techniques, such as gas chromatography, liquid chromatography, or low-pressure-ion mobility CIMS can be applied in the future studies to resolve structural isomers. Future refinements should also prioritize improved IVOC and SVOC characterization, incorporation of primary aerosol, broader field-chamber instrument comparisons, and closer matching of NO_*x*_ conditions to ambient levels.

Looking ahead, the methodology can be extended across regions, seasons, and climates, as well as to additional source classes not yet represented here, e.g., industrial, asphalt, and biomass burning emissions. Applying inventory-based fingerprints in other chambers or with different oxidant exposures would test the generality of the approach. Since the present validation is based on a single case study (Los Angeles), extending the comparison between chamber results and field campaigns in multiple cities could reveal universal versus region-specific drivers of SOA and ozone. Furthermore, this framework can be extended to investigate source-specific hazardous VOCs and their implications for health risk assessment. Beyond fundamental science, the advances toward reproducing the “breath of cities” in chambers open new opportunities for policy-relevant research, including evaluation of mitigation strategies for health and climate impacts.

In this context, it is worth noting the foundational contribution of earlier mixed-VOC chamber studies that demonstrated the policy relevance of VOC mixture experiments, particularly through maximum incremental reactivity, a metric widely used in regulatory frameworks to quantify ozone formation potential and guide emission controls.^27^ Building on this foundation, the source-resolved emission fingerprints developed here could link emission inventories with chamber chemistry, enabling simultaneous representation of ozone and SOA formation under more atmospherically relevant conditions compared to previous work and providing a more comprehensive framework for evaluating urban air quality mitigation strategies.

In summary, by integrating emission inventories, chamber experiments, and field observations, we establish a scalable framework that brings laboratory studies closer to the chemical reality of urban air. This represents a step change for atmospheric chemistry, enabling chambers not only to dissect mechanisms for individual VOCs, but also extended to complex mixtures under the radical chemistry and molecular diversity that are comparable to the atmosphere.

## Supplementary Material





## Notes

Text, figures, and tables containing additional descriptions of determination of emission inventory profiles for urban sources, the AEROMMA and JULIAC campaign, instrumentation, injection efficiency, VOC and radical turnover, injections and overviews of CHANEL experiments (PDF)
Procedures for generating reduced-complexity chemical fingerprints for major urban anthropogenic emissions (XLSX)

## ☼

Atmospheric Chemistry Observations & Modeling Laboratory, NSF National Center for Atmospheric Research, Boulder, CO, USA

## ◎

Earth, Ocean and Atmospheric Sciences Thrust, The Hong Kong University of Science and Technology (Guangzhou), Guangzhou 511453, China

## ◑

Department of Chemistry, Aarhus University, 8000 Aarhus C, Denmark; and the National Research and Innovation Agency, Indonesia

## Abbreviations

SOA: secondary organic aerosol
VOCs: volatile organic compounds
VCPs: volatile chemical products
O_3_: ozone
NO_*x*_: nitrogen oxide
HO_2_: hydroperoxyl radicals
RO_2_: organic peroxy radicals
NO: nitrogen monoxide
CHANEL: Household Chemicals Amplifying Urban Aerosol Pollution
SAPHIR: Simulation of Atmospheric Photochemistry in a Large Reaction Chamber
JULIAC: Jülich atmospheric chemistry
AEROMMA: Atmospheric Emissions and Reactions Observed from Megacities to Marine Areas
LA: Los Angeles
CRACMM: Community Regional Atmospheric Chemistry Multiphase Mechanism
CMAQ: Community Multiscale Air Quality
HONO: nitrous acid
NO_2_: nitrogen dioxide
CO: carbon monoxide
OVOCs: oxygenated VOCs
D5: D5 siloxane
FIMR: focusing ion–molecule reactor
LIF: laser-induced fluorescence
CMR: chemical modulation reactor
SOAP: secondary organic aerosol formation potential
SOAPu: secondary organic aerosol formation potential units
1-butyl-4-methylNap: 1-butyl-4-methylnaphthalene
1,3,5-TEB: 1,3,5-triethylbenzene
1,2,3-TMB: 1,2,3-trimethylbenzene
1-hexyl-4-MB: 1-hexyl-4-methylbenzene
1,3-dimethyl-5-PB: 1,3-dimethyl-5-propylbenzene
4-ethyltoluene: 4-ethyltoluene or 3-ethyltoluene
MIR: maximum incremental reactivity
