# Supplementary material for: Toward Recreating the Breath of Cities in the Atmospheric Simulation Chamber SAPHIR
Source: Environ Sci Technol. 2026 Oct 14;60(42):29992–30007. doi: 10.1021/acs.est.6c05767 (PMC13718622; doi:10.1021/acs.est.6c05767)
Supplement: Supplementary file 1 [file es6c05767_si_001.pdf]

Supplementary Information for

Toward recreating the breath of cities in the atmospheric simulation chamber SAPHIR

Yizhen Wu[1], Ralf Tillmann[1], Eva Y. Pfannerstill[1,2], Peeyush Khare[1], Andrea Carolina Marcillo Lara[1], Achim Grasse[1], Franz Rohrer[1], Milan Roska[1], Clémence Depp[1], Max Gerrit Adam[1], Sarah Albertin[3,4], Rabbia Asgher[5], Thomas J. Bannan[6], Shawon Barua[5], Kelvin H. Bates[7], David M. Bell[8], Birger Bohn[1], Steven Brown[3], Angela Buchholz[9], Yanfang Chen[8], Changmin Cho[1‡], Hugh Coe[6,10], Matthew M. Coggon[3], Michelle Färber[1], Sana Farhoudian[5], Juliane L. Fry[11], Hendrik Fuchs[1,12], Martin Graus[13], Imad El Haddad[8], Quanfu He[1#], Thorsten Hohaus[1], Siddharth Iyer[5], Vlassis A. Karydis[1], Avinash Kumar[5], Markus Leiminger[13], Lu Liu[8], Emily Matthews[6], Gordon McFiggans[6], Ann M. Middlebrook[3], Markus Müller[13], Aki Nissinen[9], Anke C. Nölscher[1,2], Anna Novelli[1], Farhan R. Nursanto[11], Sebastien Perrier[14], André S. H. Prévôt[8], Iida Pullinen[9], Eka Dian Pusfitasari[9,§], Tobias Reinecke[13], Matti Rissanen[5,15], Matthieu Riva[14], Michael A. Robinson[3,4], Siegfried Schobesberger[9], Chelsea E. Stockwell[3], Jens Top[8], Alexandra P. Tsimpidi[1], Netta Vinkvist[15], Aristeidis Voliotis[6,10], Andreas Wahner[1,2], Yuantao Wang[8], Yuwei Wang[6], Carsten Warneke[3], Robert Wegener[1], Boxing Yang[8], Georgios I. Gkatzelis[1]*

[1] Institute of Climate and Energy Systems: Troposphere (ICE-3), Forschungszentrum Jülich GmbH, Jülich, 52428, Germany

[2] Institute of Geophysics and Meteorology, University of Cologne, 50969 Cologne, Germany

[3]NOAA Chemical Sciences Laboratory, Boulder, CO 80305, USA

[4] Cooperative Institute for Research in Environmental Sciences, University of Colorado, Boulder, CO 80309, USA

[5] Aerosol Physics Laboratory, Physics Unit, Faculty of Engineering and Natural Sciences, Tampere University, 33720 Tampere, Finland

[6] Centre for Atmospheric Sciences, Department of Earth and Environmental Science, The University of Manchester, M13 9PL, Manchester, United Kingdom

[7] Department of Mechanical Engineering, University of Colorado Boulder, Boulder, CO 80309, USA

[8] PSI Center for Energy and Environmental Sciences, Paul Scherrer Institute, 5232 Villigen PSI, Switzerland

[9] Department of Technical Physics, University of Eastern Finland, 70211 Kuopio, Finland

[10] National Centre for Atmospheric Sciences, The University of Manchester, M13 9PL, Manchester, United Kingdom

[11] Department of Environmental Sciences, Wageningen University, 6708 PB Wageningen, Netherlands

[12] Department of Physics, University of Cologne, 50923 Cologne, Germany

[13] IONICON Analytik GmbH, Eduard-Bodem-Gasse 3, 6020 Innsbruck, Austria

[14] Univ. Lyon, Universite Claude Bernard Lyon1, CNRS, IRCELYON, 69626 Villeurbanne, France

[15] Department of Chemistry, University of Helsinki, 00560 Helsinki, Finland

Correspondence to: Georgios I. Gkatzelis (g.gkatzelis@fz-juelich.de)

The PDF file includes:

Text S1: Determination of emission inventory profiles for urban sources

Text S2: AEROMMA comparison to CHANEL

Text S3: JULIAC campaign

Text S4: Further instrumentation

Text S5: Injection protocols and oxidation conditions

Text S6: Injection consistency and efficiency

Text S7: VOC, RO_2, and HO_2 turnover rates

Figure S1: Workflow for constructing condensed emission fingerprints of urban sources.

Figure S2: Cumulative contribution of individual species to OH reactivity and SOAP for inventories of VCPs, gasoline, diesel, and cooking.

Figure S3: Injected chemical fingerprints of major urban emission sources including volatile chemical products (VCPs), diesel, gasoline, and cooking emissions during the CHANEL campaign.

Figure S4: Time series of key parameters during a representative daytime experiment (Experiment 24 in Table S10).

Figure S5: Time series of key parameters during a representative nighttime experiment (Experiment 16 in Table S10).

Figure S6: Optimized inlet for liquid VOC injections.

Figure S7: Radical termination regimes during CHANEL compared with global ambient distributions.

Figure S8: Radical concentrations in CHANEL experiments compared with field observations during the JULIAC campaign.

Figure S9: Comparison of VOC signals between CHANEL and AEROMMA Los Angeles measurements.

Figure S10: Comparison of VOC composition between CHANEL and AEROMMA excluding $C_3H_6O_2$.

Figure S11: Carbon atom number distributions of VOCs measured by NH_4^+ VOCUS in CHANEL chamber experiments and of species observed over Los Angeles during AEROMMA that were absent from the corresponding CHANEL experiment, coloured by oxygen atom number.

Figure S12: Comparison of observed and expected OH reactivity after VOC injections.

Figure S13: Comparison between MCM modelled and measured VOCs concentrations by GC–MS/FID in all experiments during the CHANEL campaign.

Figure S14: Comparison between MCM modelled concentrations and measured signals by NH_4^+ VOCUS in all experiments during the CHANEL campaign.

Figure S15: Comparison between MCM modelled concentrations and measured concentrations by $H3O^+$ FUSION in the gasoline experiment during the CHANEL campaign.

Figure S16: Comparison of measured and expected concentrations of (a) heptanal (C7H14O) and (b) propylene glycol ($C_3H_8O_2$) during CHANEL experiments.

Table S1: Detailed composition of the volatile chemical products (VCP) fingerprint and injection.

Table S2: Detailed composition of the gasoline fingerprint and injection.

Table S3: Detailed composition of the diesel fingerprint and injection.

Table S4: Detailed composition of the cooking fingerprint and injection.

Table S5: Detailed composition of the biogenic injection.

Table S6: Detailed composition of experiment simulating LA Anthropogenic in the CHANEL campaign.

Table S7: Detailed composition of experiment simulating LA Anthropogenic+Biogenic in the CHANEL campaign.

Table S8: Detailed composition of experiment simulating Global Anthropogenic in the CHANEL campaign.

Table S9: Detailed composition of experiment simulating Future City in the CHANEL campaign.

Table S10: Summary of experiments performed during the SAPHIR CHANEL campaign.

Table S11: Average NO, NO_2, NO_x, and O_3 concentration, temperature (T) and relative humidity (RH) of each experiment during the CHANEL campaign.

Table S12: Average OH, HO_2, and RO_2 concentration of each experiment, and total OH reactivity measured at the injection mask during the CHANEL campaign.

Table S13: Signals of C_4 VOCs that were observed over LA during the AEROMMA campaign but missing in the CHANEL LA simulation (experiment 24).

Other Supplementary Material for this manuscript includes the following:

Spreadsheet (.csv)

S1. Determination of emission inventory profiles for urban sources

Urban VOC emissions encompass thousands of reactive compounds from diverse sources, complicating the isolation of source-specific oxidation pathways in controlled chamber experiments. To address this, we reduced the chemical complexity of each source while retaining the dominant precursors that define its key chemical characteristics. This four-step approach preserves the overall reactivity and SOA formation potential of the full emission profile, while keeping the system sufficiently tractable for mechanistic investigation.

First, VOCs in each of the four sources were ranked based on their fractional contributions to two key metrics of pollutant formation in the GRA^{2}PES emission inventory: [1] (i) fractional OH reactivity (eq. 1),[2–8] which weights each VOC by its rate constant relative to the total source reactivity and is closely linked to ozone formation, and (ii) fractional SOA formation potential (SOAP) (eq. 2),[9] which weights each VOC by its SOA yield relative to the total source formation potential. Compounds were retained in the fingerprint in descending order of contribution until 80% of each metric was captured, after which lower-contributing compounds were excluded. Notably, inclusion was determined on a per-metric basis; therefore, compounds falling within the top 80% of either fractional OH reactivity or SOAP were retained in the fingerprint, regardless of their rank in the other metric. The 80% threshold was chosen as a practical balance between chemical representativeness and experimental tractability, ensuring that the dominant reactive species are retained while keeping the number of injected compounds manageable, as shown in Fig.S2.

Second, for compounds reported in the inventory only as chemical classes (e.g., branched C_{12} alkanes in VCPs) or with undefined structures (e.g., $C_8H_{14}O_2$ in cooking emissions), surrogate species were selected either following established methodologies from the literature[1] or by identifying compounds with identical molecular formulas that had been previously reported in field or laboratory measurements of the same source type.[10,11] In both cases, the OH rate constants and SOA formation potential units of the selected surrogates were verified against those of the original inventory entries, and surrogates were only accepted where these properties were comparable, minimizing the introduction of additional uncertainty into the fingerprint's reactivity and SOA formation potential.

Third, compounds were assigned to chemical classes defined by CRACMM, a state-of-the-art reduced mechanism that links gas- and particle-phase chemistry and serves as the basis for widely-used chemical transport models such as the Community Multiscale Air Quality model (CMAQ).[10] Reduced mechanisms such as CRACMM are designed to capture the dominant chemical pathways that govern ozone and SOA formation while keeping the system computationally tractable — properties that directly align with the goals of the fingerprinting approach. Within each CRACMM class, compounds with similar OH rate constants and SOA formation potential units were lumped together and represented by the most abundant member.

Finally, compounds commercially unavailable during the CHANEL campaign were replaced with structurally similar surrogates, verified to have comparable OH rate constants and SOA formation potential units, with any residual discrepancies reflected in the fingerprint uncertainty assessment discussed below.

A characteristic example that follows the above steps is the VCP inventory inclusion of "branched C_{12} alkanes," which contribute substantially to the total OH reactivity. These species were first represented by the surrogate compound 2-methylundecane. During the CRACMM classification, 2-methylundecane and n-undecane fell into the same chemical group (HC10), and because 2-methylundecane had the higher emission contribution, it served as the representative compound for both. For the CHANEL campaign, limited compound availability required substituting 2-methylundecane with n-dodecane, which has comparable reactivity and SOA formation potential.

S2. AEROMMA comparison to CHANEL

The 2023 AEROMMA (Atmospheric Emissions and Reactions Observed from Megacities to Marine Areas) campaign was conducted aboard the NASA DC-8 aircraft, with a comprehensive suite of meteorological and chemical instruments deployed to investigate emissions and their chemical evolution across the United States and Canada.[12]

The NOAA VOCUS long time-of-flight chemical ionization mass spectrometer (Tofwerk AG, Switzerland) operated in NH_4^+ mode at a time resolution of 10 Hz, continuously sampling ambient air through a heated inlet mounted on the aircraft wing. Background measurements were conducted regularly during flights, and 93 calibrations were performed before, during, and after the campaign using gravimetric gas standards, a custom-built liquid calibration system, and an Aerodyne Liquid Calibration Unit. Sensitivities were stable within 15% for most compounds (30% for low-sensitivity species such as isoprene and acetaldehyde), and calibration factors from pre- and post-campaign laboratory calibrations were normalized to acetone to align with in-flight values. During the flights, sensitivities were (1220 $\pm$ 30) cps/ppb for acetone, (800 $\pm$ 20) cps/ppb for monoterpenes, and (5740 $\pm$ 680) cps/ppb for D5 siloxane (D5), which was similar with the sensitivity during the CHANEL campaign. Detailed calibration results and comparison to CHANEL campaign were shown by Roska et al.[13] Instrument response remained stable within 20% during urban flight periods. Data processing included mass calibration, peak fitting, and molecular formula assignment with Tofware (v4.0.0), followed by background subtraction, cleaning, and averaging in Igor Pro. Same peak list was used for the CHANEL campaign for clearer comparison between the chamber experiment and field measurement.

To assess how well the CHANEL experiments reproduced atmospheric radical lifetime distributions, radical chemistry regimes measured in CHANEL were compared with observations over large North

American urban areas from the AEROMMA campaign, following the framework described in detail by Robinson et al.[14]

Furthermore, we compared VOCs observed in the CHANEL experiments to those measured by NH_4^+ VOCUS over Los Angeles. We excluded injection tracers — precursor VOCs directly injected into the CHANEL chamber, identified based on the known injection fingerprint composition (Tables S1–S9) — from both datasets, as well as compounds showing high ambient background signals during AEROMMA, i.e., acetone, ethanol, and acetonitrile, and compared chamber endpoints (final two hours of each experiment, representing predominantly secondary oxidation products) with LA urban boundary air, retaining only measurements where NO_2 levels exceeded 1 ppbv to exclude suburban air. Agreement was evaluated using overlap fractions, regression (R^2), and factor-of-three/five metrics. The following subsections provide additional compound-specific details and inventory considerations to complement the main text discussion.

(1) Comparison of the CHANEL LA simulation (experiment 24; Table S10) with LA urban air

Specific discrepancies (Fig. S9)

- **$C_5H_{10}O$**: This product ion, likely pentanal, was included in the cooking emission inventory[1,15–19] with a relatively high emission fraction (3.8% wt) but was lumped with propanal (7.6% wt) during the fingerprint generation process, as the two compounds share similar OH rate constants and SOA formation potential units. As a result, pentanal was underestimated in the CHANEL LA simulation compared to field observations.
- **C_3H_9NO**: It was strongly underrepresented in CHANEL relative to LA measurements. This discrepancy may result from the lower average NO concentration in the SAPHIR chamber. But this product ion may also represent a fragment of 2-amino-2-methyl-1-propanol ($C_4H_{11}NO$, 0.1% wt) reported in the VCP inventory.[1,15–19]

Both $C_5H_{10}O$ and C_3H_9NO showed substantially higher signals in LA compared to other U.S. cities during AEROMMA, suggesting region-specific sources or emission factors not captured in the inventories.

C_3 products (Fig. 3)

- CHANEL overrepresented $C_3H_6O_2$, which arises predominantly from VCP injections. In the VCP fingerprint list, propylene glycol ($C_3H_8O_2$) was used as a surrogate. $C_3H_8O_2$ can react with OH radicals to produce hydroxyacetone and 2-hydroxypropanal, both of which share the formula $C_3H_6O_2$. $C_3H_6O_2$ was abundant in both CHANEL and AEROMMA LA observations and fell within a factor of three of the regression line (Fig. S9a).

C_4 products (Fig. 3)

- Seven C_4 VOCs were detected only in LA urban air and were absent from CHANEL LA (Table S13). All exhibited very low signals (< 0.1% of total VOC signals).

- Several common C_4 products, including C_4H_9NO, $C_4H_{11}NO$, and $C_4H_7NO_4$, were underproduced in CHANEL relative to LA. In particular, $C_4H_{11}NO$, reported as 2-amino-2-methyl-1-propanol in the VCP emission inventory,[1,15,16] was underrepresented by approximately 25-fold in CHANEL; while $C_4H_7NO_4$ might be produced by oxidation of butanal, as butanal is reported in the cooking emission inventory.[1,16,17] These results indicate that nitrogen-containing VOCs of intermediate volatility remain challenging to simulate with the chosen surrogate-based fingerprints of this study.

C_{12} products (Fig. 3)

- CHANEL overrepresented C_{12} products due to injections of dodecane ($C_{12}H_{26}$), which was used as a surrogate for both VCPs and gasoline emissions. This design choice increased the relative contribution of C_{12}-containing products compared to AEROMMA LA observations.

(2) Comparison between Source-specific CHANEL experiments to LA urban air (Fig. S11)

- Single-source experiments reproduced distinct atom distributions but missed large fractions of urban air complexity. Gasoline contributed strongly to C_1–C_2 species, cooking to C_5–C_6 species, and traffic fuels to C_{12} species. VCPs dominated the C_3 class and reduced the absence of monoterpenes (C_{10}) that was evident in other single-source experiments. However, none of the single-source experiments matched the full diversity of LA urban air.
- VCPs-only oxidation experiments showed the best overlap among single sources (55% of LA VOCs) and even exceeded one of the nighttime mixture experiments (Experiment 16). Diesel-only experiments performed worst (24% overlap). These comparisons highlight the dominant role of VCPs as an emerging driver of urban VOC diversity.

S3. JULIAC campaign

The Jülich Atmospheric Chemistry (JULIAC) campaign was conducted at Forschungszentrum Jülich, Germany, in four month-long intensive phases covering all seasons of 2019. In this study, we compared the radical regimes that we measured in the CHANEL experiments to the summertime measurements during the JULIAC campaign, to validate the atmospherically realistic radical regimes that were achieved during the CHANEL campaign.

During the JULIAC campaign, ambient air influenced by biogenic emissions from surrounding forests and anthropogenic inputs from nearby urban areas was sampled through a 50 m inlet and subsequently introduced into the SAPHIR chamber. OH, HO_2, and RO_2 radicals were measured with the FZJ laser-induced fluorescence (LIF) instrument, the total OH reactivity (k_{OH}) was measured in ambient air by a laser-flash photolysis LIF instrument, while NO was measured by chemiluminescence (Eco Physics, TR780, NO_2 conversion by a custom-built photolytic converter). Further details can be found in previous study.[20]

S4. Further instrumentation

The CHANEL campaign employed an extensive suite of instruments to characterize gas- and particle-phase chemical composition, radical concentrations, and physical aerosol properties. In addition to the instruments described in the main text ($NH4^+$ VOCUS, ROxLIF, and Ecophysics CLD-780 TR), the campaign deployed a broad range of complementary instruments including: NO_y and NO_x analysers, an Aerosol Mass Spectrometer (AMS), Scanning Mobility Particle Sizers (SMPS), an Orbitrap mass spectrometer, a FUSION PTR-TOF operated in both NH_4^+ and H_3O^+ modes, a CHARON inlet coupled to PTR-MS, a total OH reactivity instrument (kOH-LIF), a Multi-Reagent Chemical Ionisation Mass Spectrometer (MR-CIMS) operated with iodide, acetone, bromide, and benzene reagent ions, a Filter Inlet for Gases and AEROsols (FIGAERO) coupled to iodide chemical ionization, an Extractive Electrospray Ionisation mass spectrometer (EESI), an Electrical Low Pressure Impactor (ELPI), an amine high-resolution time-of-flight instrument (amine-HTOF), photolysis frequency radiometers, HONO and Picarro trace gas analysers, and a GC-ACTRIS system for non-methane hydrocarbons. Results from select instruments are presented in this Supplementary Information, with further analyses to be reported in companion publications from the CHANEL campaign.

S4.1 VOCUS

The targeted reagent ions, $NH_4^+ \cdot H_2O$, were generated in the ion source by mixing two gas streams, a 21 sccm water vapor flow sourced from the headspace of ultra-pure water, and a 0.5 sccm ammonia-containing flow from the headspace of a 1 vol% ammonium hydroxide solution. Water molecules were initially ionized to form H_3O+, which subsequently reacted with NH_3 to produce NH_4^+ and further clustered with water to yield the desired $NH_4^+ \cdot H_2O$ reagent ions. The Focusing Ion–Molecule Reactor (FIMR) was maintained at 60 °C, 3 mbar, and a voltage gradient of 510 V.

VOC sampling was conducted through a 2-meter-long, heated 1/4" outer diameter Teflon tube, with a total flow rate of approximately 2 L min^{-1}. Approximately 100 sccm of this air stream was directed into the FIMR via a 25 mm-long PEEK capillary with a 0.18 mm inner diameter.

Instrument background measurements were conducted for 5 minutes every hour, and daily calibrations were conducted using a certified 20-compound VOC gas mixture (NPL Management Ltd., UK). Instrument sensitivity remained stable throughout the campaign, with (1700 ± 230) cps/ppb for acetone, (220 ± 30) cps/ppb for 3-carene, and (4770 ± 610) cps/ppb for D5 siloxane (D5). The instrument mass resolution was around 9950 Th/Th. Mass calibration, peak fitting, and compound identification were performed using Tofware (v4.0.0, Tofwerk AG, Switzerland), and background subtraction, time averaging, and data cleaning were carried out using custom Igor Pro scripts.

In this study, wall losses of VOCs were not taken into consideration, since the SAPHIR chamber has a low surface-to-volume ratio of around 1 m^{-1}, which minimizes wall effects. However, a previous study

has shown that in the SAPHIR chamber, Master Chemical Mechanism (MCM) predictions that did not account for radical wall losses still showed good agreement with measured OH, HO_2, and RO_2 concentrations.[21]

Ionization product distributions of common calibrants showed similar patterns in the CHANEL and AEROMMA campaigns, with NH_4^+ dominating the ionization pathways, as NH_4^+·acetone and NH_4^+· D5 accounted for more than 90% of the acetone and D5 product ions, respectively.

Besides VOCUS, an online thermal desorption gas chromatography system coupled to dual flame ionization and mass spectrometric detection (GC-TD-FID/MS; Markes TT24-7xr with Kori-xr units, Agilent 8890 GC, Agilent 5977B MS) was used for non-methane hydrocarbon (NMHC, C2–C12) measurements, and a FUSION PTR-TOF (FUSION PTR-TOF 10k, IONICON Analytik, Austria) for aromatic and oxygenated compounds.

S4.2 GC-MS/FID

Non-methane hydrocarbons (NMHCs; C_2–C_{12}) were measured with an online thermal desorption gas chromatography system coupled to dual flame ionization and mass spectrometric detection (GC-TD-FID/MS; Markes TT24-7xr with Kori-xr units, Agilent 8890 GC, Agilent 5977B MS). The TD unit used alternating twin traps for continuous sampling (44 min at 24 mL min^{-1}) followed by desorption at 300 °C with helium (6.0 grade) as carrier gas. Air from the SAPHIR chamber was drawn through a 7.2 m, 1/4″ SilcoNert-coated stainless steel manifold (50 °C). The sample flow was diluted with synthetic air (grade 6.0) by a factor of 1.75, with the addition of 8 mL min^{-1} NO for O_3 titration and 2.6 mL min^{-1} of an internal standard. The diluted sample was then transferred to the instrument through a 1 m long, 1/16″ SilcoNert-coated line. During the CHANEL campaign, calibrations were performed over 20 runs using three standards—an NPL 29-component NMHC mix, an NPL 5-component monoterpene mix, and a self-prepared multi-compound standard. Data were acquired with Agilent ChemStation/OpenLab CDS, with FID peaks semi-automatically integrated and MS analysis limited to target compounds, and concentrations were calculated using Python scripts with calibration curves.

S4.3 FUSION PTR-TOF 10k

The FUSION PTR-TOF (FUSION PTR-TOF 10k, IONICON Analytik, Austria) is a high-resolution proton-transfer-reaction time-of-flight mass spectrometer equipped with a hollow cathode discharge ion source capable of generating multiple reagent ions.[22] In the CHANEL experiments, FUSION PTR-TOF was operated at a time resolution of 1 Hz and measurements were performed using both H_3O^+ and NH_4^+ as reagent ions, but only data in H_3O^+ mode were used in this Supplementary document. A continuous sample flow of 500 sccm was drawn from the SAPHIR chamber via a 1.2-meter thermally insulated perfluoroalkoxy (PFA) line. The sampled air passed through the instrument's inlet system, which included a 1.5-meter long, 0.040" ID Sulfinert-coated stainless-steel tube (Restek Corp., Bellefonte, PA, USA)

heated to 90 °C to minimize wall interactions. Daily calibrations were performed using a certified gas standard containing a representative set of atmospherically relevant compounds. Instrument sensitivities, detection limits, and transmission efficiencies across different mass-to-charge ratios were evaluated using PTR-TOF Viewer (v3.4.5). Mass calibration, peak fitting, and compound identification were performed with the IONICON Data Analyzer (IDA, v2.2.2.1), and custom Igor Pro scripts were used for background subtraction, data averaging, and cleaning.

S4.4 Measurements of radicals

For the radical measurements, chamber air was drawn through a conical nozzle into a low-pressure cell (~4 hPa), where OH radicals were excited by short laser pulses (308 nm, 8.5 kHz), and their subsequent fluorescence was detected by a gated microchannel plate detector. In the chemical modulation reactor, propane (Air Liquide, >99.95% purity, (5.0±0.1)% in N_2) was periodically injected to scavenge ambient OH. For the ROx measurement, sampled air entered a converter (~25 hPa), where RO_2 radicals were sequentially converted to HO_2 and then to OH in reactions with added NO. With excess CO, OH formed from HO_2 + NO was reconverted to HO_2 in the converter, while HO_2 was then completely transformed to OH with pure NO in another low-pressure detection cell downstream of the converter. Potential interferences from certain RO2 species, which rapidly generate HO_2 through RO_2 + NO, were negligible as the HO2 detection cell was operated at low NO levels in this study.

In the HO_X and RO_X detection cells, where NO addition is required, background signals equivalent to $(8.3±0.5)×10^7$ cm^{-3} and $(5.0±2.6)×10^7$ cm^{-3}, respectively, were observed. These were determined during calibrations with clean, humidified synthetic air. The exact cause of this NO-related background remains unresolved but it has been attributed to impurity present in the cylinders used for the NO injections.[23] Such impurities are minimized by using Ascarite (sodium hydroxide-coated silica) as a scrubber specifically for HONO prior to the injections and are expected to be rather constant during CHANEL as the same NO cylinders were used for all channels throughout the entire campaign.

S4.5 Total OH reactivity measurement

Total OH reactivity (k_{OH}), the inverse of the OH chemical lifetime, was measured with a laser-flash photolysis LIF instrument.[24] OH was generated by 266 nm flash photolysis of ozone in humid air within a flow tube above the OH fluorescence cell. The pseudo-first-order OH decay due to reactions with atmospheric reactants was measured directly. Chamber air was sampled through a stainless-steel inlet (10 mm I.D., 6 m length) coated with SilcoNert® and operated at 19 sL min^{-1}, yielding a residence time of ~1.5 s and minimizing losses of reactive species.

S4.6 Measurements of NO and NO_2

Calibration of the Ecophysics CLD-780 TR was conducted using a gas-phase titration (GPT) system. In zero mode, the instrument sampled high-purity zero air (6.0) to establish the instrument baseline. In NO mode, a primary NO standard (2.048 ± 0.016 µmol mol^{-1}) was diluted with zero air, while in NO_2 mode, excess ozone was added to the NO standard to quantitatively generate NO_2.

S4.7 Aerosol Mass Spectrometer (AMS)

Non-refractory submicron aerosols were measured with a high-resolution time-of-flight aerosol mass spectrometer (HR-ToF-AMS) operated in V-mode at 1-minute resolution (detection limit ~3 ng m^{-3}, ~20% uncertainty).[25] Aerosols were sampled through a 5-meter stainless steel inlet shared with an SMPS, and the instrument was calibrated weekly with size-selected 350 nm dry ammonium nitrate particles to determine the ionization efficiency (IE). In addition, the relative ionization efficiency (RIE) for sulfate was assessed using ammonium sulfate particles. Data were analyzed using the PIKA 1.24 software toolkit with high-resolution peak fitting, and elemental ratios (O:C, H:C) were corrected via the Improved-Ambient method.[26]

S4.8 O_3 measurement

O_3 was measured by the 2B Technologies Model G211 Ozone Monitor, using the UV absorption method, a standard technique based on the Beer-Lambert law. Sample air was drawn through the instrument in two alternating paths: one which includes an ozone scrubber to remove ozone, and another that passes air directly to the measurement cell. By comparing the UV light intensity transmitted through these two paths, measured with a photodiode detector, ozone concentration was calculated based on differential absorption. Data averaged to 60s bins result in a detection limit (3s) of 1.1 ppb.

S4.9 HONO measurement

HONO was measured by the Airyx ICAD HONO/NO2 200L S/N 1062, using Iterative Cavity-Enhanced Differential Optical Absorption Spectroscopy (ICAD), a CE-DOAS–based technique.[27] Data were averaged to 120 s intervals, with a 3σ detection limit of 80 ppt.

S4.10 Photolysis frequencies measurement

Total and diffuse solar spectral actinic flux densities in the wavelength range 280 – 660 nm are measured outside of SAPHIR using a sun-tracker and two CCD array spectroradiometers with a spectral resolution of about 2 nm and a temporal resolution of 10 s. The instruments are calibrated with lamps traceable to national standards and corrected with respect to their angular reception characteristics.[28,29] Construction conditioned, the fraction of radiation that enters the chamber depends on solar zenith and solar azimuth angels. A model was developed to account for these effects and to predict chamber-mean

spectral actinic flux densities for clear-sky and cloudy conditions.[30] From these spectra photolysis frequencies were calculated. The validity of the approach is tested regularly by actinometric experiments.[31]

S5. Injection protocols and oxidation conditions

The injection protocols of the experiments are as follows:

(1) Humidification: SAPHIR was initially humidified to approximately 60% RH at around 20 °C (20% for Experiment 2; Table S10). During daytime experiments, RH gradually decreased to 20% due to increases in chamber temperatures to 35 °C when the SAPHIR roof was open (Fig. S4).

(2) Seed aerosol injection: approximately 10 µg/m^3 of ammonium sulfate seed particles with geometric mean diameters of about 100 nm were added to the chamber (except Experiment 17).

(3) Chemical mixtures injection: liquid VOCs were introduced through a custom-designed SilcoNert-coated stainless steel line (~31 cm) maintained at 60 °C (Fig. S6). The line was connected to a T-valve, where injected liquids were rapidly vaporized and mixed with a high-flow stream of heated synthetic air before entering the chamber. High-volatility gases were introduced directly into the chamber from pressurized gas cylinders using calibrated mass flow controllers.

(4) Mixing: chamber fans homogenized injected compounds within a few minutes. Injection efficiencies varied across compound classes, particularly for lower-volatility and sticky compounds such as propylene glycol and are characterized in detail in Text S6.

Different oxidation conditions were simulated across experiments by adjusting NO, NO_2, O_3, or carbon monoxide (CO) levels and by opening or closing the SAPHIR chamber roof:

- **High-NO**: NO (7.5 ppbv) was injected to supplement the NO formed by HONO photolysis from the SAPHIR chamber surface. In the LA simulation experiment (experiment 24), 8 ppbv of NO_2 was injected instead of NO. Average NO mixing ratios of the high-NO CHANEL experiments ranged from 0.23 to 1.05 ppbv (Table S11).
- **Low-NO**: 50-100 ppbv of O_3 was injected to titrate NO formed in the chamber. In low-NO experiments, NO mixing ratios averaged between 0.07 and 0.19 ppbv (Table S11).
- **Medium-NO**: Neither NO/NO_2 nor O_3 was injected; NO concentration accumulated via photolysis of HONO emitted from the Teflon surface of SAPHIR. For the medium-NO experiments, the average NO mixing ratio was 0.15 to 0.31 ppbv (Table S11).
- **Daytime oxidation**: The chamber roof was opened, allowing sunlight to generate OH radicals through HONO photolysis.
- **Nighttime oxidation**: O_3 (10-40 ppbv) and NO_2 (17-40 ppbv) were injected with the chamber roof closed to form NO_3 radicals.
- **Pure ozonolysis**: 75 ppbv of O_3 was injected with the roof closed.

- **OH suppression**: CO (2-60 ppmv) was added in selected experiments to scavenge OH radicals.

S6. Injection consistency and efficiency

Injection consistency during the CHANEL campaign was evaluated using a combination of complementary diagnostics probing different levels of chemical detail. First, bulk consistency was assessed using total OH reactivity, which provides an integrated measure of the combined reactivity of all injected VOCs. Second, compound-resolved consistency was evaluated using gas-phase measurements from GC–MS/FID, H_3O^+ FUSION, and NH_4^+ VOCUS, enabling inspection of individual injected species. Because direct GC overlap with injection periods was limited in duration and available for only a subset of experiments, these measurements were further interpreted using zero-dimensional simulations based on the Master Chemical Mechanism (MCM). This approach enabled a more statistically robust evaluation of injection consistency by comparing modelled concentration time series with observed decay and instrument response. Together, these diagnostics provide a consistent framework for verifying that injected mixtures matched experimental targets within known and quantifiable uncertainties.

S6.1 Evaluation using total OH reactivity

As a first-order consistency check, total OH reactivity measured at the injection mask was compared with values calculated from target injected VOC mixtures (Fig. S12). This metric evaluates whether the overall oxidative capacity introduced into the chamber matched expectations, independent of compound-specific detection limitations.

Experiments without biogenic emissions generally showed good agreement between measured and calculated OH reactivity, clustering near the 1:1 line. In contrast, experiments including biogenic mixtures systematically exhibited lower measured OH reactivity. This offset was driven primarily by under-injection of isoprene, which was introduced as part of a liquid biogenic mixture and partially evaporated during solution preparation and injection. Independent GC–MS/FID measurements confirmed that isoprene concentrations were approximately a factor of three lower than targeted. Based on this verification, the expected isoprene contribution was reduced by a factor of three in subsequent consistency evaluations.

After accounting for this correction, nearly all experiments collapse onto the 1:1 line, with only three experiments (Experiments 13, 21, and 23) remaining as outliers. This demonstrates that, aside from known compound-specific limitations, the injected mixtures achieved the intended total reactivity across the CHANEL campaign.

S6.2 Compound-resolved consistency using MCM-assisted comparisons

GC–MS/FID measurements played a central diagnostic role in evaluating injection consistency during CHANEL. In particular, they independently confirmed the systematic under-injection of isoprene by approximately a factor of three when introduced as part of the liquid biogenic mixture, consistent with the total OH reactivity analysis. This verification established the GC as a critical reference for identifying genuine injection limitations. Beyond this case, the GC data also revealed compound-dependent and time-dependent sensitivity behaviour that needed to be considered when interpreting compound-resolved comparisons with MCM predictions.

For liquid-injected monoterpenes, examination of individual species provided more insight than consideration of their summed signal. As shown in Fig. S13, α-Pinene showed excellent agreement between GC observations and MCM predictions across the full campaign, with slopes close to unity and very high correlation ($R^2 = 0.97$), indicating stable injection, transfer, and detection. β-Pinene, in contrast, was systematically underpredicted relative to MCM expectations but exhibited strong internal consistency ($R^2 = 0.92$). This behavior suggests a compound-specific limitation rather than variable injection efficiency. One plausible contributor is reduced effective transfer or incomplete incorporation of β-pinene during preparation of the liquid terpene mixtures, which could lead to a consistent underrepresentation across experiments.

Limonene displayed a distinct temporal pattern: early experiments showed good agreement with MCM predictions, whereas later experiments consistently fell below the one-to-one line. Because injection solutions remained unchanged throughout the campaign and α-pinene did not show a similar trend, this behavior was most consistent with a shift in GC sensitivity for limonene over time rather than a change in injected composition. As limonene contributed the largest fraction to the total monoterpene signal, this sensitivity shift disproportionately affected comparisons based on summed monoterpenes. Accordingly, while the summed monoterpene signal showed broadly reasonable agreement overall, interpretation based on individual monoterpenes is more diagnostically meaningful.

Independent comparisons using NH_4^+ VOCUS measurements further refined this interpretation (Fig. S14). When considering the summed monoterpene signal, ammonium-based product ion intensities showed good agreement with MCM predictions for experiments that included biogenic mixtures, indicating that the overall monoterpene burden introduced into the chamber was well represented under these conditions. In contrast, for experiments without biogenic emissions—such as single-source injections of VCPs or cooking emissions that nevertheless contain monoterpene tracers— NH_4^+ VOCUS often reported lower signals than expected from MCM. This systematic underprediction suggests that part of the discrepancy reflects real limitations in effective monoterpene delivery, potentially linked to incomplete incorporation or transfer losses during preparation of liquid injection solutions, rather than solely analytical bias. This behaviour is consistent with GC indications that certain terpene components,

particularly β-pinene, may not have been introduced at their nominal target levels. At the same time, limonene exhibited experiment-to-experiment shifts that were not mirrored in the ammonium measurements and were therefore more consistent with GC sensitivity changes over the course of the campaign. Taken together, these observations indicate that NH_4^+ VOCUS captured the relative variability of injected monoterpenes reliably, while highlighting compound- and mixture-dependent limitations in effective delivery rather than a uniform instrumental artifact.

Aromatic compounds showed analogous behavior. Benzene agreed well with MCM predictions in most experiments (R^2 = 0.91 for GC–MCM, Fig. S14; $R^2 \approx 1$ for FUSION–MCM, Fig. S15), though both GC and FUSION measurements showed a consistent ~50% lower response in the gasoline experiment, indicating reduced effective injection. Toluene showed generally good agreement at lower concentrations but deviated at higher concentrations, yielding a lower overall GC–MCM correlation across the campaign (R^2 = 0.68), with a similar offset in the gasoline experiment; FUSION–MCM agreement in the gasoline experiment was substantially higher ($R^2 \approx 1$). Xylenes showed a coherent but systematically lower response relative to MCM expectations across most experiments, with early VCP experiments displaying near 1:1 agreement and later experiments falling below. The consistently lower aromatic concentrations in the gasoline experiment suggest potential underestimation of aromatic chemistry under those conditions.

Compounds introduced via pressurized gas cylinders exhibited different behavior. Propene showed a strong GC–MCM correlation (R^2 = 0.85) but with a systematic offset, with MCM-predicted concentrations exceeding GC observations by ~45% across most experiments. The high cross-experiment consistency suggests stable relative injection behavior, but either a lower-than-expected effective injected concentration or a GC sensitivity bias for propene. Diesel-only experiments showed slightly larger underprediction, with no clear temporal trend.

For 1,3-butadiene, measurable concentrations were observed almost exclusively in gasoline-related experiments, likely reflecting either incomplete delivery to the chamber or limited GC sensitivity under the applied conditions. Similarly, butene showed reasonable agreement in gasoline experiments but systematic underestimation in others despite use of the same gas cylinder, suggesting compound-dependent losses or analytical limitations rather than experiment-specific injection failures. Across all gas-cylinder species, no campaign-wide shift in GC sensitivity was evident, and deviations appeared compound-specific and sporadic.

Compound-dependent transfer behavior during liquid injections was further assessed using VOCUS measurements (Fig. S16). Aldehydes such as heptanal showed good agreement with expected concentrations, indicating efficient vaporization and delivery. In contrast, viscous compounds such as propylene glycol showed larger variability, consistent with enhanced wall interactions and losses during injection — a general limitation of chamber experiments even in large-volume facilities such as SAPHIR.

Critically, these compound-resolved comparisons show that injection consistency during CHANEL was generally robust, while also revealing clear compound-dependent limitations arising from a combination of analytical sensitivity shifts, transfer efficiency constraints, and incomplete incorporation during preparation of liquid mixtures. These effects were internally consistent across experiments and did not compromise relative comparisons or interpretation of chemical evolution. Rather, they define the bounds of uncertainty for specific compounds and highlight the value of combining bulk OH reactivity, GC diagnostics, and mass-spectrometric comparisons to obtain a transparent and internally consistent assessment of injection performance.

S7. VOC, RO_2, and HO_2 turnover rates

To characterize the radical cycling initiated by VOC oxidation, we calculated turnover rates of VOCs, RO_2, and HO_2 as:

$$VOC\ turnover = [OH] \times k_{OH} \quad (5)$$

$$RO_2\ turnover = [RO_2] \times [NO] \quad (6)$$

$$HO_2\ turnover = [HO_2] \times [NO] \quad (7)$$

where $[OH]$, $[RO_2]$, $[NO]$, and $[HO_2]$ represent the radical and NO concentrations, and k_{OH} is the total VOC OH reactivity. Note that the total OH reactivity measured by the LIF instrument (Text S4.5) includes contributions from inorganic species and may therefore have biased the calculated VOC turnover. Furthermore, while RO_2 and HO_2 turnover were dominated by reactions with NO, their fates were not exclusively NO-driven. Comparison of radical concentrations and turnover rates between the CHANEL and JULIAC campaigns are shown in Fig. S8.

Supplementary Figures

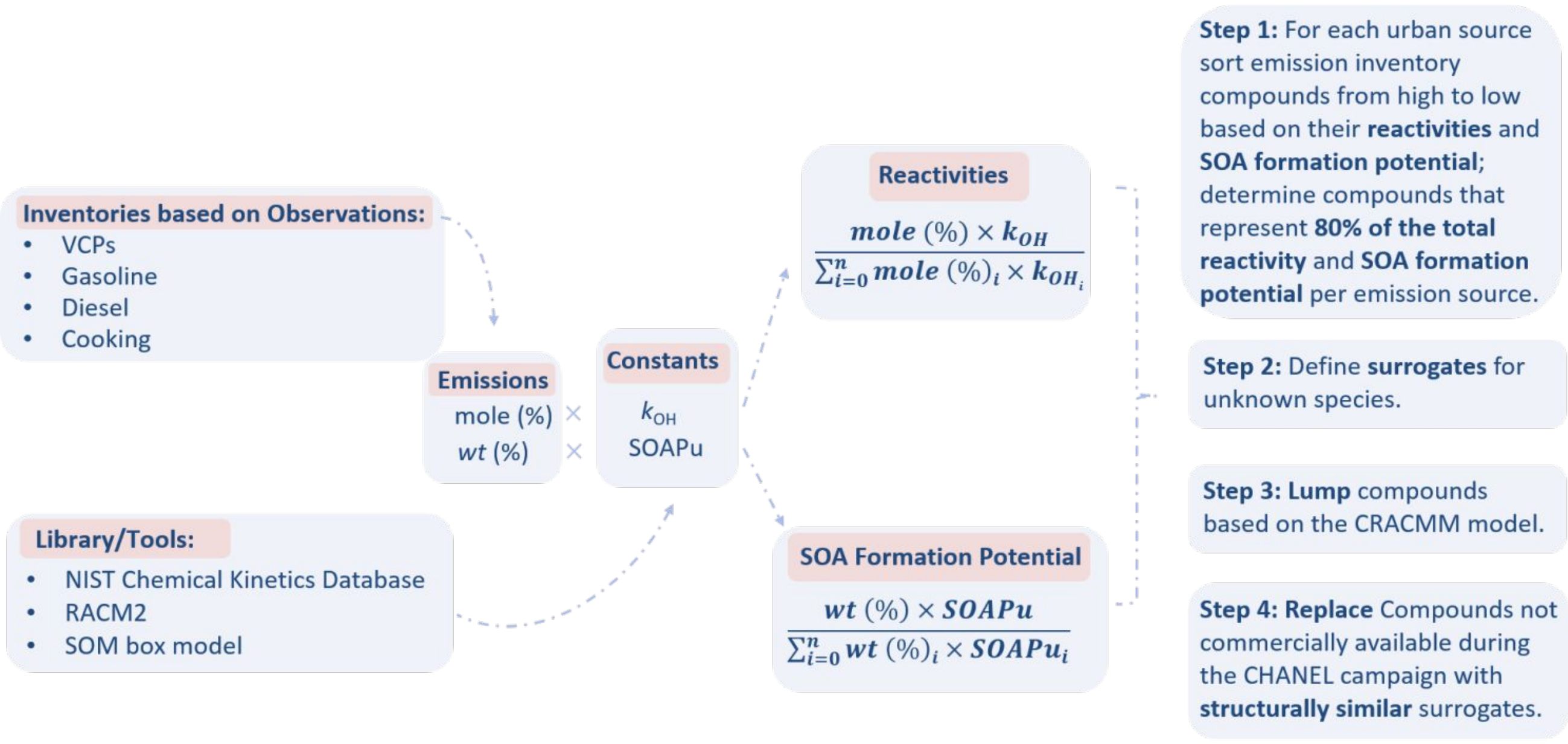

Figure S1. Workflow for constructing condensed emission fingerprints of urban sources. Schematic showing the four-step procedure used to generate reduced-complexity chemical fingerprints for volatile chemical products (VCPs), cooking, gasoline, and diesel exhaust.[1,15–19] Compounds are ranked by OH reactivity and secondary organic aerosol formation potential (SOAP), filtered to capture 80% of each, and grouped using CRACMM chemical classes. Details of compound selection and surrogates are provided in the Supplementary Spreadsheet.

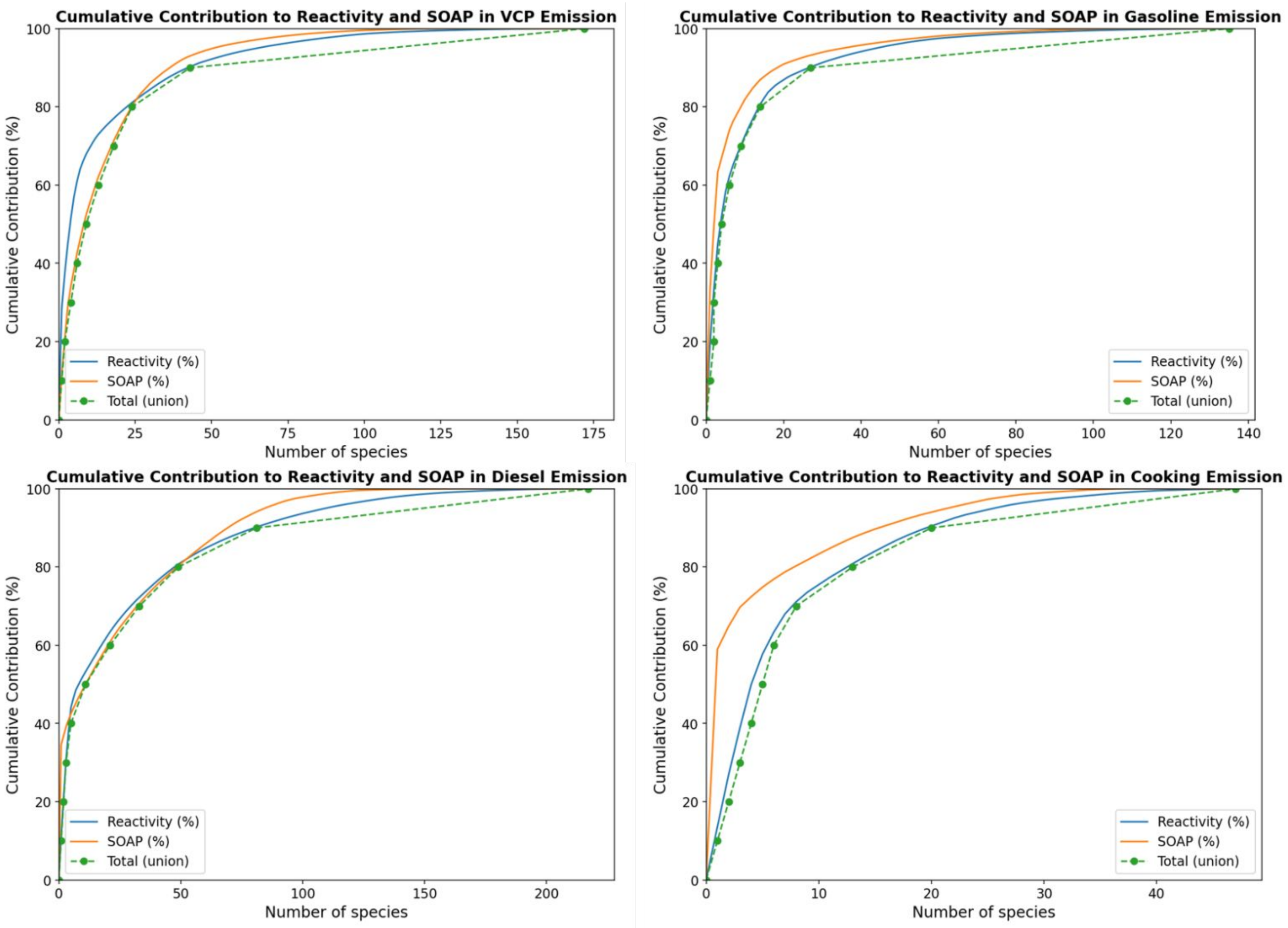

Figure S2. Cumulative contribution of individual species to OH reactivity and SOAP for inventories of VCPs, gasoline, diesel, and cooking. Species are ranked in descending order separately for each metric, and the cumulative percentage is plotted against the number of species included (blue: Reactivity; orange: SOAP). The green dashed line ("Total") shows the minimum number of unique species required to simultaneously account for a given percentage threshold of both Reactivity and SOAP, calculated as the union of the top-ranked species from each individual ranking.

As shown in Figure S2, an 80% threshold represents a reasonable compromise between the number of species retained and the representativeness of reactivity and SOAP for the emission source. Below this threshold, small increases in the number of species yield substantial gains in cumulative contribution, whereas above it, additional species add comparatively little. For example, 49 species from the diesel inventory are sufficient to reach 80% of both reactivity and SOAFP, while 81 species are needed to reach 90% for both metrics.

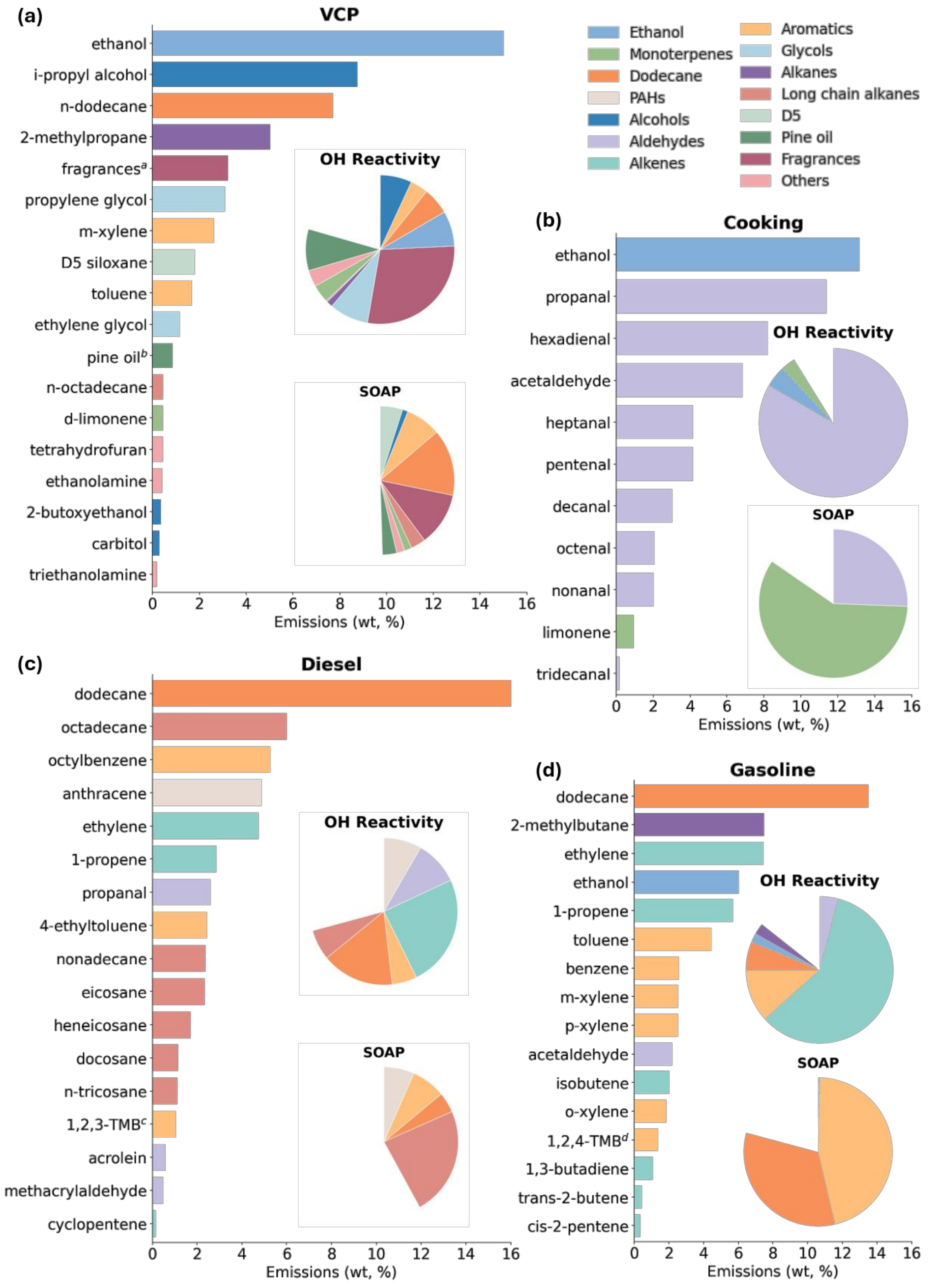

Figure S3. Injected chemical fingerprints of major urban emission sources including (a) volatile chemical products (VCP), (b) cooking, (c) diesel tailpipe, and (d) gasoline tailpipe emissions[1,15–19] during the CHANEL campaign. Bars show individual compound contributions to total source mass (by weight), and pie charts indicate the fractions of OH

reactivity and secondary organic aerosol (SOA) formation potential retained relative to the full emission inventories. In the figure, [a] fragrances denote mixtures consisting of 20% monoterpenes, 80% propylene glycol; [b] pine oil denotes a mixture of 25% a-pinene, 25% b-pinene, 50% limonene; [c] 1,2,3-TMB denotes 1,2,3-trimethylbenzene; [d] 1,2,4-TMB denotes 1,2,4-trimethylbenzene.

Fewer than 20 VOCs were injected to represent each urban emission source to the SAPHIR chamber, while they preserve more than 75% of source OH reactivity and SOA potential (except diesel, limited by high inventory uncertainties, and VCPs). Due to limited availability of several key compounds (e.g., methylcycloundecane and methylcyclodecane) during preparation of the liquid injection mixtures, the effective VCP representation in the CHANEL experiments was reduced to roughly 50 % of the inventory-based SOAP.

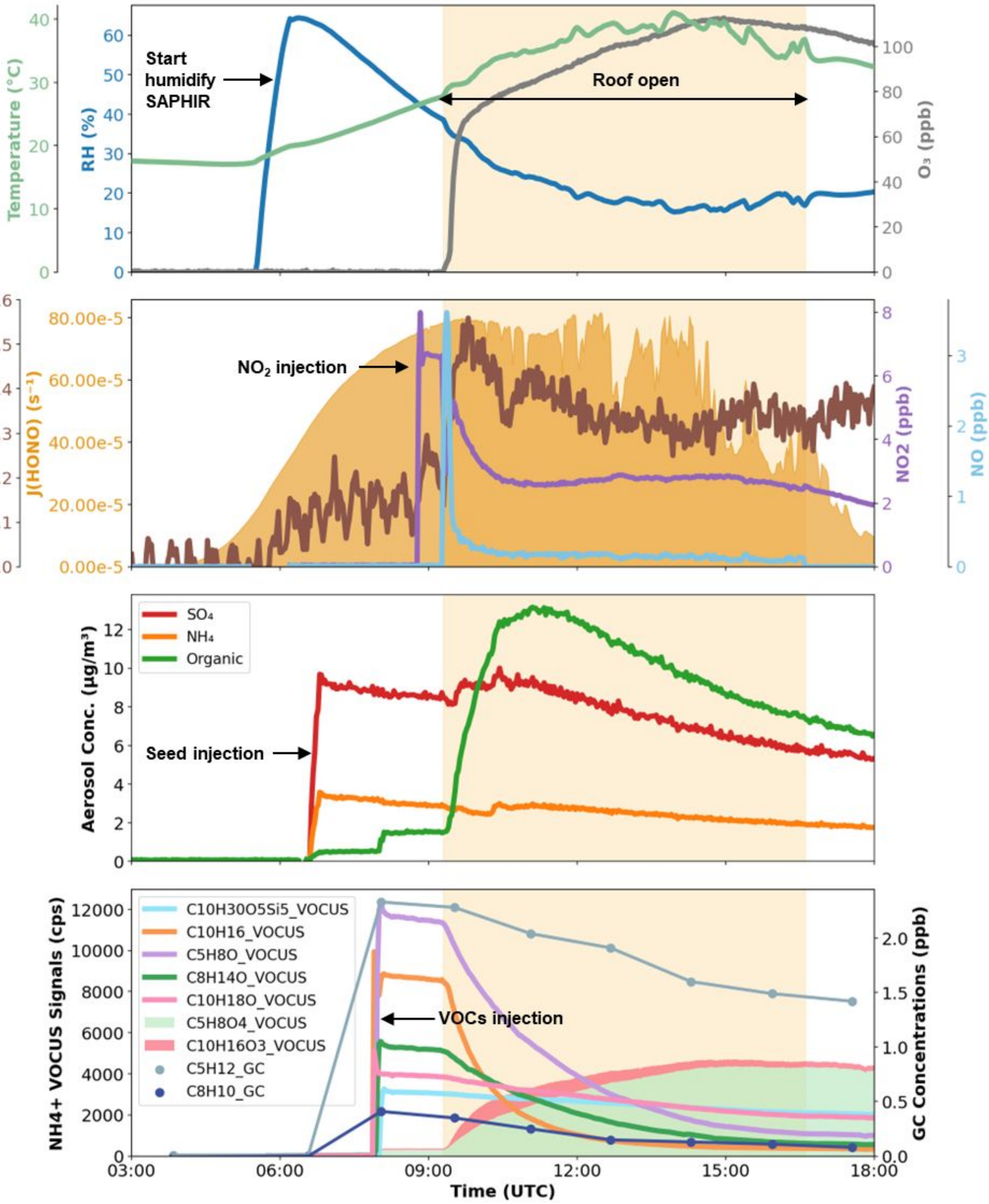

Figure S4. Time series of key parameters during a representative daytime experiment (Experiment 24 in Table S10). Panels show (from top to bottom) temperature, relative humidity (RH) and O_3; NO, NO_2, HONO, and photolysis frequencies (J(HONO)); aerosol composition (sulfate, ammonium, and organic mass); and selected VOC concentrations measured by GC-MS/FID and signals measured by NH_4^+ VOCUS. Arrows mark major experimental events, including chamber humidification, seed aerosol addition, NO_2 injection, roof opening, and VOC injection. Shaded regions indicate the photochemical phase of the experiment.

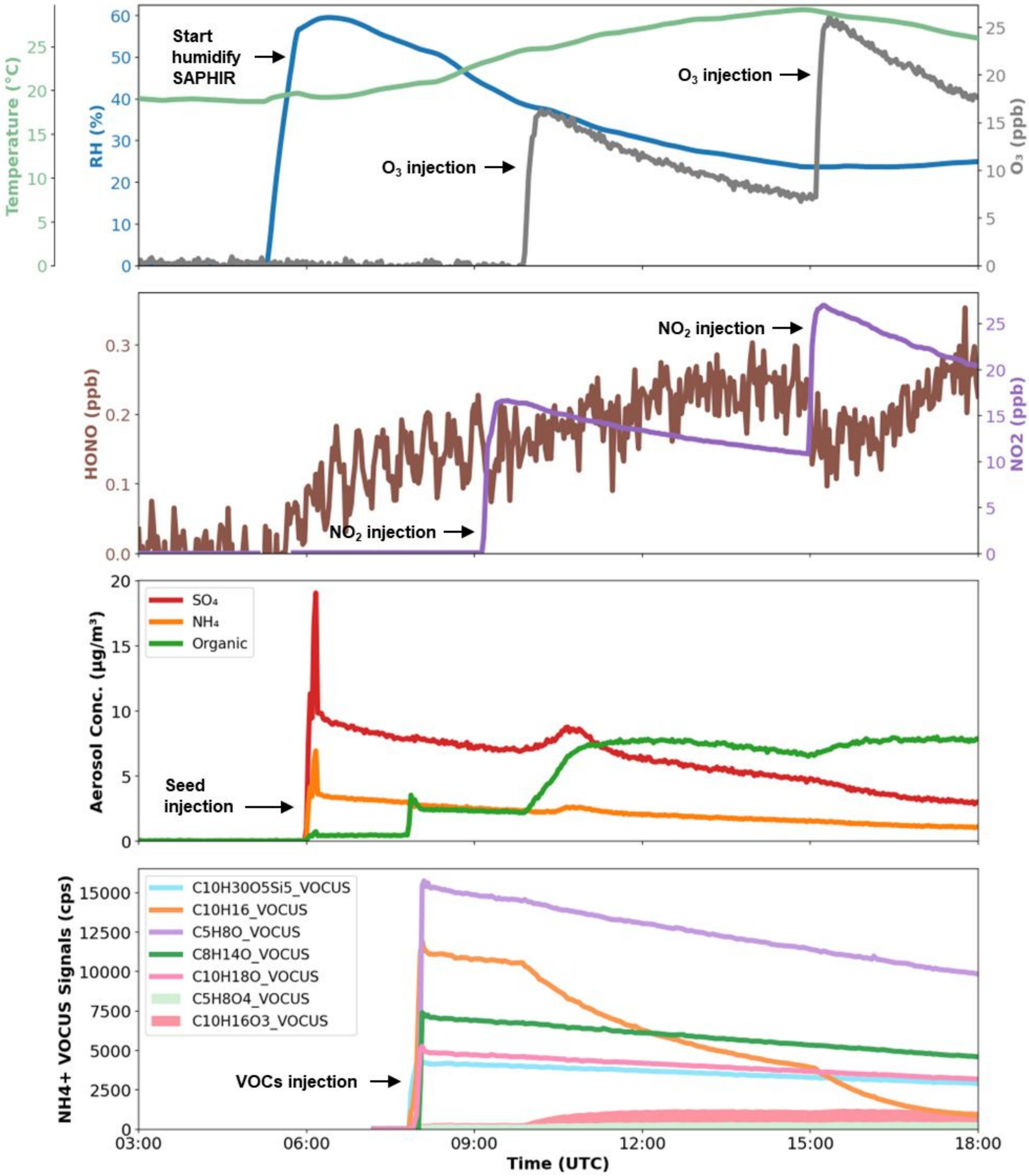

635 **Figure S5. Time series of key parameters during a representative nighttime experiment (Experiment 16 in Table S10).** Panels show (from top to bottom) temperature, relative humidity (RH) and O_3; NO_2 and HONO; aerosol composition (sulfate, ammonium, and organic mass); and selected VOC signals measured by NH_4^+ VOCUS. Arrows mark major experimental events, including chamber humidification, seed aerosol addition, O_3, NO_2, and VOC injection.

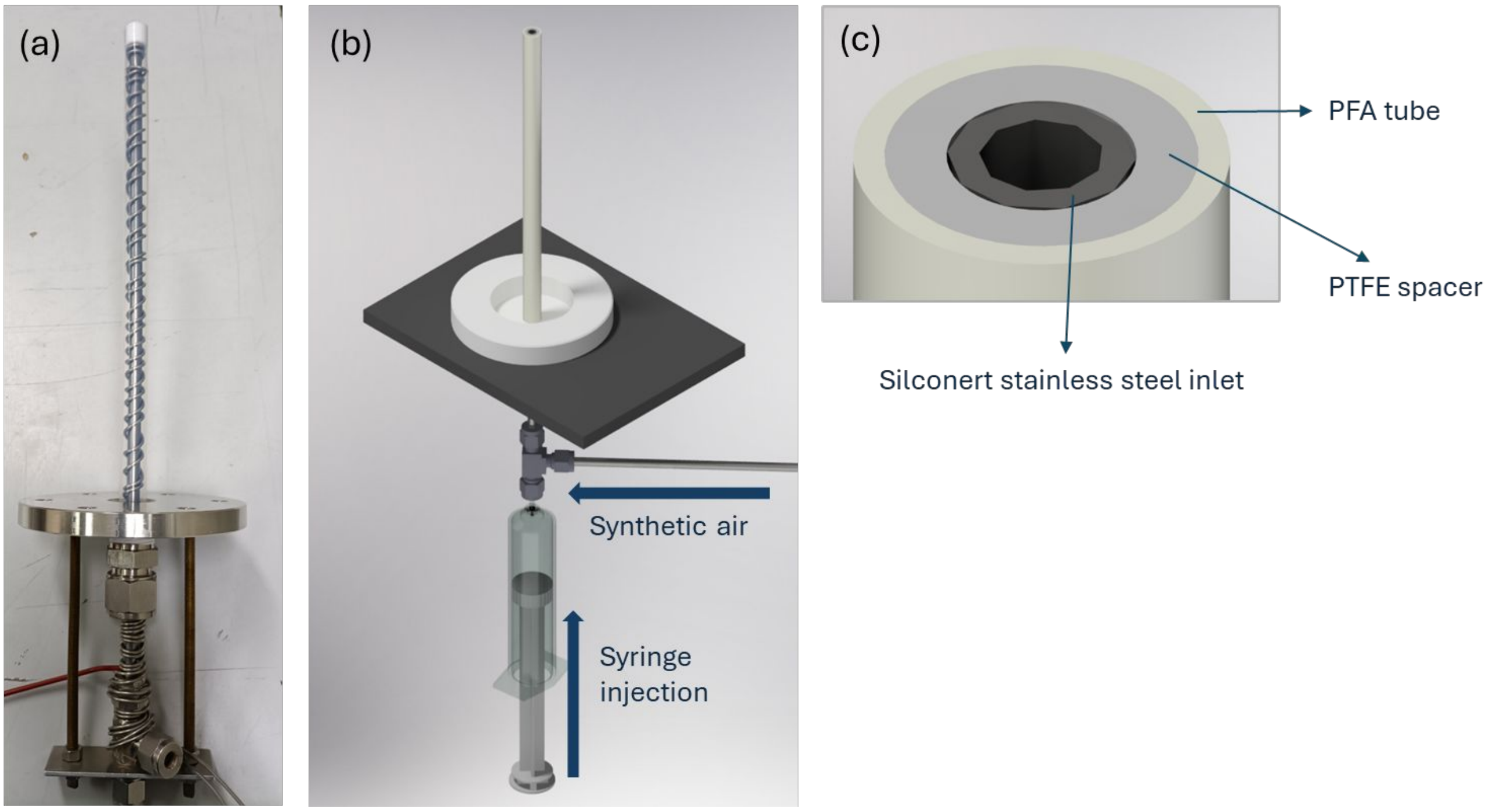

Figure S6. Optimized inlet for liquid VOC injections.

(a) Photograph of the inlet used during the CHANEL campaign. (b) 3D schematic showing the inlet setup for syringe-based liquid injections with synthetic air. (c) Cross-sectional view of the three-layer design: an inner SilcoNert-coated stainless steel tube (heated to 60 °C during CHANEL to prevent condensation), a PTFE spacer for alignment and houses a heated transfer line between the inner stainless steel tube and the outer PFA layer, and an outer PFA layer for compatibility with the SAPHIR chamber.

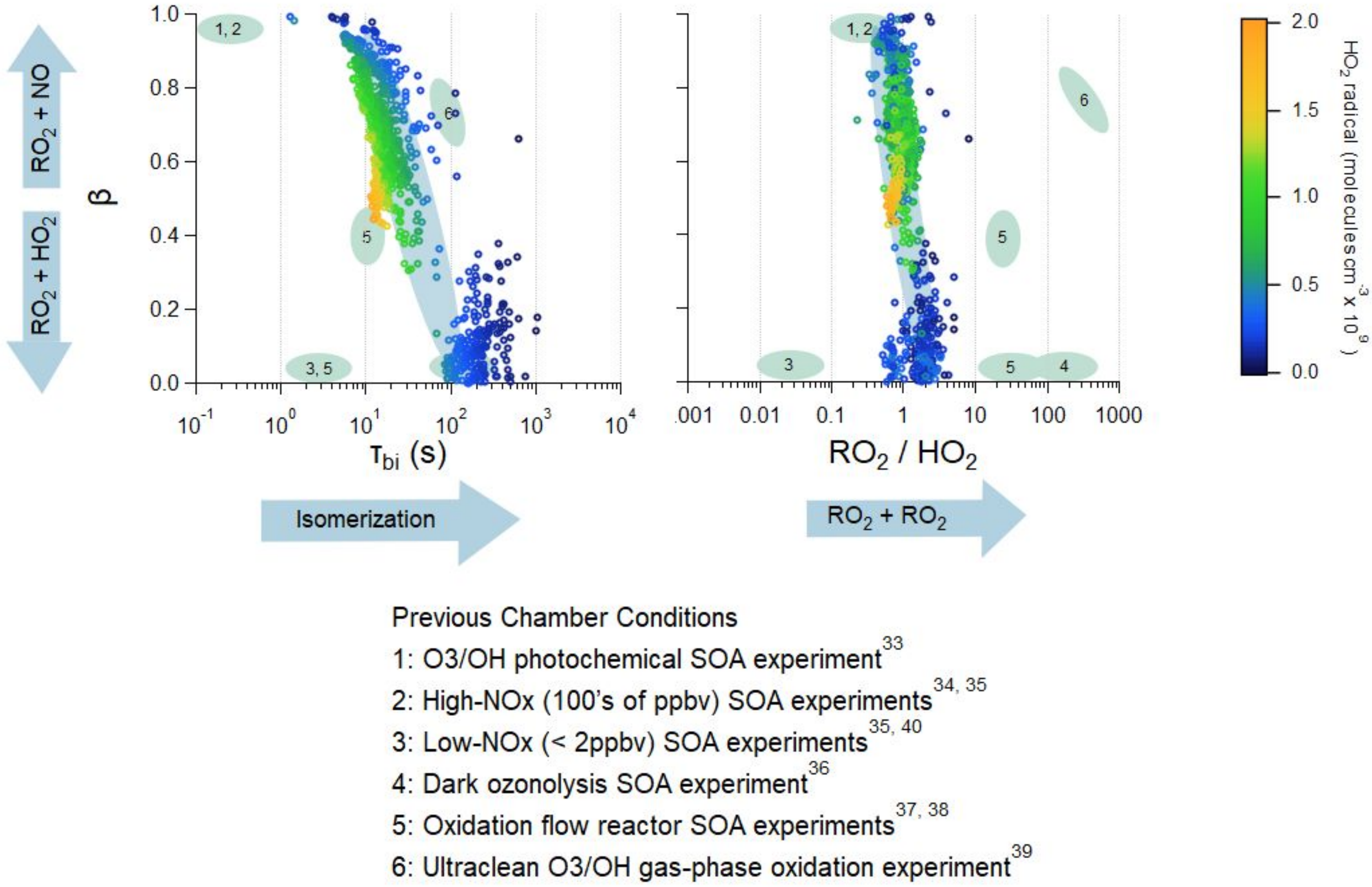

Figure S7. Radical termination regimes during CHANEL compared with global ambient distributions. CHANEL data points are coloured by measured HO_2 concentrations. Blue oval indicates global distributions from the GEOS-Chem chemical transport model.[32] Numbers in turquoise ovals correspond to previously reported chamber experiments.[33–40]

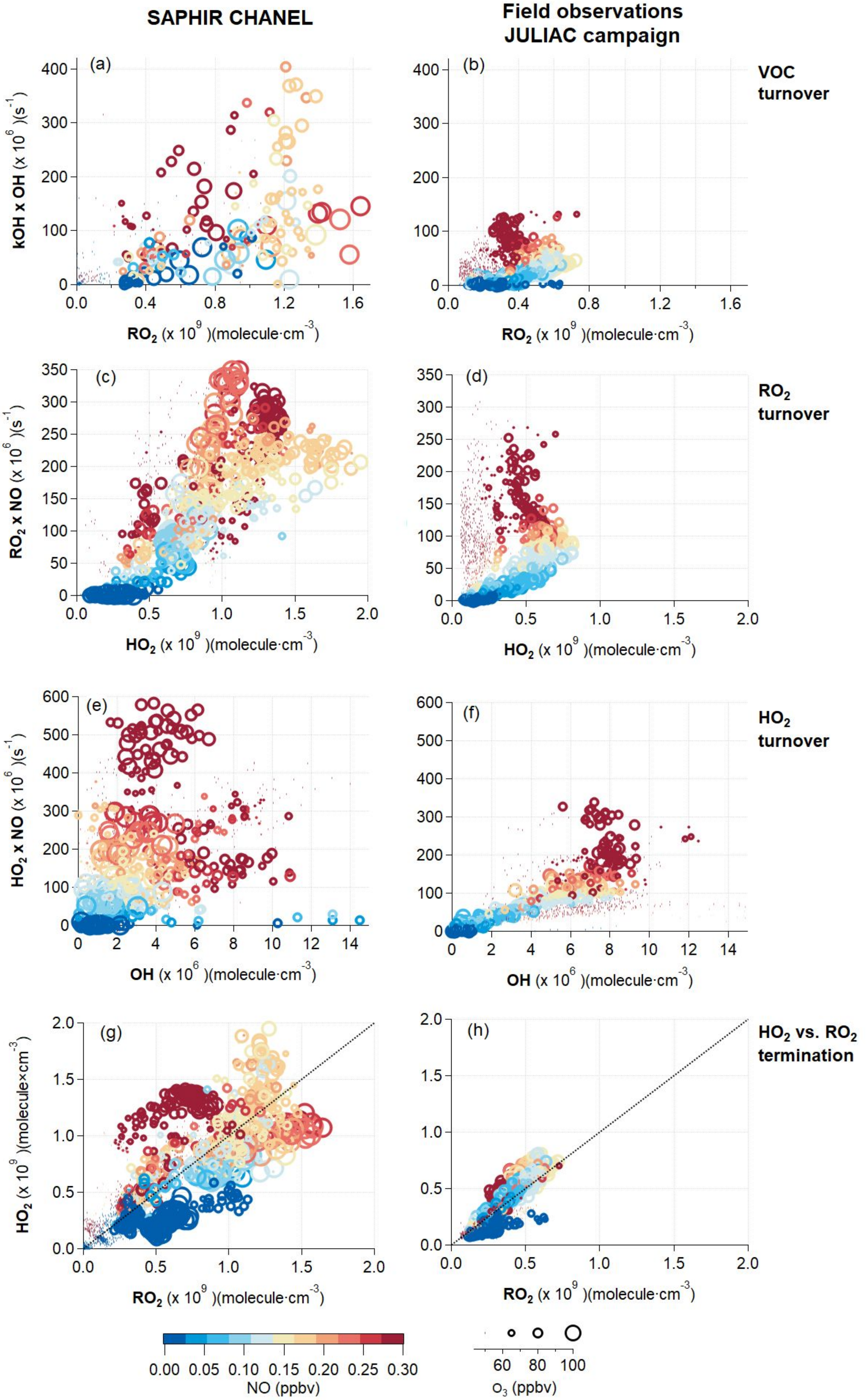

Figure S8. Radical concentrations in CHANEL experiments compared with field observations during the JULIAC campaign.

Panels (a, c, e, g) show SAPHIR CHANEL results and (b, d, f, h) JULIAC field data. (a, b) VOC turnover versus RO_2 concentration, (c, d) RO_2 turnover versus HO_2 concentration, (e, f) HO_2 turnover versus OH concentration, and (g, h) HO_2 concentration versus RO_2 concentration with the 1:1 line indicating radical termination balance. Data points are colored by NO concentration and scaled by O_3 concentration.

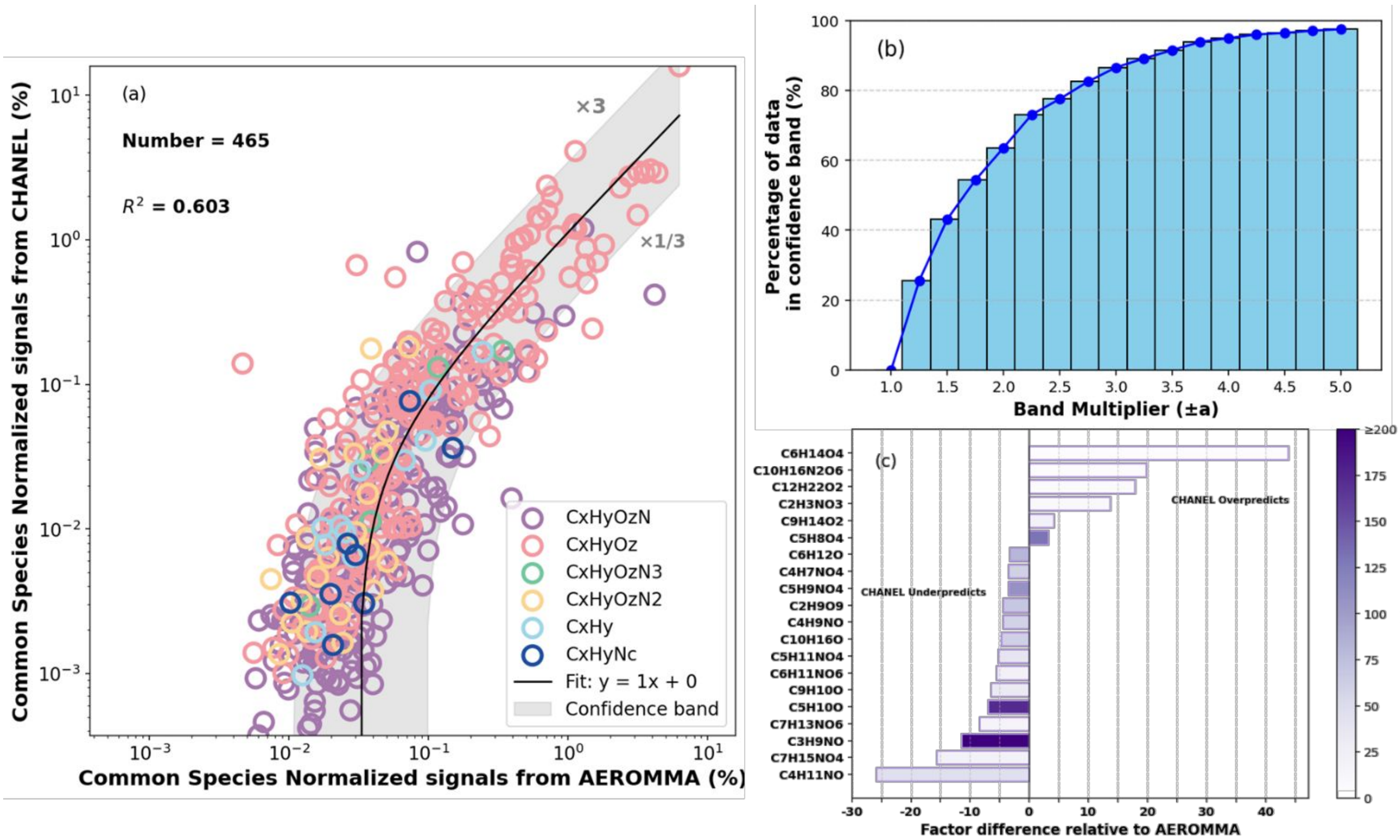

Figure S9. Comparison of VOC signals between CHANEL and AEROMMA Los Angeles measurements.

(a) Correlation of 465 common VOCs measured during the CHANEL LA Anthropogenic+ Biogenic + O_3 Daytime at High NO experiment (Experiment 24) and in urban Los Angeles air during AEROMMA. Data points are colored by chemical group. The black line shows the best-fit correlation (R^2 = 0.60); grey lines indicate ±3× deviations, and shading denotes the confidence band. (b) Fraction of common VOCs falling within factors of 1–5 of the fit. (c) VOCs deviating from the fitting by more than 3×, with bars colored by their AEROMMA signal intensity.

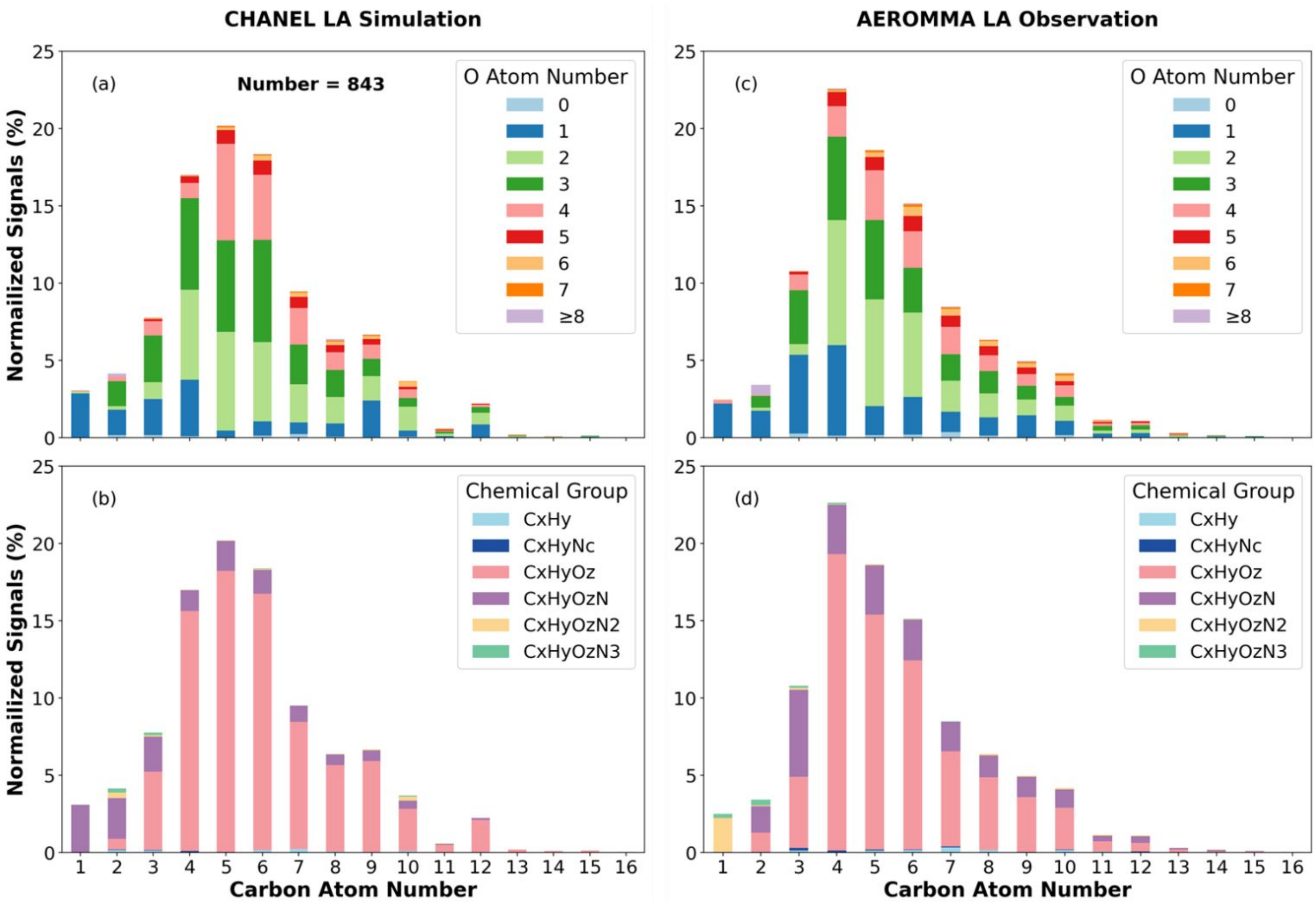

Figure S10. Comparison of VOC composition between CHANEL and AEROMMA excluding $C_3H_6O_2$.

Elemental (a,c) and chemical-group (b,d) distributions of VOCs measured by NH_4^+ VOCUS in (a,b) the CHANEL LA Anthropogenic+ Biogenic + O_3 Daytime at High NO experiment (Experiment 24) and (c,d) Los Angeles urban air during the AEROMMA campaign. CHANEL VOCs were averaged over the final two hours of oxidation to represent secondary products. Acetone (C_3H_6O), acetonitrile (C_2H_3N), ethanol (C_2H_6O), injected species, and seed compounds are excluded. **In comparison with Fig.3, $C_3H_6O_2$ is excluded here to improve the match between CHANEL and AEROMMA.**

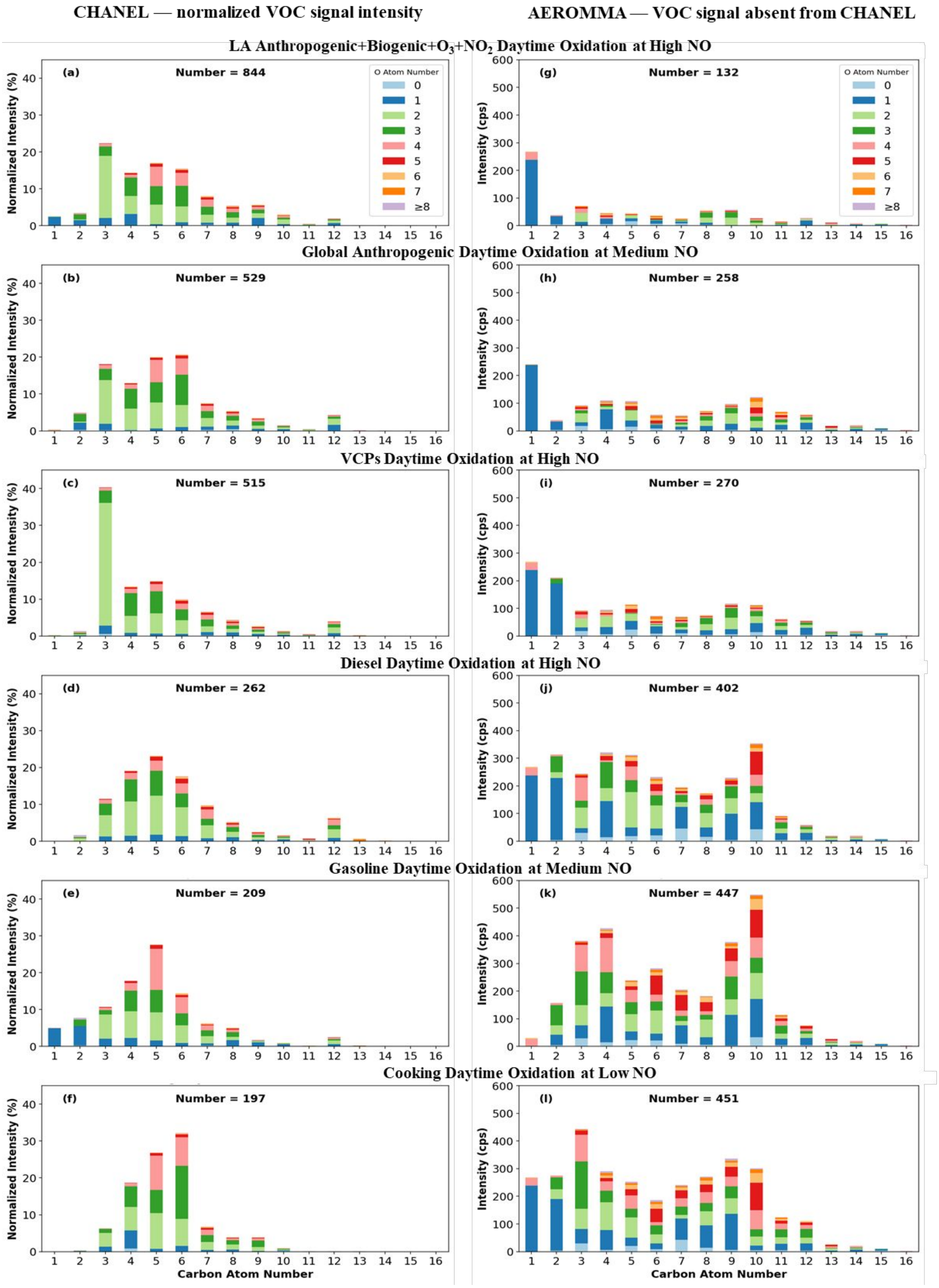
CHANEL — normalized VOC signal intensity
AEROMMA — VOC signal absent from CHANEL
LA Anthropogenic+Biogenic+O3+NO2 Daytime Oxidation at High NO
Global Anthropogenic Daytime Oxidation at Medium NO
VCPs Daytime Oxidation at High NO
Diesel Daytime Oxidation at High NO
Gasoline Daytime Oxidation at Medium NO
Cooking Daytime Oxidation at Low NO
(a) Number = 844
(b) Number = 529
(c) Number = 515
(d) Number = 262
(e) Number = 209
(f) Number = 197
(g) Number = 132
(h) Number = 258
(i) Number = 270
(j) Number = 402
(k) Number = 447
(l) Number = 451
O Atom Number
0
1
2
3
4
5
6
7
≥8
Normalized Intensity (%)
Intensity (cps)
Carbon Atom Number

Figure S11. Carbon atom number distributions of VOCs measured by NH_4^+ VOCUS in CHANEL chamber experiments and of species observed over Los Angeles during AEROMMA that were absent from the corresponding CHANEL experiment, coloured by oxygen atom number.

Left panels (a–f): CHANEL — normalized VOC distributions. Bars show the normalized signal intensity (% of total) of VOCs detected during the final two hours of oxidation in each CHANEL experiment, representing predominantly secondary oxidation products. The total number of detected VOCs is given at the top of each panel.

Right panels (g–l): AEROMMA — signal intensity of species not reproduced by CHANEL. Bars show the absolute signal intensity (cps) of VOCs observed in LA urban air during AEROMMA that were absent from the corresponding CHANEL experiment. The count of these non-overlapping species is given at the top of each panel.

Panel pairs correspond to: (a,g) LA Anthropogenic+Biogenic+O_3+NO_2 Daytime Oxidation at High NO (Experiment 24); (b,h) Global Anthropogenic Daytime Oxidation at Medium NO (Experiment 19); (c,i) VCPs Daytime Oxidation at High NO (Experiment 6); (d,j) Diesel Daytime Oxidation at High NO (Experiment 12); (e,k) Gasoline Daytime Oxidation at Medium NO (Experiment 8); and (f,l) Cooking Daytime Oxidation at Low NO (Experiment 10). Acetone (C_3H_6O), acetonitrile (C_2H_3N), ethanol (C_2H_6O), injected species, and seed compounds are excluded from both datasets.

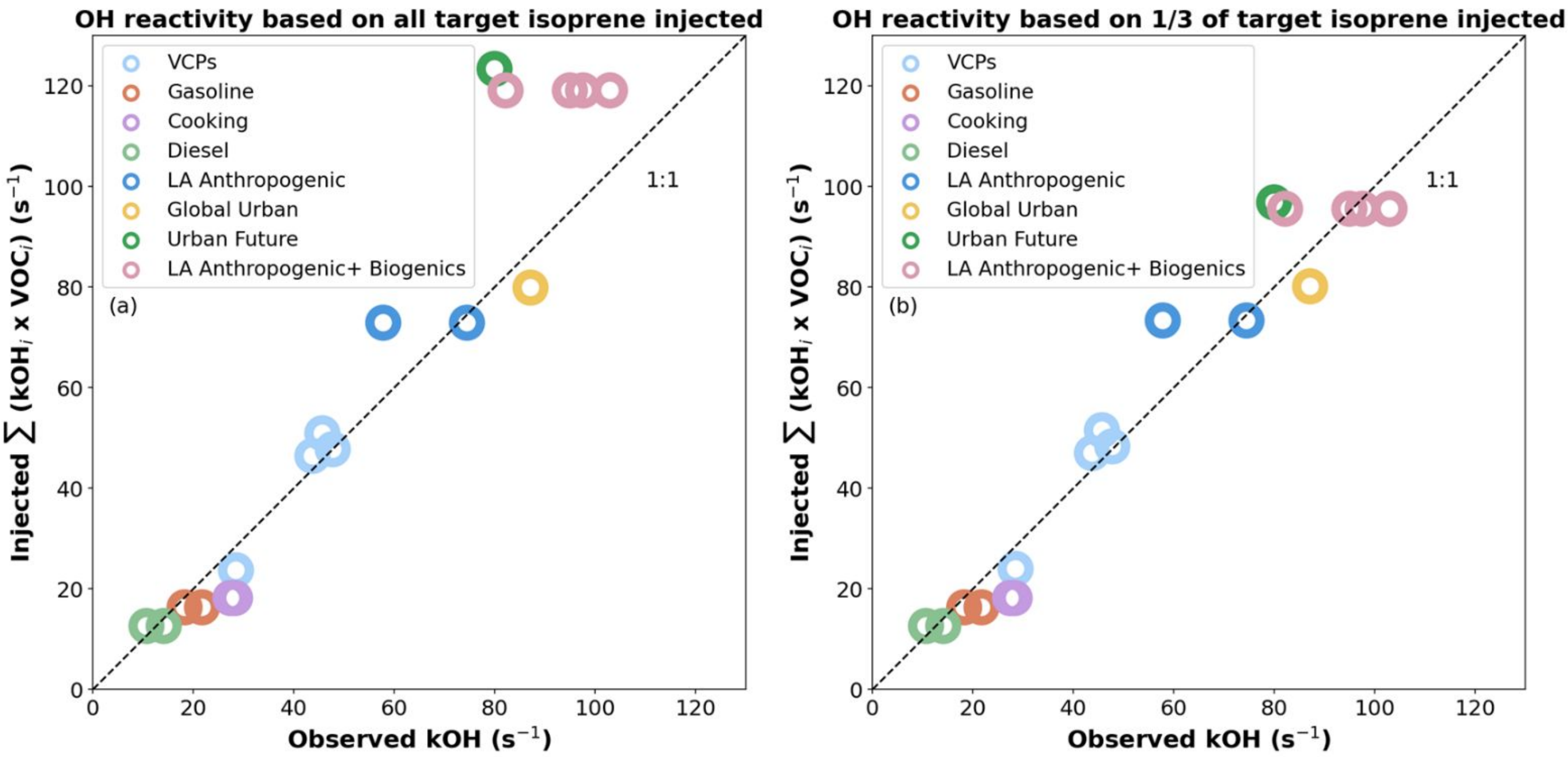

Figure S12. Comparison of observed and expected OH reactivity after VOC injections.

Observed total OH reactivity (kOH, s^{-1}) from LIF versus the expected Σ (kOH_i × $[VOC]_i$), calculated from literature OH rate constants[41,42] and (a) target injection concentrations and (b) 1/3 of target injection concentration of isoprene, and the target of all the other injected compounds. Datapoints are coloured by experiment injections (Table S1-S9); the dashed line denotes 1:1 agreement. Deviations from the 1:1 line were driven primarily by under-injection of isoprene. When accounting for the reduced isoprene injection, nearly all experiments agree with the calculated OH reactivity, except for Experiment 21 (green) and Experiment 23 (pink), consistent with the GC–MS/FID results.

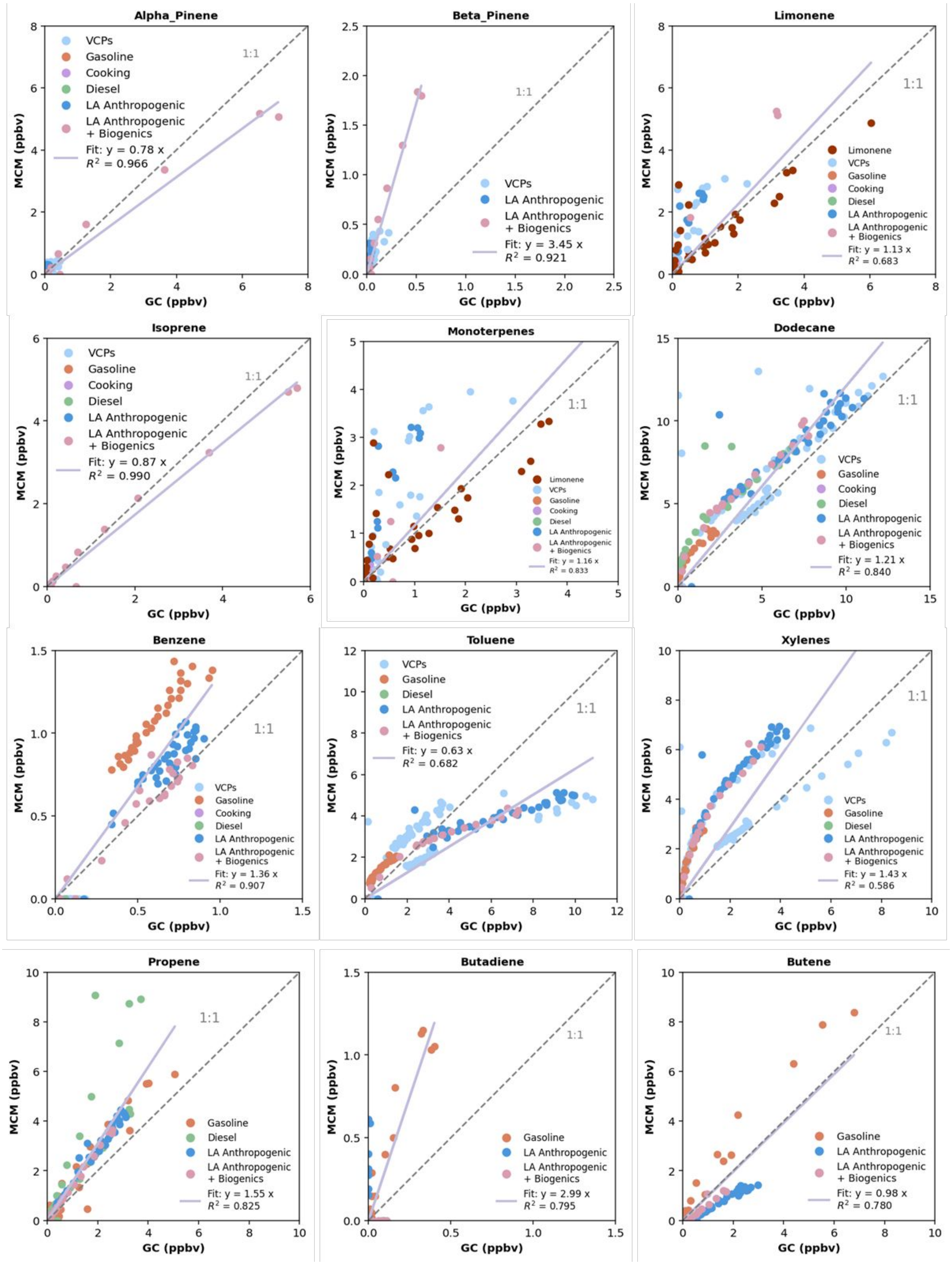

Figure S13. Comparison between MCM modelled and measured VOCs concentrations by GC–MS/FID in all experiments during the CHANEL campaign. Datapoints are coloured by experiment injections (Table S1-S9); the grey dashed line denotes 1:1 agreement; and the purple solid line represents the linear fit for the data. For each experiment, the

MCM was initialized using the full set of injected VOCs (but 1/3 of the target isoprene), with initial concentrations constrained by the expected experimental conditions upon injection.

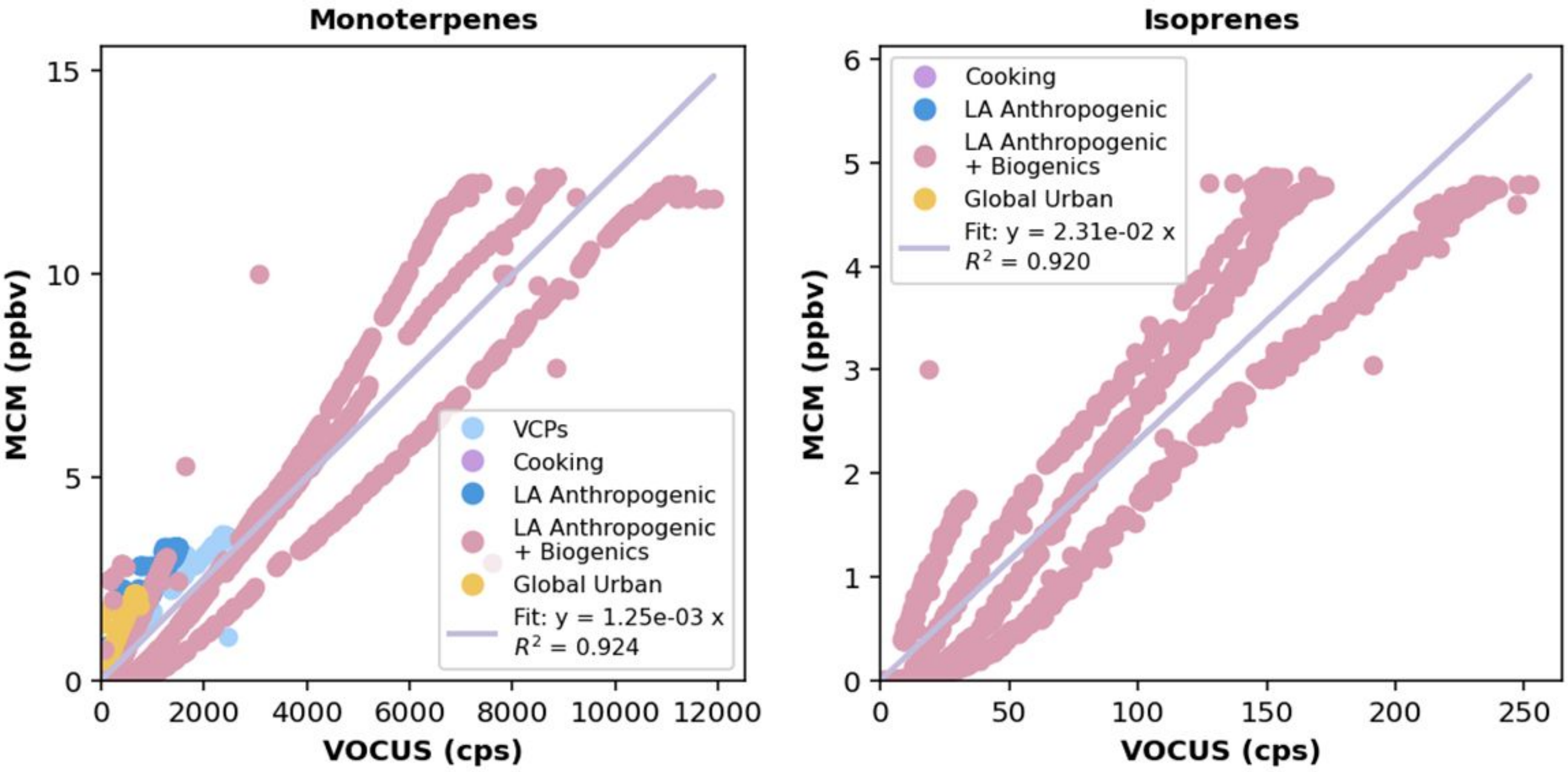

Figure S14. Comparison between MCM modelled concentrations and measured signals by NH_4^+ VOCUS in all experiments during the CHANEL campaign. Datapoints are coloured by experiment injections (Table S1-S9); the purple solid line represents the linear fit for the data. For each experiment, the MCM was initialized using the full set of injected VOCs (but 1/3 of the target isoprene), with initial concentrations constrained by the expected experimental conditions upon injection.

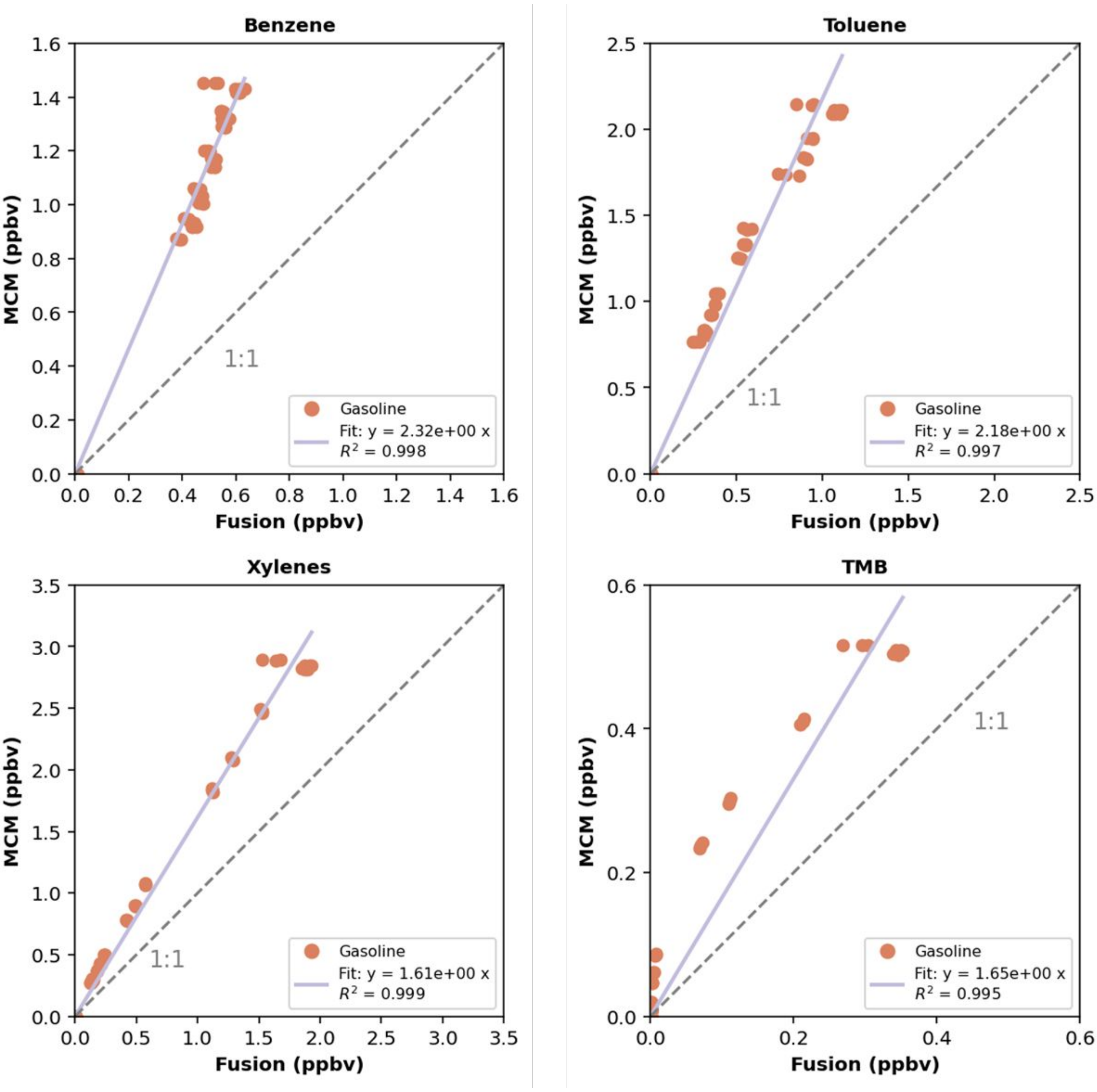

Figure S15. Comparison between MCM modelled concentrations and measured concentrations by H_3O^+ FUSION in the gasoline experiment during the CHANEL campaign. The grey dashed line denotes 1:1 agreement; and the purple solid line represents the linear fit for the data. The MCM was initialized using the full set of injected VOCs, with initial concentrations constrained by the expected experimental conditions upon injection.

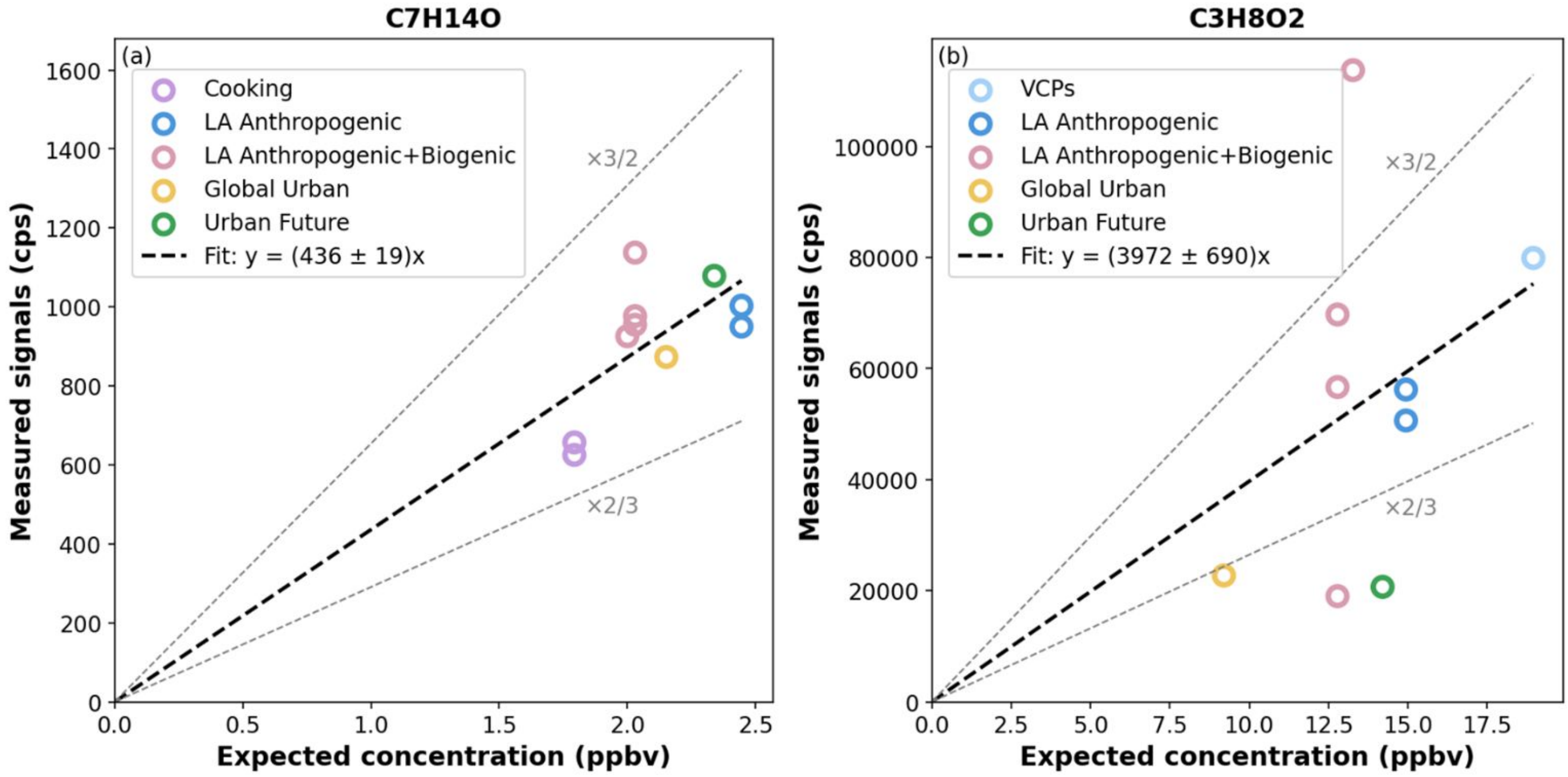

Figure S16. Comparison of measured and expected concentrations of (a) heptanal ($C_7H_{14}O$) and (b) propylene glycol ($C_3H_8O_2$) during CHANEL experiments.

Measured NH_4^+ VOCUS signal intensities versus expected gas-phase concentrations for heptanal injections from the cooking fingerprint, and propylene glycol injections from the VCP fingerprint. The black dashed line shows the linear fit (y = (436 ± 19) x for heptanal, y = (3972±690) x for propylene glycol), and grey dashed lines indicate ±50 % deviation. Results show good agreement of heptanal injections across different mixture experiments, confirming stable injection and detection performance for this compound. However, the variability of propylene glycol injections across different experiments, particularly lower signals in Experiment 21 (green) and Experiment 23 (pink), likely results from losses during injection due to the high viscosity of propylene glycol.

Supplementary Tables

Table S1 | Detailed composition of the volatile chemical products (VCP) fingerprint and injection.

List of injected compounds representing VCPs emissions used in the CHANEL campaign, with the original fingerprint species that are generated from step 1-3 (Text 2.1 and S1) and assigned CRACMM surrogates. Columns show total emissions by weight and mole fraction, OH rate constants (kOH, from the emission inventory[15]), individual SOAP values (SOAPu), and fractional contributions to total OH reactivity and secondary organic aerosol formation potential (SOAP) from the full emission inventory.[15] More details of fingerprint compounds are shown in the Supplementary spreadsheet.

**Injected Compounds based on availability**	**Fingerprint Compounds**	**CRACMM Surrogate**	**Total Emissions (wt) %**	**Total Emissions (mol) %**	**kOH**	**OH Reactivity (%)**	**SOAPu**	**SOAP (%)**
pine oil (25% a-pinene, 25% b-pinene, 50% limonene)	pine oil (25% a-pinene, 25% b-pinene, 50% limonene)	API, LIM	0.86	0.47	4.79	9.02	0.80	3.10
ethanol	ethanol	EOH	15.01	24.18	0.08	7.66	0.00	0.00
ethylene glycol	ethylene glycol	ETEG	1.18	1.41	0.37	2.08	0.00	0.00
tetrahydrofuran	tetrahydrofuran	HC10	0.44	0.45	0.44	0.81	0.00	0.00
2-methylpropane	2-methylpropane	HC3	5.02	6.41	0.05	1.35	0.00	0.00
d-limonene	d-limonene	LIM	0.44	0.24	4.04	3.93	0.80	1.60
fragrances (20% monoterpenes, 80% propylene glycol)	fragrances (20% monoterpenes, 80% propylene glycol)	LIM, PROG	3.22	1.75	4.04	28.46	0.80	11.62
propylene glycol	propylene glycol	PROG	3.09	3.02	0.53	6.44	0.00	0.00
carbitol	carbitol	ROCIOXY	0.30	0.18	1.01	0.74	0.52	0.70
Decamethylcyclopentasiloxane (D5)	Decamethylcyclopentasiloxane (D5)	ROCIOXY	1.81	0.36	0.03	0.05	0.60	4.90
ethanolamine	ethanolamine	ROCP1ALK	0.41	0.50	0.88	1.78	0.00	0.00
triethanolamine	triethanolamine	ROCP1ALK	0.19	0.09	2.72	1.02	2.08	1.76
i-propyl alcohol	i-propyl alcohol	ROH	8.75	10.80	0.13	5.46	0.00	0.00
2-butoxyethanol	2-butoxyethanol	ROH	0.36	0.22	0.72	0.64	0.31	0.50
toluene	toluene	TOL	1.69	1.36	0.14	0.76	0.54	4.14
m-xylene	m-xylene	XYM	2.64	1.84	0.42	3.10	0.29	3.50
n-dodecane	2-Methylundecane	HC10	7.70	4.47	0.33	5.84	0.42	14.47
	2-Methyloctane	HC10						
	Isopropylcyclopentane	HC8						
	n-dodecane	ROCP6ALK						
n-octadecane	2-Methylpentadecane	ROCP4ALK	0.46	0.15	0.51	0.30	1.54	3.20
	2-Methylhexadecane	ROCP4ALK						

Table S2 | Detailed composition of the gasoline fingerprint and injection.

List of injected compounds representing gasoline tailpipe emissions used in the CHANEL campaign, with the original fingerprint species that are generated from step 1-3 (Text 2.1 and S1) and assigned CRACMM surrogates. Columns show total emissions by weight and mole fraction, OH rate constants (kOH, from the emission inventory[15]), individual SOAP values (SOAPu), and fractional contributions to total OH reactivity and secondary organic aerosol formation potential (SOAP) from the full emission inventory.[15] More details of fingerprint compounds are shown in the Supplementary spreadsheet.

Injected Compounds based on availability	Fingerprint Compounds	CRACMM Surrogate	Total Emissions (wt, %)	Total Emissions (mole, %)	kOH	OH Reactivity (%)	SOAPu	SOAP (%)
acetaldehyde	acetaldehyde	ACD	2.20	3.20	14.90	4.02	0.00	0.00
1,3-butadiene	1,3-butadiene	BDE13	1.07	1.26	66.50	7.07	0.00	0.00
benzene	benzene	BEN	2.56	2.10	1.22	0.22	0.18	16.43
ethanol	ethanol	EOH	6.04	8.40	2.81	1.99	0.00	0.00
ethylene	ethylene	ETE	7.47	17.05	8.20	11.78	0.00	0.00
2-methylbutane	2-methylbutane	HC5	7.48	6.64	4.44	2.48	0.00	0.02
trans-2-butene	trans-2-butene	OLI	0.45	0.51	71.10	3.05	0.00	0.00
cis-2-pentene	cis-2-pentene	OLI	0.35	0.32	71.10	1.92	0.03	0.32
1-propene	1-propene	OLT	5.70	8.37	30.60	21.58	0.00	0.00
isobutene	isobutene	OLT	2.04	2.33	71.10	13.93	0.00	0.00
dodecane	dodecane	ROCP6	13.51	6.55	11.30	6.23	0.07	32.82
toluene	toluene	TOL	4.46	3.10	5.93	1.55	0.09	14.10
1,2,4-trimethylbenzene	1,2,4-trimethylbenzene	XYM	1.40	0.75	23.10	1.45	0.07	3.61
p-xylene	p-xylene	XYE	2.53	1.49	21.30	2.68	0.05	4.34
	4-Ethyltoluene or 3-Ethyltoluene	XYE + XYM						
o-xylene	o-xylene	XYE	1.87	1.09	13.60	1.25	0.05	3.20
	4-Ethyltoluene or 3-Ethyltoluene	XYE + XYM						
m-xylene	m-xylene	XYM	2.53	2.31	23.10	4.49	0.05	4.34
	4-Ethyltoluene or 3-Ethyltoluene	XYE + XYM						

Table S3 | Detailed composition of the diesel fingerprint and injection.

List of injected compounds representing diesel tailpipe emissions used in the CHANEL campaign, with the original fingerprint species that are generated from step 1-3 (Text 2.1 and S1) and assigned CRACMM surrogates. Columns show total emissions by weight and mole fraction, OH rate constants (kOH, from the emission inventory[15]), individual SOAP values (SOAPu), and fractional contributions to total OH reactivity and secondary organic aerosol formation potential (SOAP) from the full emission inventory.[15] More details of fingerprint compounds are shown in the Supplementary spreadsheet.

Injected Compounds based on availability	Fingerprint Compounds	CRACMM Surrogate	Total Emissions (wt, %)	Total Emissions (mole, %)	kOH	OH Reactivity (%)	SOAPu	SOAP (%)
acrolein	acrolein	ACRO	0.58	0.79	29.00	2.09	0.00	0.00
propanal	propanal	ALD	2.60	3.42	19.80	6.21	0.00	0.00
ethylene	ethylene	ETE	4.75	12.91	8.20	9.71	0.00	0.00
cyclopentene	cyclopentene	OLI	0.17	0.19	71.10	1.22	0.01	0.00
1-propene	1-propene	OLT	2.86	4.90	30.60	13.75	0.00	0.00
Docosane	Docosane	ROCP1	1.14	0.88	0.00	0.00	0.98	3.11
n-Tricosane	n-Tricosane	ROCP2	1.13	0.87	0.00	0.00	1.02	3.23
Eicosane	Eicosane	ROCP2	2.34	1.65	0.00	0.00	0.66	4.34
nonadecane	nonadecane	ROCP3	2.36	1.82	11.30	1.89	0.47	3.08
heneicosane	heneicosane	ROCP3	1.72	1.21	0.00	0.00	0.86	4.13
octylbenzene	octylbenzene	ROCP5ARO	5.26	4.41	5.93	2.40	0.46	6.69
1,2,3-Trimethylbenzene	1,2,3-Trimethylbenzene	XYM	1.06	0.89	5.93	0.49	0.07	0.22
dodecane	dodecane	ROCP6	22.90	15.36	11.30	15.92	0.07	4.39
	2-Methyldecane	HC8						
	2-Methylundecane	HC8						
	2-methyltridecane	ROCP6						
octadecane	octadecane	ROCP4	6.00	4.63	11.30	4.80	0.34	5.73
	2-methylpentadecane	ROCP4						
	2-methylhexadecane	ROCP4						
Methacrylaldehyde	Methacrylaldehyde	MACR	0.50	0.54	29.00	1.45	0.00	0.00
	crotonaldehyde	MACR						
Anthracene	1-Butyl-4-methylnaphthalene	NAPH	4.88	3.91	23.10	8.29	0.49	6.60
	Chrysene	NAPH						
	5-Methylchrysene	NAPH						
	Cyclohexadecane	ROCP2						
	cyclopentadecane	ROCP3						
4-Ethyltoluene	1,3,5-triethylbenzene	ROCP6ARO	2.45	2.06	13.60	2.57	0.07	0.50
	1,3-dimethyl-5-propylbenzene	XYM						

Table S4 | Detailed composition of the cooking fingerprint and injection.

List of injected compounds representing cooking emissions used in the CHANEL campaign, with the original fingerprint species that are generated from step 1-3 (Text 2.1 and S1) and assigned CRACMM surrogates. Columns show total emissions by weight and mole fraction, OH rate constants (kOH, from the emission inventory[15]), individual SOAP values (SOAPu), and fractional contributions to total OH reactivity and secondary organic aerosol formation potential (SOAP) from the full emission inventory.[15] More details of fingerprint compounds are shown in the Supplementary spreadsheet.

Injected Compounds based on availability	Fingerprint Compounds	CRACMM Surrogate	Total Emissions (wt, %)	Total Emissions (mole, %)	kOH	Reactivity (%)	SOAPu	SOAP (%)
acetaldehyde	acetaldehyde	ACD	6.86	14.29	1.50×10^{-11}	11.92	0.00	0.00
propanal	propanal	ALD	11.39	16.16	1.99×10^{-11}	17.88	0.00	0.00
heptanal	heptanal	ALD	4.18	3.32	2.96×10^{-11}	5.46	0.00	1.99
pentenal	pentenal	ALD	4.14	4.57	4.37×10^{-11}	11.09	0.00	0.18
octenal	octenal	ALD	2.08	1.62	4.08×10^{-11}	3.68	0.00	2.41
limonene	monoterpene	API	0.97	0.66	8.52×10^{-11}	3.14	0.13	58.99
ethanol	ethanol	EOH	13.18	26.29	3.2×10^{-12}	4.68	0.00	0.00
nonanal	nonanal	ROCIOXY	2.04	1.36	2.41×10^{-11}	1.82	0.01	4.74
decanal	decanal	ROCIOXY	3.06	1.88	3.15×10^{-11}	3.29	0.01	12.58
tridecanal	tridecanal	ROCP5ARO	0.17	0.08	3.15×10^{-11}	0.14	0.03	2.43
hexadienal	hexadienal	ALD	8.20	8.56	5.90×10^{-11}	28.09	0.00	1.28
	pentadienal	ALD						

Table S5 | Detailed composition of the biogenic injection.

List of injected compounds representing biogenic emission[43] in the CHANEL campaign and their total emissions by weight fraction.

Injected Compounds	Total Emissions (wt, %)
isoprene	42%
a-pinene	24%
limonene	9%
a-cedrene	7%
camphene	5%
eucalyptol	5%
b-myrcene	4%
b-pinene	4%

Table S6 | Detailed composition of experiment simulating LA Anthropogenic in the CHANEL campaign.

Contribution of different emission sources is defined by Stockwell *et al*.[16]

**VCP:**	
Compound of Injection	Total Emissions (wt, %)
Limonene	1.85
propylene glycol	6.93
a-pinene	0.26
b-pinene	0.26
ethanol	18.35
ethylene glycol	1.44
D5 siloxane	2.22
triethanolamine	0.23
n-dodecane	9.42
i-propyl alcohol	10.70
toluene	2.07
m-xylene	3.22
carbitol	0.36
tetrahydrofuran	0.54
ethanolamine	0.51
2-butoxyethanol	0.44
octadecane	0.56
2-methylpropane	6.13
**sum**	**65.50**
**Gasoline:**	
Compound of Injection	Total Emissions (wt, %)
acetaldehyde	0.43
benzene	0.50
ethanol	1.18

dodecane	2.63
toluene	0.87
p-xylene	0.49
o-xylene	0.36
m-xylene	0.49
1,2,4-trimethylbenzene	0.27
cis-2-pentene	0.07
2-methylbutane	1.46
1-propene	1.11
ethylene	1.45
trans-2-butene	0.09
1,3-butadiene	0.21
isobutene	0.40
**sum**	**12.00**
**Cooking:**	
**Compound of Injection**	**Total Emissions (wt, %)**
acetaldehyde	2.74
propanal	4.55
heptanal	1.67
hexadienal	3.28
pentenal	1.66
octenal	0.83
nonanal	0.82
decanal	1.22
limonene	0.39
ethanol	5.27
tridecanal	0.07
**sum**	**22.50**

Table S7 | Detailed composition of experiment simulating LA Anthropogenic+Biogenic in the CHANEL campaign.

Contribution of different emission sources is defined by Stockwell *et al.*[16]

**VCP**	
**Compound of Injection**	**Total Emissions (wt, %)**
Limonene	1.59
propylene glycol	5.94
a-pinene	0.23
b-pinene	0.23
ethanol	15.74
ethylene glycol	1.24
D5 siloxane	1.90
triethanolamine	0.20
n-dodecane	8.08
i-propyl alcohol	9.17
toluene	1.78
m-xylene	2.77
carbitol	0.31
tetrahydrofuran	0.46
ethanolamine	0.43
2-butoxyethanol	0.37
octadecane	0.48
2-methylpropane	5.26
**sum**	**56.18**
**Gasoline**	
**Compound of Injection**	**Total Emissions (wt, %)**
acetaldehyde	0.36
benzene	0.42
ethanol	1.00
dodecane	2.23
toluene	0.74
p-xylene	0.42
o-xylene	0.31
m-xylene	0.42
1,2,4-trimethylbenzene	0.23
cis-2-pentene	0.06
2-methylbutane	1.24
1-propene	0.94
ethylene	1.24
trans-2-butene	0.07
1,3-butadiene	0.18
isobutene	0.34
**sum**	**10.20**
**Cooking**	
**Compound of Injection**	**Total Emissions (wt, %)**
acetaldehyde	2.34
propanal	3.88
heptanal	1.43

hexadienal	2.80
pentenal	1.41
octenal	0.71
nonanal	0.70
decanal	1.04
limonene	0.33
ethanol	4.50
tridecanal	0.06
**sum**	**19.20**
**Biogenic**	
**Compound of Injection**	**Total Emissions (wt, %)**
Isoprene	6.06
a-pinene	3.46
limonene	1.30
a-cedrene	1.01
camphene	0.72
eucalyptol	0.72
b-myrcene	0.58
b-pinene	0.58
**sum**	**14.43**

Table S8 | Detailed composition of experiment simulating Global Anthropogenic in the CHANEL campaign.

Contribution of different emission sources is defined by Stockwell *et al*.[16]

**VCP**	
**Compound of Injection**	**Total Emissions (wt, %)**
Limonene	1.13
propylene glycol	4.23
a-pinene	0.16
b-pinene	0.16
ethanol	11.21
ethylene glycol	0.88
D5 siloxane	1.35
triethanolamine	0.14
n-dodecane	5.75
i-propyl alcohol	6.53
toluene	1.27
m-xylene	1.97
carbitol	0.22
tetrahydrofuran	0.33
ethanolamine	0.31
2-butoxyethanol	0.27
octadecane	0.34
2-methylpropane	3.75
**sum**	**40.00**
**Gasoline**	
**Compound of Injection**	**Total Emissions (wt, %)**
acetaldehyde	0.71

benzene	0.83
ethanol	1.96
dodecane	4.38
toluene	1.45
p-xylene	0.82
o-xylene	0.61
m-xylene	0.82
1,2,4-trimethylbenzene	0.45
cis-2-pentene	0.11
2-methylbutane	2.43
1-propene	1.85
ethylene	2.42
trans-2-butene	0.14
1,3-butadiene	0.35
isobutene	0.66
**sum**	**20.00**
**Cooking**	
**Compound of Injection**	**Total Emissions (wt, %)**
acetaldehyde	2.44
propanal	4.05
heptanal	1.48
hexadienal	2.91
pentenal	1.47
octenal	0.74
nonanal	0.73
decanal	1.09
limonene	0.34
ethanol	4.69
tridecanal	0.06
**sum**	**20.00**
**Diesel**	
**Compound of Injection**	**Total Emissions (wt, %)**
acrolein	0.27
propanal	1.20
Methacrylaldehyde	0.23
cyclopentene	0.08
1,3,5-trimethylbenzene	1.00
dodecane	8.93
octadecane	1.03
Docosane	0.52
Eicosane	1.38
nonadecane	1.09
henicosane	0.79
ethylene	2.18
1-propene	1.31
**sum**	**20.00**

Table S9 | Detailed composition of experiment simulating Future City in the CHANEL campaign.

Contribution of different emission sources is defined by Stockwell *et al.*[16]

**VCP**	
**Compound of Injection**	**Total Emissions (wt, %)**
Limonene	1.77
propylene glycol	6.62
a-pinene	0.25
b-pinene	0.25
ethanol	17.53
ethylene glycol	1.38
D5 siloxane	2.12
triethanolamine	0.22
n-dodecane	9.00
i-propyl alcohol	10.22
toluene	1.98
m-xylene	3.08
carbitol	0.34
tetrahydrofuran	0.51
ethanolamine	0.48
2-butoxyethanol	0.42
octadecane	0.54
2-methylpropane	5.86
**sum**	**62.56**
**Cooking**	
**Compound of Injection**	**Total Emissions (wt, %)**
acetaldehyde	2.61
propanal	4.33
heptanal	1.59
hexadienal	3.12
pentenal	1.57
octenal	0.79
nonanal	0.78
decanal	1.16
limonene	0.37
ethanol	5.01
tridecanal	0.07
**sum**	**21.38**
**Biogenic**	
**Compound of Injection**	**Total Emissions (wt, %)**
Isoprene	6.75
a-pinene	3.86
limonene	1.45
a-cedrene	1.12
camphene	0.80
eucalyptol	0.80
b-myrcene	0.64
b-pinene	0.64

**sum**	**16.06**

Table S10 | Summary of experiments performed during the SAPHIR CHANEL campaign.

Overview of all 27 experiments conducted in the SAPHIR chamber during the CHANEL campaign, including experiment type, main oxidants, and duration. Experiments covered a range of urban-representative mixtures, NO regimes, and oxidant conditions (daytime OH, nighttime NO_3, and ozonolysis).

Experiment Nr	Experiment Date	Experiment	Main Oxidants	Duration (h)
1	01.07.2024	Limonene Daytime Oxidation at Low NO	OH radicals	7.5
2	03.07.2024	Limonene Nighttime Oxidation	NO_3 radicals	13
3	04.07.2024	VCPs Daytime Oxidation at Low NO	OH radicals	5.5
4	05.07.2024	VCPs Nighttime Oxidation	NO_3 radicals	20
5	08.07.2024	VCPs Daytime Oxidation at Medium NO	OH radicals	7
6	09.07.2024	VCPs Daytime Oxidation at High NO	OH radicals	5
7	10.07.2024	Gasoline Daytime Oxidation at High NO	OH radicals	6
8	11.07.2024	Gasoline Daytime Oxidation at Medium NO	OH radicals	7
9	15.07.2024	Cooking Daytime Oxidation at Medium NO	OH radicals	6
10	16.07.2024	Cooking Daytime Oxidation at Low NO	OH radicals	3.5
11	17.07.2024	Diesel Daytime Oxidation at Medium NO	OH radicals	5.5
12	18.07.2024	Diesel Daytime Oxidation at High NO	OH radicals	6.5
13	22.07.2024	LA Anthropogenic Daytime Oxidation at Medium NO	OH radicals	6
14	23.07.2024	LA Anthropogenic Nighttime Oxidation	NO_3 radicals	7.5
15	25.07.2024	LA Anthropogenic Nighttime to Daytime Oxidation	NO_3 radicals, OH radicals	7
16	26.07.2024-27.07.2024	LA Anthropogenic+Biogenic Nighttime to Daytime Oxidation	NO_3 radicals, OH radicals	28
17	28.07.2024	Background Experiment without seeds	OH radicals	5
18	29.07.2024	LA Anthropogenic+Biogenic Daytime Oxidation at Medium NO	OH radicals	7.5
19	30.07.2024	Global Anthropogenic Daytime Oxidation at Medium NO	OH radicals	7
20	31.07.2024-01.08.2024	LA Anthropogenic+Biogenic Nighttime Oxidation[*]	NO_3 radicals, OH radicals	22
21	02.08.2024	Future City at Low NO	OH radicals	6
22	04.08.2024	Background Experiment + seeds	OH radicals	5
23	05.08.2024	LA Anthropogenic+Biogenic+O_3 Daytime Oxidation at Low NO	OH radicals	9
24	06.08.2024	LA Anthropogenic+Biogenic+O_3+NO_2 Daytime Oxidation at High NO	OH radicals	7.5
25	07.08.2024	Background Experiment + seeds + CO	OH radicals	2.5
26	08.08.2024	Limonene Ozonolysis	O_3	22
27	13.08.2024	Limonene Ozonolysis+CO	O_3	13

[*] The VCP mixture in experiment 20 excluded carbitol, tetrahydrofuran, ethanolamine, 2-butoxyethanol, and octadecane.

935 **Table S11 | Average NO, NO_2, NO_x, and O_3 concentration, temperature (T) and relative humidity (RH) of each experiment during the CHANEL campaign.**

Overview of all 27 experiments conducted in the SAPHIR chamber during the CHANEL campaign, including experiment type, average concentration of NO, NO_2, NO_x, and O_3, and average temperature (T) and relative humidity (RH) during the experiment.

Experiment Nr	Experiment Date	Experiment	Average NO (ppbv)	Average NO_2 (ppbv)	Average NO_x (ppbv)	Average O_3 (ppbv)	Average T (°C)	Average RH (%)
1	01.07.2024	Limonene Daytime Oxidation at Low NO	0.19±0.10	2.2±0.7	2.3±0.8	74.5±5.6	25.6±2.5	40±8
2	03.07.2024	Limonene Nighttime Oxidation	0	11.1±2.3	11.1±2.3	10.6±3.4	17.3±1.1	21.7±3.5
3	04.07.2024	VCPs Daytime Oxidation at Low NO	0.16±0.06	2.2±0.8	2.4±0.8	103±28	28.3±3.9	29±8
4	05.07.2024	VCPs Nighttime Oxidation	0	10.2±3.0	10.2±3.0	11.5±3.3	20.2±0.9	41±7
5	08.07.2024	VCPs Daytime Oxidation at Medium NO	0.27±0.09	1.8±0.6	2.1±0.6	33±19	29.9±1.6	23.1±3.8
6	09.07.2024	VCPs Daytime Oxidation at High NO	1.0±1.5	4.3±0.8	5.3±0.9	67±36	41.5±3.3	21.5±4.5
7	10.07.2024	Gasoline Daytime Oxidation at High NO	0.9±1.3	3.3±0.7	4.2±1.0	51±27	35.0±3.4	22.4±5.5
8	11.07.2024	Gasoline Daytime Oxidation at Medium NO	0.27±0.07	1.6±0.6	1.9±0.6	37±23	34.0±3.6	22±6
9	15.07.2024	Cooking Daytime Oxidation at Medium NO	0.24±0.06	1.3±0.6	1.6±0.6	27±17	35.0±3.5	22±6
10	16.07.2024	Cooking Daytime Oxidation at Low NO	0.07±0.04	1.0±0.5	1.1±0.5	104±26	28.1±2.3	37.0±6.2
11	17.07.2024	Diesel Daytime Oxidation at Medium NO	0.31±0.09	1.2±0.5	1.5±0.5	14±9	30.2±2.4	29.4±5.3
12	18.07.2024	Diesel Daytime Oxidation at High NO	1.1±1.4	2.8±0.6	3.9±1.1	43±23	34.9±3.3	18.9±4.9
13	22.07.2024	LA Anthropogenic Daytime Oxidation at Medium NO	0.22±0.05	1.3±0.5	1.6±0.5	29±19	33.2±3.6	27±8
14	23.07.2024	LA Anthropogenic Nighttime Oxidation	0	12.2±2.2	12.2±2.2	14.0±3.3	26.4±0.9	33.3±4.7
15	25.07.2024	LA Anthropogenic Nighttime to Daytime Oxidation	0.5±0.9	9.7±4.5	10.2±4.3	48±35	31.1±4.3	22±7
16	26.07.2024-27.07.2024	LA Anthropogenic+Biogenic Nighttime to Daytime Oxidation	0.1±0.4	13.1±5.1	13.3±4.9	12±7	23.2±3.4	25.6±4.8
17	28.07.2024	Background Experiment	0.58±0.16	2.4±1.0	3.0±1.1	27±17	31.6±3.9	31±9
18	29.07.2024	LA Anthropogenic+Biogenic Daytime Oxidation at Medium NO	0.15±0.03	1.1±0.3	1.2±0.3	30±19	34.0±4.7	19±7
19	30.07.2024	Global Anthropogenic Daytime Oxidation at Medium NO	0.18±0.04	1.7±0.6	1.8±0.6	39±25	39.5±4.7	20±7

20	31.07.2024-01.08.2024	LA Anthropogenic+Biogenic Nighttime Oxidation*	0	16.9±4.4	16.9±4.4	17±5	23.4±1.7	39±8
21	02.08.2024	Future City at Low NO	0.13±0.04	1.5±0.6	1.6±0.6	54±14	33.1±4.1	25±8
22	04.08.2024	Background Experiment + seeds	0.81±0.29	1.3±0.7	2.1±0.9	7±4	26.9±1.2	43.8±5.5
23	05.08.2024	LA Anthropogenic+Biogenic $+O_3$ Daytime Oxidation at Low NO	0.11±0.04	1.5±0.5	1.6±0.6	62±14	34.9±4.4	21±7
24	06.08.2024	LA Anthropogenic+Biogenic $+O_3+NO_2$ Daytime Oxidation at High NO	0.2±0.4	2.9±0.5	3.1±0.7	94±21	36.4±3.0	21.0±5.5
25	07.08.2024	Background Experiment + seeds + CO	0.15±0.08	0.4±0.3	0.6±0.3	0.9±0.9	23.9±0.8	57.6±3.1
26	08.08.2024	Limonene Ozonolysis	0	0.03±0.01	0.03±0.01	62±12	24.3±3.1	40±9
27	13.08.2024	Limonene Ozonolysis+CO	0	0.10±0.01	0.09±0.01	67±13	31.5±4.9	35±8

Table S12 | Average OH, HO_2, and RO_2 concentration of each experiment, and total OH reactivity measured at the injection mask during the CHANEL campaign.

Experiment Nr	Experiment Date	Average OH (*10^6 molecule*cm^{-3})	Average HO_2 (*10^8 molecule*cm^{-3})	Average RO_2 (*10^8 molecule*cm^{-3})	Total OH reactivity measured at the injection mask (s^{-1})
1	01.07.2024	5.1±2.5	4.8±1.4	3.9±1.1	NaN
2	03.07.2024	0.54±0.49	2.9±1.0	2.1±0.8	NaN
3	04.07.2024	2.1±1.2	8.3±2.0	11±3	45.7
4	05.07.2024	0.77±0.59	0.81±0.23	1.45±0.3	28.5
5	08.07.2024	2.0±1.1	8.3±1.9	8.4±2.0	47.7
6	09.07.2024	NaN	NaN	NaN	43.7
7	10.07.2024	NaN	NaN	NaN	18.2
8	11.07.2024	5.5±2.3	8.7±2.0	6.6±1.6	21.7
9	15.07.2024	NaN	NaN	NaN	28.3
10	16.07.2024	NaN	NaN	NaN	27.4
11	17.07.2024	NaN	NaN	NaN	10.6
12	18.07.2024	NaN	NaN	NaN	14.1
13	22.07.2024	NaN	NaN	NaN	57.8
14	23.07.2024	NaN	NaN	NaN	74.4
15	25.07.2024	NaN	NaN	NaN	NaN
16	26.07.2024-27.07.2024	0.78±0.56	2.3±0.48	4.7±1.1	102.9
17	28.07.2024	7.6±2.8	4.7±0.67	3.0±0.57	NaN
18	29.07.2024	1.6±1.1	9.5±2.5	9.6±2.6	95.0
19	30.07.2024	1.7±1.0	12±4	11±3	87.1
20	31.07.2024-01.08.2024	NaN	1.9±0.72	4.0±1.3	103.2
21	02.08.2024	NaN	11±1.8	11±1.7	NaN
22	04.08.2024	5.0±2.0	1.7±0.53	1.3±0.27	79.9
23	05.08.2024	NaN	11±2.8	10±2.4	82.1
24	06.08.2024	NaN	14±3	11±2	97.6
25	07.08.2024	NaN	3.9±1.2	1.5±0.37	NaN
26	08.08.2024	NaN	NaN	NaN	NaN
27	13.08.2024	NaN	NaN	NaN	NaN

Table S13 | Signals of C_4 VOCs that were observed over LA during the AEROMMA campaign but missing in the CHANEL LA simulation (experiment 24).

Formulas of the compounds	Averaged signals measured in LA urban air (cps)	Normalized total VOC signals (%)
C_4H_4O	5	0.05
$C_4H_4O_6$	3	0.02
C_4H_7N	6	0.05
$C_4H_9N_3O$	4	0.04
$C_4H_{10}N_2O_4$	6	0.05
$C_4H_{10}O$	10	0.09
$C_4H_{10}O_6$	2	0.01

References

(1) Lyu, C.; Harkins, C.; Li, M.; Mueller, K.; Prothero, J.; Verreyken, B.; Miller, J. B.; Lehman, S. J.; Peischl, J.; Gilman, J. B.; Warneke, C.; Coggon, M. M.; Stockwell, C. E.; Brown, S. S.; Zuraski, K.; Lamplugh, A.; Gurney, K. R.; Gawuc, L.; Dass, P.; Hoesly, R. M.; Smith, S. J.; Oda, T.; Hutyra, L. R.; Gately, C. K.; Granier, C.; Whetstone, J. R.; Mcdonald, B. C. Development of the United States GReenhouse Gas and Air Pollutants Emissions System (GRA2PES). *J. Geophys. Res. Atmos.* **2025**, *2* (x), 1–24. https://doi.org/10.1029/2025JD043597.

(2) Atkinson, R.; Arey, J. Atmospheric Degradation of Volatile Organic Compounds. *Chem. Rev.* **2003**, *103* (3), 4605–4638. https://doi.org/10.1021/cr0206420.

(3) Bowman, J. H.; Barket Dennis J.; Shepson, P. B. Atmospheric Chemistry of Nonanal. *Environ. Sci. Technol.* **2003**, *37* (10), 2218–2225. https://doi.org/10.1021/es026220p.

(4) Atkinson, R.; Arey, J.; Aschmann, S. M.; Corchnoy, S. B.; Shu, Y.; Tuazon, E. C.; Atkinson, R.; Carter, W. P. L. Rate Constants for the Gas-Phase Reactions of Cis-3-Hexen-1-Ol, Cis-3-Hexenylacetate, Trans-2-Hexenal, and Linalool with OH and NO3 Radicals and O3 at 296 ± 2 K, and OH Radical Formation Yields from the O3 Reactions. *Int. J. Chem. Kinet.* **1995**, *27* (10), 941–955. https://doi.org/https://doi.org/10.1002/kin.550271002.

(5) Dagaut, P.; Wallington, T. J.; Liu, R.; Kurylo, M. J. A Kinetic Investigation of the Gas-Phase Reactions of Hydroxyl Radicals with Cyclic Ketones and Diones: Mechanistic Insights. *J. Phys. Chem.* **1988**, *92* (15), 4375–4377. https://doi.org/10.1021/j100326a026.

(6) Teruel, M. A.; Benitez-Villalba, J.; Caballero, N.; Blanco, M. B. Gas-Phase Oxidation of Methyl Crotonate and Ethyl Crotonate. Kinetic Study of Their Reactions toward OH Radicals and Cl Atoms. *J. Phys. Chem. A* **2012**, *116* (24), 6127–6133. https://doi.org/10.1021/jp2113889.

(7) IUPAC. *Atmospheric Chemical Kinetic Data Evaluation*. https://iupac.aeris-data.fr/.

(8) NIST. *NIST Chemical Kinetics Database Standard Reference Database 17, Version 7.1 (Web Version), Release 1.6.8 Data Version 2025*. https://kinetics.nist.gov/kinetics/.

(9) Cappa, C. D.; Wilson, K. R. Multi-Generation Gas-Phase Oxidation, Equilibrium Partitioning, and the Formation and Evolution of Secondary Organic Aerosol. *Atmos. Chem. Phys.* **2012**, *12* (20), 9505–9528. https://doi.org/10.5194/acp-12-9505-2012.

(10) Pye, H. O. T.; Place, B. K.; Murphy, B. N.; Seltzer, K. M.; D'Ambro, E. L.; Allen, C.; Piletic, I. R.; Farrell, S.; Schwantes, R. H.; Coggon, M. M.; Saunders, E.; Xu, L.; Sarwar, G.; Hutzell, W. T.; Foley, K. M.; Pouliot, G.; Bash, J.; Stockwell, W. R. Linking Gas, Particulate, and Toxic Endpoints to Air Emissions in the Community Regional Atmospheric Chemistry Multiphase Mechanism (CRACMM). *Atmos. Chem. Phys.* **2023**, *23* (9), 5043–5099. https://doi.org/10.5194/acp-23-5043-2023.

(11) Guo, Z.; Chen, X.; Wu, D.; Huo, Y.; Cheng, A.; Liu, Y.; Li, Q.; Chen, J. Higher Toxicity of Gaseous Organics Relative to Particulate Matters Emitted from Typical Cooking Processes. *Environ. Sci. Technol.* **2023**, *57* (44), 17022–17031. https://doi.org/10.1021/acs.est.3c05425.

(12) Warneke, C.; Schwantes, R.; Veres, P. R.; Rollins, A.; Baidar, S.; Brewer, W. A.; Senff, C.; Langford, A.; Aikin, K.; Frost, G.; Fahey, D.; Judd, L.; Lefer, B.; Pierce, R. B.; Kondragunta, S.; Stockwell, C.; Gentner, D.; Lambe, Andrew T. Dylan B. Millet, Delphine Farmer, Nga Lee Ng, Jennifer Kaiser, C. Y.; John E. Mak, Glenn M. Wolfe, John Sullivan, Kimberly Mueller, Anna Karion, Lukas Valin, Mikael Witte, Lynn M. Russell, Xinrong Ren, Russell Dickerson, Peter Decarlo, B. M.; Brown, S. S. *The AEROMMA 2023 Experiment (Atmospheric Emissions and Reactions Observed from Megacities to Marine Areas)*. https://csl.noaa.gov/projects/aeromma/whitepaper.pdf.

(13) Roska, M.; Stockwell, C. E.; Coggon, M. M.; Bates, K. H.; Xu, L.; Womack, C. C.; Chace, W. S.; Wu, Y.; Tillmann, R.; Wesolek, C.; Pfannerstill, E. Y.; Rohrer, F.; Tsimpidi, A. P.; Kar, V. A.; Gkatzelis, G. I. Beyond Targeted

Calibrations: Comprehensive Quantification of Oxygenated Organic Compounds in Urban Atmospheres. *Environ. Sci. Technol.* **2026**, *60* (34), 24227–24238.

(14) Robinson, M. A.; Coggon, M. M.; Bates, K. H.; Peischl, J.; Jernigan, C. M.; Novak, G.; Thakali, S.; Roberts, J. M.; Neuman, J. A.; Veres, P. R.; Zuraski, K.; Waxman, E. M.; Chace, W. S.; Rollins, A. W.; Treadaway, V.; Selby, M.; Francoeur, C.; Gilman, J. B.; Liu, S.; Delaria, E. R.; Sebol, A. E.; Desai, N. S.; Kaiser, J.; Kautzman, K. E.; Clair, J. M. S.; Wolfe, G. M.; Xu, L.; Stockwell, C. E.; Warneke, C.; Huynh, H. N.; Lyu, M.; Ahern, A.; Brock, C. A.; Piasecki, A.; Albertin, S.; Middlebrook, A. M.; Sullivan, A. P.; Schwantes, R.; Abad, G. G.; Nowlan, C. R.; Liu, X. Fate of Isoprene Peroxy Radical Constrains the Urban Photochemical Regime. *Sci. Adv.* **2026**, 1–13. https://doi.org/10.1126/sciadv.aea6509.

(15) McDonald, B. C.; De Gouw, J. A.; Gilman, J. B.; Jathar, S. H.; Akherati, A.; Cappa, C. D.; Jimenez, J. L.; Lee-Taylor, J.; Hayes, P. L.; McKeen, S. A.; Cui, Y. Y.; Kim, S. W.; Gentner, D. R.; Isaacman-VanWertz, G.; Goldstein, A. H.; Harley, R. A.; Frost, G. J.; Roberts, J. M.; Ryerson, T. B.; Trainer, M. Volatile Chemical Products Emerging as Largest Petrochemical Source of Urban Organic Emissions. *Science (80-.).* **2018**, *359* (6377), 760–764. https://doi.org/10.1126/science.aaq0524.

(16) Stockwell, C. E.; Coggon, M. M.; Schwantes, R. H.; Harkins, C.; Verreyken, B.; Lyu, C.; Zhu, Q.; Xu, L.; Gilman, J. B.; Lamplugh, A.; Peischl, J.; Robinson, M. A.; Veres, P. R.; Li, M.; Rollins, A. W.; Zuraski, K.; Baidar, S.; Liu, S.; Kuwayama, T.; Brown, S. S.; McDonald, B. C.; Warneke, C. Urban Ozone Formation and Sensitivities to Volatile Chemical Products, Cooking Emissions, and NOx across the Los Angeles Basin. *Atmosphecic Chem. Phys.* **2025**, *2024* (x), 1–24.

(17) Coggon, M. M.; Stockwell, C. E.; Xu, L.; Peischl, J.; Gilman, J. B.; Lamplugh, A.; Bowman, H. J.; Aikin, K.; Harkins, C.; Zhu, Q.; Schwantes, R. H.; He, J.; Li, M.; Seltzer, K.; Mcdonald, B.; Warneke, C.; Agency, U. S. E. P.; Park, T.; Sciences, P.; Stockwell, C. E.; Xu, L.; Peischl, J.; Gilman, J. B.; Lamplugh, A.; Bowman, H. J.; Aikin, K.; Harkins, C.; Zhu, Q.; Schwantes, R. H.; He, J.; Li, M.; Seltzer, K.; Mcdonald, B.; Warneke, C. Contribution of Cooking Emissions to the Urban Volatile Organic Compounds in Las Vegas, NV. *Atmos. Chem. Phys.* **2024**, *24* (7), 4289–4304. https://doi.org/10.5194/acp-24-4289-2024.

(18) Mcdonald, B. C.; Mckeen, S. A.; Cui, Y. Y.; Ahmadov, R.; Kim, S.; Frost, G. J.; Pollack, I. B.; Peischl, J.; Ryerson, T. B.; Holloway, J. S.; Graus, M.; Warneke, C.; Gilman, J. B.; Gouw, J. A. De; Kaiser, J.; Keutsch, F. N.; Hanisco, T. F.; Wolfe, G. M.; Trainer, M. Modeling Ozone in the Eastern U.S. Using a Fuel-Based Mobile Source Emissions Inventory. *Environ. Sci. Technol.* **2018**. https://doi.org/10.1021/acs.est.8b00778.

(19) He, J.; Harkins, C.; O'Dell, K.; Li, M.; Francoeur, C.; Aikin, K. C.; Anenberg, S.; Baker, B.; Brown, S. S.; Coggon, M. M.; Frost, G. J.; Gilman, J. B.; Kondragunta, S.; Lamplugh, A.; Lyu, C.; Moon, Z.; Pierce, B. R.; Schwantes, R. H.; Stockwell, C. E.; Warneke, C.; Yang, K.; Nowlan, C. R.; González Abad, G.; McDonald, B. C. COVID-19 Perturbation on US Air Quality and Human Health Impact Assessment. *PNAS Nexus* **2024**, *3* (1), pgad483. https://doi.org/10.1093/pnasnexus/pgad483.

(20) Cho, C.; Fuchs, H.; Hofzumahaus, A.; Holland, F.; Bloss, W. J.; Bohn, B.; Dorn, H. P. H.-P.; Glowania, M.; Hohaus, T.; Liu, L.; Monks, P. S.; Niether, D.; Rohrer, F.; Sommariva, R.; Tan, Z.; Tillmann, R.; Kiendler-Scharr, A.; Wahner, A.; Novelli, A. Experimental Chemical Budgets of OH, HO2, and RO2 Radicals in Rural Air in Western Germany during the JULIAC Campaign 2019. *Atmos. Chem. Phys.* **2023**, *23* (3), 2003–2033. https://doi.org/10.5194/acp-23-2003-2023.

(21) Färber, M.; Fuchs, H.; Bohn, B.; Carlsson, P. T. M.; Gkatzelis, G. I.; Marcillo Lara, A. C.; Rohrer, F.; Vereecken, L.; Wedel, S.; Wahner, A.; Novelli, A. Effect of the Alkoxy Radical Chemistry on the Ozone Formation from Anthropogenic Organic Compounds Investigated in Chamber Experiments. *ACS ES&T Air* **2024**. https://doi.org/10.1021/acsestair.4c00064.

(22) Reinecke, T.; Leiminger, M.; Jordan, A.; Wisthaler, A.; Müller, M. Ultrahigh Sensitivity PTR-MS Instrument with a Well-Defined Ion Chemistry. *Anal. Chem.* **2023**, *95* (32), 11879–11884. https://doi.org/10.1021/acs.analchem.3c02669.

(23) Fuchs, H.; Bohn, B.; Hofzumahaus, A.; Holland, F.; Lu, K. D.; Nehr, S.; Rohrer, F.; Wahner, A. Detection of HO2 by Laser-Induced Fluorescence: Calibration and Interferences from RO2 Radicals. *Atmos. Meas. Tech.* **2011**, *4* (6), 1209–1225. https://doi.org/10.5194/amt-4-1209-2011.

(24) Fuchs, H.; Novelli, A.; Rolletter, M.; Hofzumahaus, A.; Pfannerstill, E. Y.; Kessel, S.; Edtbauer, A.; Williams, J.; Michoud, V.; Dusanter, S.; Locoge, N.; Zannoni, N.; Gros, V.; Truong, F.; Sarda-Esteve, R.; Cryer, D. R.; Brumby, C. A.; Whalley, L. K.; Stone, D.; Seakins, P. W.; Heard, D. E.; Schoemaecker, C.; Blocquet, M.; Coudert, S.; Batut, S.; Fittschen, C.; Thames, A. B.; Brune, W. H.; Ernest, C.; Harder, H.; Muller, J. B. A.; Elste, T.; Kubistin, D.; Andres, S.; Bohn, B.; Hohaus, T.; Holland, F.; Li, X.; Rohrer, F.; Kiendler-Scharr, A.; Tillmann, R.; Wegener, R.; Yu, Z.; Zou, Q.; Wahner, A. Comparison of OH Reactivity Measurements in the Atmospheric Simulation Chamber SAPHIR. *Atmos. Meas. Tech.* **2017**, *10* (10), 4023–4053. https://doi.org/10.5194/amt-10-4023-2017.

(25) Canagaratna, M. R.; Jayne, J. T.; Jimenez, J. L.; Allan, J. D.; Alfarra, M. R.; Zhang, Q.; Onasch, T. B.; Drewnick, F.; Coe, H.; Middlebrook, A.; Delia, A.; Williams, L. R.; Trimborn, A. M.; Northway, M. J.; DeCarlo, P. F.; Kolb, C. E.; Davidovits, P.; Worsnop, D. R. Chemical and Microphysical Characterization of Ambient Aerosols with the Aerodyne Aerosol Mass Spectrometer. *Mass Spectrom. Rev.* **2007**, *26* (2), 185–222. https://doi.org/10.1002/mas.20115.

(26) Canagaratna, M. R.; Jimenez, J. L.; Kroll, J. H.; Chen, Q.; Kessler, S. H.; Massoli, P.; Hildebrandt Ruiz, L.; Fortner, E.; Williams, L. R.; Wilson, K. R.; Surratt, J. D.; Donahue, N. M.; Jayne, J. T.; Worsnop, D. R. Elemental Ratio Measurements of Organic Compounds Using Aerosol Mass Spectrometry: Characterization, Improved Calibration, and Implications. *Atmos. Chem. Phys.* **2015**, *15* (1), 253–272. https://doi.org/10.5194/acp-15-253-2015.

(27) Horbanski, M.; Pöhler, D.; Lampel, J.; Platt, U. The ICAD (Iterative Cavity-Enhanced DOAS) Method. *Atmos. Meas. Tech.* **2019**, *12* (6), 3365–3381. https://doi.org/10.5194/amt-12-3365-2019.

(28) Bohn, B.; Lohse, I. Calibration and Evaluation of CCD Spectroradiometers for Ground-Based and Airborne Measurements of Spectral Actinic Flux Densities. *Atmos. Meas. Tech.* **2017**, *10* (9), 3151–3174. https://doi.org/10.5194/amt-10-3151-2017.

(29) Bohn, B.; Lohse, I. Optical Receiver Characterizations and Corrections for Ground-Based and Airborne Measurements of Spectral Actinic Flux Densities. *Atmos. Meas. Tech.* **2023**, *16* (2), 209–233. https://doi.org/10.5194/amt-16-209-2023.

(30) Bohn, B.; Zilken, H. Model-Aided Radiometric Determination of Photolysis Frequencies in a Sunlit Atmosphere Simulation Chamber. *Atmos. Chem. Phys.* **2005**, *5* (1), 191–206. https://doi.org/10.5194/acp-5-191-2005.

(31) Bohn, B.; Rohrer, F.; Brauers, T.; Wahner, A. Actinometric Measurements of NO2 Photolysis Frequencies in the Atmosphere Simulation Chamber SAPHIR. *Atmos. Chem. Phys.* **2005**, *5* (2), 493–503. https://doi.org/10.5194/acp-5-493-2005.

(32) Kenagy, H. S.; Heald, C. L.; Tahsini, N.; Goss, M. B.; Kroll, J. H. Can We Achieve Atmospheric Chemical Environments in the Laboratory? An Integrated Model-Measurement Approach to Chamber SOA Studies. *Sci. Adv.* **2024**, *10* (37), 1–29. https://doi.org/10.1126/sciadv.ado1482.

(33) Griffin R. J. , Cocker III D. R. , Flagan R. C., S. J. H. Organic Aerosol Formation from the Oxidation of Biogenic Hydrocarbons. *J. Geophys. Res. Atmos.* **1999**, *104*, 3555–3567.

(34) Kroll, J. H.; Ng, N. L.; Murphy, S. M.; Flagan, R. C.; Seinfeld, J. H. Secondary Organic Aerosol Formation from Isoprene Photooxidation under High-NO Conditions. *Geophys. Res. Lett.* **2005**, *32* (18). https://doi.org/https://doi.org/10.1029/2005GL023637.

(35) Ng, N. L.; Chhabra, P. S.; Chan, A. W. H.; Surratt, J. D.; Kroll, J. H.; Kwan, A. J.; McCabe, D. C.; Wennberg, P. O.;

Sorooshian, A.; Murphy, S. M.; Dalleska, N. F.; Flagan, R. C.; Seinfeld, J. H. Effect of NOx Level on Secondary Organic Aerosol (SOA) Formation from the Photooxidation of Terpenes. *Atmos. Chem. Phys.* **2007**, *7* (19), 5159–5174. https://doi.org/10.5194/acp-7-5159-2007.

(36) Shilling, J. E.; Chen, Q.; King, S. M.; Rosenoern, T.; Kroll, J. H.; Worsnop, D. R.; McKinney, K. A.; Martin, S. T. Particle Mass Yield in Secondary Organic Aerosol Formed by the Dark Ozonolysis of α-Pinene. *Atmos. Chem. Phys.* **2008**, *8* (7), 2073–2088. https://doi.org/10.5194/acp-8-2073-2008.

(37) Ahlberg, E.; Eriksson, A.; Brune, W. H.; Roldin, P.; Svenningsson, B. Effect of Salt Seed Particle Surface Area, Composition and Phase on Secondary Organic Aerosol Mass Yields in Oxidation Flow Reactors. *Atmos. Chem. Phys.* **2019**, *19* (4), 2701–2712. https://doi.org/10.5194/acp-19-2701-2019.

(38) Li, Z.; Buchholz, A.; Barreira, L. M. F.; Ylisirniö, A.; Hao, L.; Pullinen, I.; Schobesberger, S.; Virtanen, A. Isothermal Evaporation of α-Pinene Secondary Organic Aerosol Particles Formed under Low NOx and High NOx Conditions. *Atmos. Chem. Phys.* **2023**, *23* (1), 203–220. https://doi.org/10.5194/acp-23-203-2023.

(39) Nie, W.; Yan, C.; Yang, L.; Roldin, P.; Liu, Y.; Vogel, A. L.; Molteni, U.; Stolzenburg, D.; Finkenzeller, H.; Amorim, A.; Bianchi, F.; Curtius, J.; Dada, L.; Draper, D. C.; Duplissy, J.; Hansel, A.; He, X.-C.; Hofbauer, V.; Jokinen, T.; Kim, C.; Lehtipalo, K.; Nichman, L.; Mauldin, R. L.; Makhmutov, V.; Mentler, B.; Mizelli-Ojdanic, A.; Petäjä, T.; Quéléver, L. L. J.; Schallhart, S.; Simon, M.; Tauber, C.; Tomé, A.; Volkamer, R.; Wagner, A. C.; Wagner, R.; Wang, M.; Ye, P.; Li, H.; Huang, W.; Qi, X.; Lou, S.; Liu, T.; Chi, X.; Dommen, J.; Baltensperger, U.; El Haddad, I.; Kirkby, J.; Worsnop, D.; Kulmala, M.; Donahue, N. M.; Ehn, M.; Ding, A. NO at Low Concentration Can Enhance the Formation of Highly Oxygenated Biogenic Molecules in the Atmosphere. *Nat. Commun.* **2023**, *14* (1), 3347. https://doi.org/10.1038/s41467-023-39066-4.

(40) Kroll, J. H.; Ng, N. L.; Murphy, S. M.; Flagan, R. C.; Seinfeld, J. H. Secondary Organic Aerosol Formation from Isoprene Photooxidation. *Environ. Sci. Technol.* **2006**, *40* (6), 1869–1877. https://doi.org/10.1021/es0524301.

(41) Kaminski, M.; Fuchs, H.; Acir, I.-H.; Bohn, B.; Brauers, T.; Dorn, H.-P.; Häseler, R.; Hofzumahaus, A.; Li, X.; Lutz, A.; Nehr, S.; Rohrer, F.; Tillmann, R.; Vereecken, L.; Wegener, R.; Wahner, A. Investigation of the β-Pinene Photooxidation by OH in the Atmosphere Simulation Chamber SAPHIR. *Atmos. Chem. Phys.* **2017**, *17* (11), 6631–6650. https://doi.org/10.5194/acp-17-6631-2017.

(42) Rohrer, F.; Bohn, B.; Brauers, T.; Brüning, D.; Johnen, F. J.; Wahner, A.; Kleffmann, J. Characterisation of the Photolytic HONO-Source in the Atmosphere Simulation Chamber SAPHIR. *Atmos. Chem. Phys.* **2005**, *5* (8), 2189–2201. https://doi.org/10.5194/acp-5-2189-2005.

(43) Pfannerstill, E. Y.; Arata, C.; Zhu, Q.; Schulze, B. C.; Ward, R.; Woods, R.; Harkins, C.; Schwantes, R. H.; Seinfeld, J. H.; Bucholtz, A.; Cohen, R. C.; Goldstein, A. H. Temperature-Dependent Emissions Dominate Aerosol and Ozone Formation in Los Angeles. *Science* **2024**, *384* (6702), 1324–1329. https://doi.org/10.1126/science.adg8204.
